# Access to Cyclic Monensin Derivatives via a Four-Component Ugi Reaction

**DOI:** 10.1021/acs.joc.6c01246

**Published:** 2026-07-04

**Authors:** Robert Graniczny, Adam Huczyński, Jan Janczak, Dagmara Kłopotowska, Joanna Wietrzyk, Michał Sulik

**Affiliations:** † Department of Medical Chemistry, Faculty of Chemistry, Adam Mickiewicz University, Uniwersytetu Poznańskiego 8, 61−614 Poznań, Poland; ‡ 215275 Institute of Low Temperature and Structure Research, Polish Academy of Sciences, Okólna 2, Wrocław, 50−422, Poland; § Hirszfeld Institute of Immunology and Experimental Therapy, Polish Academy of Sciences, Rudolfa Weigla 12, 53−114 Wrocław, Poland

## Abstract

Herein, we report the application of the Ugi four-component reaction as an efficient and versatile strategy for the synthesis of macrocyclic derivatives of monensin. The approach enables the incorporation of a peptidomimetic linker, significantly modulating the biological and cation complexation properties of the obtained compounds. All derivatives were obtained in crystalline form, allowing unambiguous structural elucidation by single-crystal X-ray diffraction, which revealed macrocyclic architectures stabilized by intramolecular hydrogen bonding.

## Graphical abstract

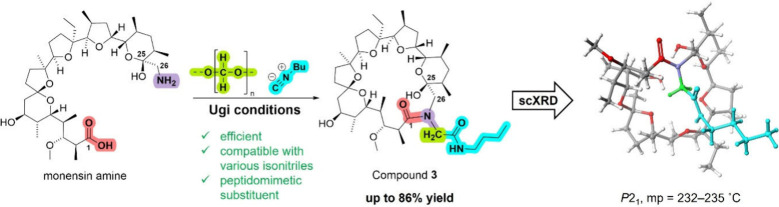

## Introduction

Among the many types of reactions employed in organic chemistry, particular attention should be given to multicomponent reactions (MCRs).^1–5^ These are processes in which three or more starting materials react in a single operation to form a product in which most, if not all, of the atoms from the reactants are incorporated into the final structure.^1,5^ Numerous examples of MCRs involving three components are well-known, such as the Mannich reaction, which enables the synthesis of aminomethylated compounds,^6,7^ and the Strecker reaction, widely used for the preparation of amino acids.^8,9^ In contrast, four-component reactions are considerably less common; among them, the most extensively studied and broadly applied is the Ugi reaction (Scheme 1).^10,11^

**Scheme 1:**
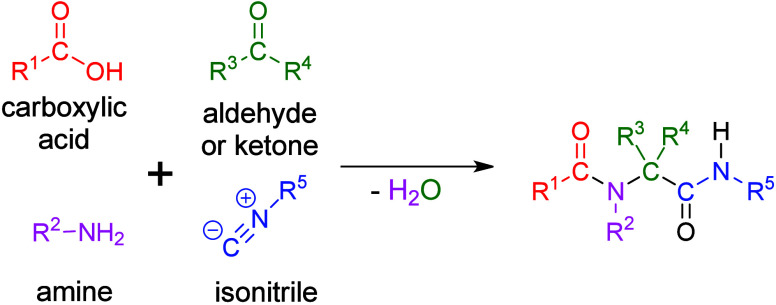
General Scheme of the Four-Component Ugi Reaction

The Ugi four-component condensation (U-4CC) involves the reaction of a carboxylic acid, an amine, an aldehyde or ketone, and an isonitrile, with the latter acting as a key and distinctive reagent in this transformation (Scheme 1).^10,11^ The reaction affords peptidomimetic compounds that are of considerable interest in medicinal chemistry due to their potential bioactivity.^10–16^ Notably, even with a relatively small set of starting materials, the combinatorial nature of this reaction enables the rapid and cost-effective generation of diverse libraries of novel compounds.^17–20^ An interesting and innovative extension of the Ugi reaction in both organic and medicinal chemistry is its application in cyclization processes, which enables the formation of structurally intriguing macrocyclic frameworks.

Numerous examples of such approaches have been reported, in which the use of bifunctional reagents enabled the construction of novel macrocyclic and cage-like architectures.^21–26^ Moreover, this strategy has also been applied to side chain-to-side chain and side chain-to-terminus macrocyclization of peptides, leading to the formation of stable, folded structures.^27^ These observations clearly demonstrate the significance of the Ugi reaction in this context, highlighting its potential for further expansion toward the cyclization of natural product-derived compounds. In this regard, carboxylic ionophore antibiotics, such as monensin, appear to be particularly intriguing targets.

Monensin (**MON**, Figure 1a), like other ionophores, exhibits the ability to coordinate metal cations within a polar pocket and transport them across biological membranes.^28,29^ This polar pocket arises from a specific pseudocyclic conformation of the molecule, stabilized by intramolecular hydrogen bonding (Figure 1a).^28,29^ Motivated by longstanding interest in the properties of monensin, we wished to establish the influence of macrocyclization of the ionophore on its properties. Conversion of the C26 hydroxyl group into an amino functionality enabled an efficient macrocyclization, affording a macrolactam (Figure 1b).^30^ Although this derivative displays reduced biological activity compared to that of **MON**, it retains the ability to coordinate group 1 and 2 metal cations, with the highest affinity observed for sodium, and can also function as an ion-pair receptor.^30^

**Figure 1:**
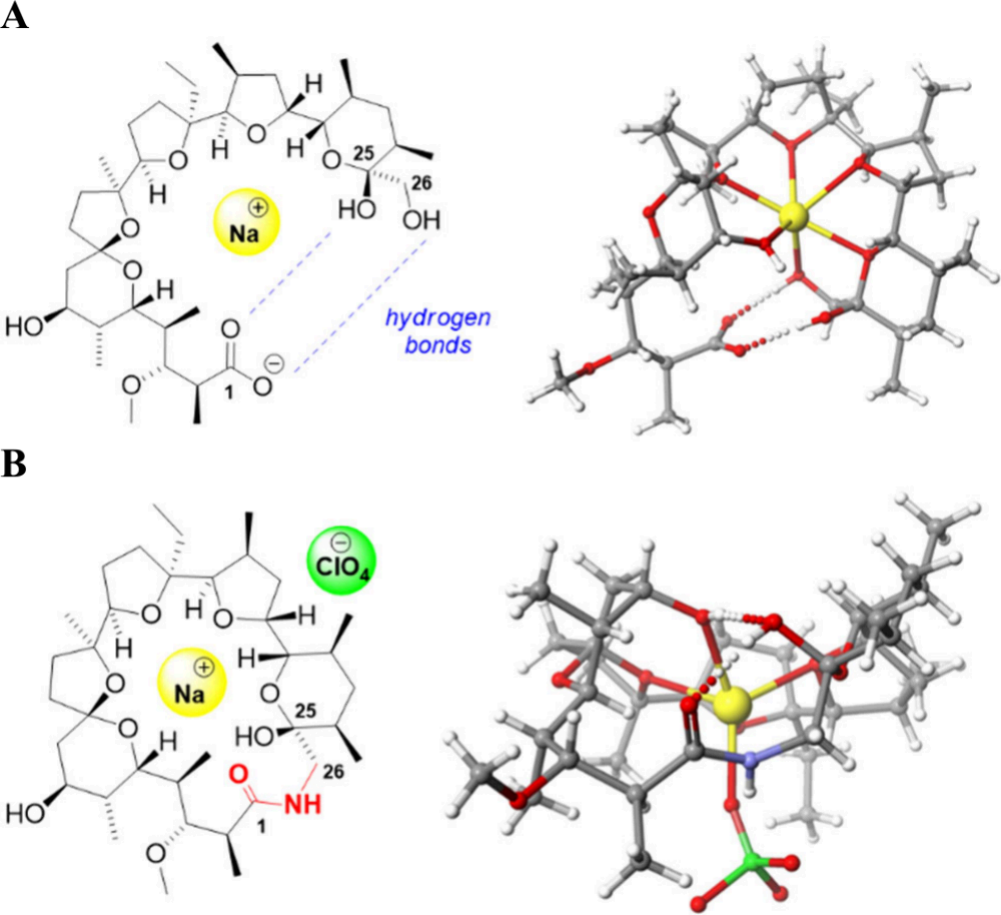
(A) Crystal structure of monensin A sodium salt (based on CCDC 620458); (B) Crystal structure of the cyclic monensin lactam complex with NaClO_4_ (based on CCDC 2391726).

## Results and Discussion

Several strategies for the cyclization of the **MON** scaffold have been reported, including macrolactonization and macrolactamization approaches, based on amino acid incorporation or “click” chemistry.^29–34^ In contrast, the use of the Ugi reaction represents an entirely novel approach to the macrocyclization of this molecule. It offers a particularly attractive strategy for two reasons: (i) the peptidomimetic linker may offer improved physiological compatibility across diverse biological systems, and (ii) depending on the isonitrile employed, a “tail” can be introduced into the macrocyclic structure, providing a convenient handle for further functionalization (scorpion-like macrocycles).^35,36^

To enable the desired macrocyclization, two of the four functional groups required for the Ugi reaction must be present in the substrate. As **MON** contains a carboxylic acid functionality, we attempted to prepare its C26 amino derivative (compound **2**, Scheme 2). This transformation was accomplished via a three-step sequence, comprising tosylation of the hydroxyl group, nucleophilic substitution with sodium azide, and subsequent reduction of the resulting azide under hydrogenation conditions (Scheme 2).^30^ The obtained compound **2** incorporates both carboxyl and amino functionalities within its structure, making it a bifunctional building block for further transformation.

**Scheme 2:**
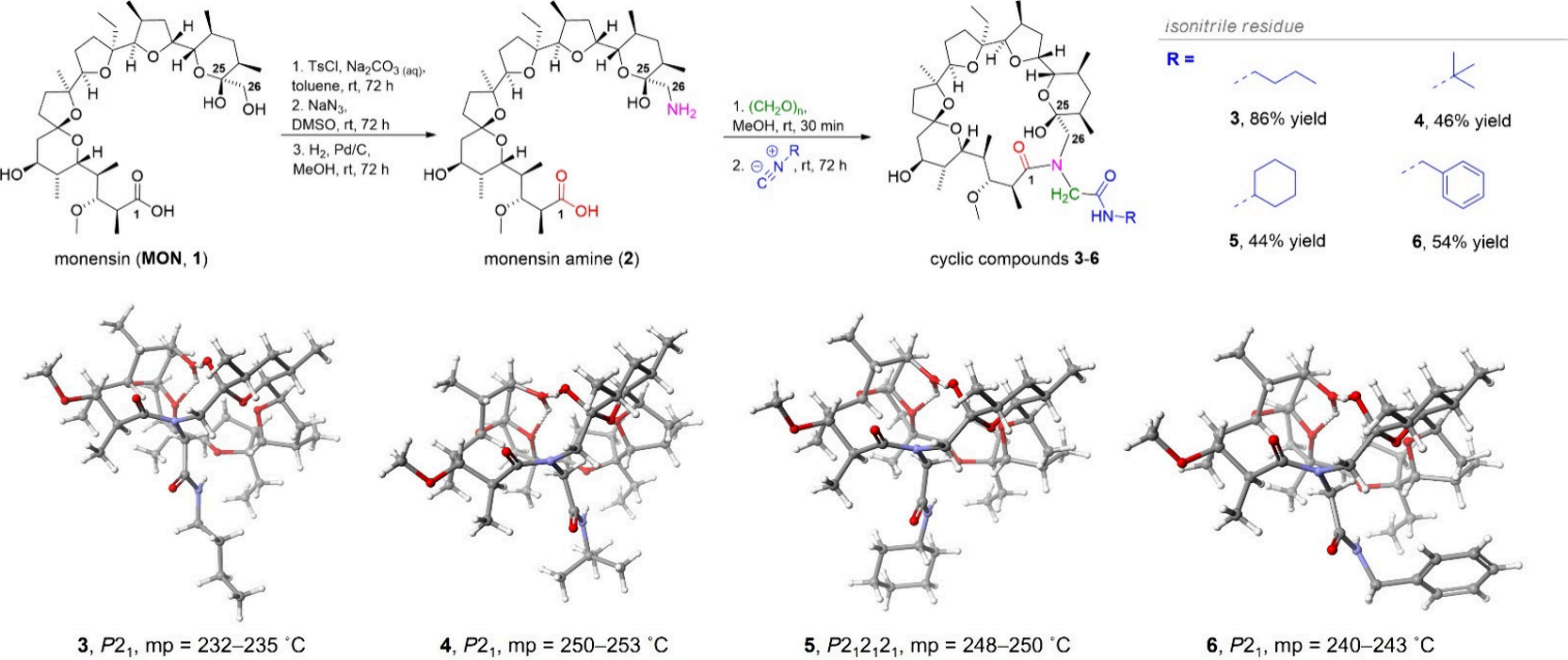
Synthesis of MON Macrocycles Using the Ugi Reaction, as well as Crystal Structures of the Obtained Derivatives

With access to the bifunctional precursor, the selection of an appropriate carbonyl component and isonitrile was essential. As this transformation was being explored for the first time, we opted for paraformaldehyde to avoid an unnecessary structural complexity (Scheme 2). Owing to its minimal steric demand, it readily formed an imine with the C26 amino group of **MON**, constituting the initial step of the Ugi reaction (for this reason, the reaction was initially conducted in the absence of the isonitrile). An additional advantage of paraformaldehyde is the introduction of a methylene unit that does not generate an additional stereogenic center. After imine formation (approximately 30 min), an appropriate isonitrile was added to the reaction mixture (Scheme 2). Four different isonitriles bearing substituents of diverse character (linear aliphatic, branched aliphatic, cyclic aliphatic, and aromatic) were selected to enable preliminary assessment of structure–property relationships, even within a relatively small set of products (Scheme 2). It is worth noting that isonitriles (particularly butyl isonitrile) possess an exceptionally strong and unpleasant odor; therefore, appropriate precautions should be taken when handling these reagents.^37^

The reaction afforded four new macrocyclic derivatives of **MON** in satisfactory yields (44–86%, Scheme 2). Their structures and purities were confirmed by spectroscopic (NMR, FT-IR) and spectrometric (ESI-HRMS) methods. Additionally, all derivatives (**3**–**6**) were obtained in crystalline form, enabling single-crystal X-ray diffraction (scXRD) analysis. Characteristic ^13^C NMR resonances for the monensin-derived and isonitrile-derived carbonyl groups were observed at 179.9–180.0 and 168.1–169.3 ppm, respectively, while the amide proton appeared at 5.87–6.61 ppm in the ^1^H NMR spectra. FT–IR spectra showed two characteristic carbonyl bands (∼1690 and 1655 cm^–1^), and ESI-HRMS analysis confirmed the molecular ions as [M + Na]^+^. Detailed spectroscopic data are provided in the ().

Structural confirmation of the obtained derivatives and insight into their conformational features were achieved by single-crystal X-ray diffraction analysis. Accordingly, all cyclic derivatives of **MON** were characterized using this method. Detailed crystallographic data, refinement parameters, and experimental details are provided in and (). Single crystals of compounds **3**–**6** were obtained by slow crystallization from acetonitrile. Compounds **3**, **4**, and **6** crystallized in the noncentrosymmetric space group *P*2_1_ of the monoclinic system, whereas compound **5** crystallized in the noncentrosymmetric space group *P*2_1_2_1_2_1_ of the orthorhombic system. The asymmetric units of compounds **3**–**6** are shown in Scheme 2.

The molecular structures of all derivatives are closely related; the analysis confirmed their macrocyclic architecture which is further stabilized by intramolecular hydrogen bonding, whose exact geometry is described in (). Representative examples include hydrogen bonds between the hydroxyl group O3–H and the oxygen atom O9, as well as between O9–H and O3, present in all investigated structures. Notably, despite attempts to obtain complexes with sodium (via addition of NaClO_4_ or NaCl) or potassium (via addition of KBF_4_) cations, all compounds were crystallized in their uncoordinated forms.

In our previous studies, we have demonstrated that cyclic derivatives of **MON** are capable of coordinating alkali and alkaline-earth metal cations.^30^ Considering that, for the derivatives obtained in this work, crystalline complexes with sodium cations could not be obtained, we revisited this aspect using electrospray ionization mass spectrometry (ESI-MS). We investigated the complexation ability of the cyclic **MON** derivatives toward mono- (Li^+^, Na^+^, K^+^, Rb^+^, and Cs^+^) and divalent (Mg^2+^, Ca^2+^, Sr^2+^, and Ba^2+^) cations. Surprisingly, the derivatives obtained via the Ugi reaction exhibited significantly lower complexation ability compared to those of the previously reported macrocyclic analogues. The studies revealed that the highest affinity was observed for Na^+^ and Li^+^ cations; notably, compound **3** displayed an even higher affinity toward Li^+^, which is rather uncommon for **MON** derivatives.

Given that **MON** and its derivatives are of significant interest in medicinal chemistry, we considered it essential to establish the effect of application of the Ugi reaction to macrocyclization on the antiproliferative activity of the newly obtained macrocycles. Incorporation of a peptidomimetic linker permitted access to highly active cyclic monensin derivatives (**3** and **5**). Compound **3** retained strong antiproliferative activity (IC_50_ < 5 μM) and high selectivity toward LoVo/DX cells (SI = 14.5). All derivatives overcame drug resistance of LoVo/DX cells (RI < 1.0) and in several cases showed higher selectivity than cisplatin and doxorubicin (Table 1).

**Table 1:** Antiproliferative Activity (IC_50_) (μM) and the Calculated Values of the Selectivity () and Resistance (RI) Index of MON, Its Macrocyclic Derivatives, and Reference Drugs^a^

	Cancer cells									Normal cells
	A549			MCF-7		LoVo		LoVo/DX		BALB/3T3
Compound	IC_50_	SI^b^	IC_50_	SI^b^	IC_50_	SI^b^	IC_50_	SI^b^	RI^c^	IC_50_
monensin, **1**	0.09 ± 0.07	45.9	0.26 ± 0.13	15.9	0.05 ± 0.04	82.6	0.05 ± 0.03	82.6	1.0	4.13 ± 4.04
**3**	3.97 ± 1.48	6.7	4.87 ± 1.12	5.5	1.95 ± 0.39	13.6	1.84 ± 0.39	14.5	0.9	26.60 ± 6.36
**4**	13.37 ± 2.22	5.9	36.98 ± 20.27	2.1	29.60 ± 21.59	2.7	24.50 ± 19.69	3.2	0.8	79.43 ± 26.36
**5**	4.06 ± 2.70	5.0	8.28 ± 5.60	2.4	3.03 ± 1.99	6.6	2.52 ± 1.39	8.0	0.8	20.14 ± 9.62
**6**	20.60 ± 7.86	3.0	33.54 ± 13.20	1.8	14.35 ± 6.48	4.3	12.82 ± 5.48	4.8	0.9	61.82 ± 11.25
										
cisplatin	2.54 ± 0.85	2.4	2.72 ± 0.94	2.2	3.65 ± 1.20	1.7	4.46 ± 1.12	1.4	1.2	6.10 ± 2.35
doxorubicin	0.15 ± 0.11	1.8	0.14 ± 0.05	1.9	0.10 ± 0.03	2.7	3.66 ± 0.83	0.07	36.6	0.27 ± 0.12

a Cisplatin and doxorubicin were used as reference drugs in this study. Panel of cell lines: human lung carcinoma (A549), human breast adenocarcinoma (MCF-7), human colon adenocarcinoma (LoVo), its doxorubicin-resistant subline (LoVo/DX), and normal murine embryonic fibroblasts (BALB/3T3).

b The selectivity index () was calculated as the ratio of the IC_50_ value for the normal cell line BALB/3T3 to the IC_50_ value of a respective cancer cell line.

c The resistance index (RI) was calculated as the ratio of the IC_50_ value of the LoVo/DX cell line to that of the LoVo cell line.

## Conclusions

In summary, we report the application of the Ugi four-component reaction as an efficient and versatile tool for the synthesis of macrocyclic derivatives of monensin, a representative ionophore antibiotic. Notably, the reaction proved to be compatible with a diverse range of isonitriles, affording the desired products in moderate to good yields (44–86%). Furthermore, the use of paraformaldehyde as the carbonyl component eliminated the formation of diastereomeric mixtures, simplifying product profiles and facilitating purification. Structural modification of the ionophore framework was found to significantly influence both, its biological activity and metal cation complexation properties. The structures of the obtained macrocycles were confirmed by spectroscopic and spectrometric methods, as well as by single-crystal X-ray diffraction analysis, which revealed their cyclic architecture further stabilized by a network of intramolecular hydrogen bonds. Notably, the compounds did not crystallize as sodium complexes. Consistently with this observation, complexation studies demonstrated reduced affinity toward alkali and alkaline earth metal cations. Importantly, the obtained compounds exhibit high selectivity and the ability to overcome drug resistance, with compounds **3** and **5** displaying higher activity than cisplatin against selected cancer cell lines. These findings highlight the significant potential of multicomponent reactions in the functional modification of ionophore antibiotics and demonstrate that the Ugi reaction constitutes a powerful strategy for the design of new, structurally diverse and biologically active compounds.

## Experimental Section

### General Procedures

All reagents were purchased from two sources – Merck or Trimen Chemicals S.A., and used without further purification. Deuterated solvent for NMR analysis (CD_2_Cl_2_) was stored over 3Å molecular sieves for several days. Reaction mixtures were stirred using Teflon-coated magnetic stir bars and were monitored by thin layer chromatography (TLC) using aluminum-backed plates (Merck 60 F_254_). TLC plates were visualized by UV-light (254 nm), followed by treatment with phosphomolybdic acid (PMA) (5% in absolute ethanol) and gentle heating. Products of the reactions were purified using the CombiFlash Rf+ Lumen Flash Chromatography System (Teledyne Isco) with integrated ELS and UV detectors. All solvents used in flash chromatography were of HPLC grade (Merck) and were used as received. Solvents were removed using a rotary evaporator.

NMR spectra were recorded on a Bruker AvanceNEO 600 (^1^H NMR at 600 MHz and ^13^C NMR at 151 MHz) magnetic resonance spectrometer. ^1^H NMR spectra are reported in chemical shifts downfield from TMS using the respective residual solvent peak as the internal standard (CD_2_Cl_2_ δ 5.32 ppm). ^1^H NMR spectra are reported as follows: chemical shift (δ, ppm), multiplicity (s = singlet, d = doublet, t = triplet, dd = doublet of doublets, dq = dublets of quartets, qd = quartet of dublets, ddd = doublet of doublet of doublets, dddd = doublet of doublet of doublet of doublets, m = multiplet), coupling constant(s) in Hz, and integration. Significant peaks are reported within the overlapping ∼2.00–0.50 ppm region of the ^1^H NMR spectra. ^13^C NMR spectra are reported in chemical shifts downfield from TMS using the respective residual solvent peak as internal standard (CD_2_Cl_2_ δ 53.84 ppm). Line broadening parameters were 0.5 or 1.0 Hz, while the error of chemical shift value was 0.1 ppm.

Electrospray ionization (ESI) high-resolution mass spectra were recorded on a QTOF (Impact HD, Bruker Daltonics) mass spectrometer in the positive ion detection mode. Samples were prepared in dry acetonitrile with the addition of NaClO_4_. The mass range for ESI experiments was from *m*/*z* = 200 to *m*/*z* = 1200. To analyze the ability of cyclic compounds to form complexes with metal cations, electrospray ionization (ESI) measurements with positive mode detection were performed using PurIonMass Spectrometer Model L. The complexes of compounds **3**–**6** with cations were prepared by mixing acetonitrile solutions of these compounds and respective cation salts in the 1:5 mixture of acetonitrile. The concentration of studied compounds in final solutions used for the ESI measurement was 5 × 10^–5^ mol dm^–3^. The samples were infused directly to the ESI source. The major parameters were set as follows: Source: ESI + 3.5 kV 350 °C, Capillary: 150 V 300 °C Offset: 25 V Span: 0 V, full scan ranging 400–1100 (*m*/*z*).

### Synthesis

#### Isolation of MON

The sodium salt of **MON** was isolated from commercially available veterinary premix – Coxidin®, as previously described.^31^ The obtained sodium salt of **MON** was then extracted with a solution of sulfuric acid (pH = 1), giving **MON** ready for further synthesis.

#### Synthesis of MON Amine (Compound **2**)

To a solution of **MON** (11.0 g, 16.39 mmol, 1.0 equiv) in toluene (120 mL), a solution of 0.1 M Na_2_CO_3_ was added (150 mL) upon stirring. After that, tosyl chloride (9.37 g, 49.18 mmol, 3.0 equiv) was added to the resulting mixture in one portion. The resulting solution was stirred at room temperature for the next 72 h. The reaction mixture was then transferred to a separatory funnel and extracted with 0.1 M Na_2_CO_3_ solution and brine. After that, the organic layer was concentrated under reduced pressure, and directed subsequently to the next step. The excess of sodium azide (3.19 g, 49.09 mmol, 3.0 equiv) was dissolved in 30 mL of DMSO (complete conversion of **MON** to tosylate was assumed). The solution of crude tosylate dissolved in 20 mL of DMSO was then added to the mixture. The resulting solution was stirred at room temperature for the next 72 h. The reaction mixture was then diluted with a large amount of water (over 300 mL) and extracted several times with ethyl acetate. **Caution!** A nonchlorinated solvent must be used for the extraction. The use of chlorinated solvents in the presence of sodium azide may lead to the formation of hazardous species and presents a potential explosion hazard.^38^ Therefore, this precaution must be strictly observed. Purification on silica gel using the CombiFlash system (0 → 40% EtOAc/*n*-hexane) gave the pure azide as a clear oil. The oil was diluted in *n*-pentane and evaporated to dryness three times to form an amorphous solid, with an isolated yield of 62% (7.10 g). Then, under a nitrogen atmosphere, to a solution of **MON** azide (2.0 g, 2.87 mmol, 1.0 equiv) in methanol, the catalytic amount of palladium on carbon (Pd/C) was added upon stirring. A hydrogen-filled balloon was connected to the system. The reaction was continued until the azide was completely consumed (TLC and ESI-MS control), replacing the hydrogen balloon if necessary (typically 72 h). After this time, the reaction mixture was filtered through Celite to remove Pd/C, and concentrated under reduced pressure. Purification on silica gel using the CombiFlash system (0 → 30% MeOH/CHCl_3_) gave the pure product as a clear oil. The oil was diluted in *n*-pentane and evaporated to dryness three times to form an amorphous solid, with an isolated yield of 63% (1.22 g), a single spot by TLC; stains green with PMA. The spectroscopic data were in agreement with those published previously.^30^

#### General Procedure for Preparation of Cyclic Monensin Derivatives Using Ugi Reaction (Compounds **3**–**6**)

In a 100 mL round-bottom flask equipped with a magnetic stir bar, monensin amine **2** (300 mg, 0.45 mmol, 1.0 equiv) was dissolved in methanol (10 mL). Paraformaldehyde (13 mg, 0.45 mmol, 1.0 equiv) was then added, and the mixture was stirred for 30 min to allow efficient imine formation (monitored by ESI-MS). Subsequently, the appropriate isonitrile* (0.45 mmol, 1.0 equiv) was added, and the reaction mixture was diluted with an additional portion of methanol (40 mL). After stirring for 72 h (monitored by TLC and ESI-MS), the reaction mixture was concentrated under reduced pressure. Purification by flash column chromatography on silica gel (CombiFlash, 0→40% EtOAc/*n*-hexane) afforded the desired products as clear oils. The obtained oils were dissolved in *n*-pentane and evaporated to dryness three times to yield amorphous solids.

*Caution: Isonitriles (particularly butyl isonitrile) possess an exceptionally strong and unpleasant odor; therefore, appropriate precautions should be taken when handling these reagents.

#### Cyclic *n*-Butyl Derivative **3**

294 mg, 86% yield. Isolated as a white amorphous solid, a single spot by TLC. Stains green with PMA. Melting point: 232–235 °C. ^1^H NMR (600 MHz, CD_2_Cl_2_) δ 6.61 (dd, *J* = 7.8, 3.9 Hz, 1H), 5.33–5.30 (m, 1H), 5.29 (d, *J* = 1.8 Hz, 1H), 5.11 (d, *J* = 19.0 Hz, 1H), 4.73 (dd, *J* = 13.8, 1.2 Hz, 1H), 4.52 (d, *J* = 8.5 Hz, 1H), 4.38 (s, 1H), 4.33 (ddd, *J* = 10.7, 5.7, 3.2 Hz, 1H), 3.94 (dd, *J* = 11.4, 2.2 Hz, 1H), 3.85 (dd, *J* = 10.4, 3.3 Hz, 1H), 3.82 (dq, *J* = 8.8, 3.0 Hz, 1H), 3.79 (d, *J* = 3.9 Hz, 1H), 3.70 (dd, *J* = 11.1, 4.7 Hz, 1H), 3.62–3.54 (m, 1H), 3.44–3.41 (m, 4H), 2.88 (dddd, *J* = 11.8, 7.6, 5.4, 4.1 Hz, 1H), 2.78 (qd, *J* = 7.1, 3.4 Hz, 1H), 2.49 (d, *J* = 13.8 Hz, 1H), 2.27–1.17 (m, 26H), 1.04 (d, *J* = 7.1 Hz, 3H), 0.98 (t, *J* = 7.4 Hz, 3H), 0.90 (dd, *J* = 6.8, 3.2 Hz, 7H), 0.88 (s, 3H), 0.87–0.84 (m, 6H), 0.80 (d, *J* = 6.5 Hz, 3H) ppm. ^13^C­{^1^H} NMR (151 MHz, CD_2_Cl_2_) δ 179.9, 169.0, 109.7, 99.4, 87.9, 87.7, 85.7, 85.3, 80.1, 77.7, 73.5, 71.8, 68.1, 58.7, 51.4, 39.1, 38.5, 38.2, 37.1, 36.7, 35.8, 35.3, 34.1, 33.0, 32.7, 32.6, 31.8, 29.1, 28.0, 27.6, 20.2, 17.3, 16.7, 15.5, 15.0, 13.8, 10.9, 9.1, 8.4 ppm. HRMS (ESI^+^) *m*/*z*: [M + Na]^+^ Calcd for C_42_H_72_N_2_NaO_10_^+^ 787.5079; Found 787.5098.

#### Cyclic *tert*-Butyl Derivative **4**

158 mg, 46% yield. Isolated as a white amorphous solid, a single spot by TLC. Stains green with PMA. Melting point: 250–253 °C. ^1^H NMR (600 MHz, CD_2_Cl_2_) δ 5.87 (s, 1H), 5.32–5.29 (m, 3H), 4.95 (d, *J* = 18.7 Hz, 1H), 4.73 (d, *J* = 13.1 Hz, 1H), 4.54 (d, *J* = 8.8 Hz, 1H), 4.33 (ddd, *J* = 10.9, 5.6, 3.2 Hz, 1H), 4.16 (d, *J* = 18.9 Hz, 1H), 3.88 (dd, *J* = 11.4, 2.1 Hz, 1H), 3.84 (dd, *J* = 10.4, 3.2 Hz, 1H), 3.79 (dq, *J* = 8.9, 3.0 Hz, 1H), 3.73 (d, *J* = 3.9 Hz, 1H), 3.68 (dd, *J* = 10.8, 4.9 Hz, 1H), 3.44 (t, *J* = 3.3 Hz, 1H), 3.42 (s, 3H), 2.73 (qd, *J* = 7.1, 3.5 Hz, 1H), 2.54 (d, *J* = 13.8 Hz, 1H), 2.24–1.19 (m, 31H), 1.02 (d, *J* = 7.1 Hz, 3H), 0.96 (t, *J* = 7.4 Hz, 3H), 0.90 (dd, *J* = 6.8, 2.6 Hz, 6H), 0.88 (d, *J* = 6.7 Hz, 3H), 0.84 (d, *J* = 7.2 Hz, 3H), 0.79 (d, *J* = 6.5 Hz, 3H) ppm. ^13^C­{^1^H} NMR (151 MHz, CD_2_Cl_2_) δ 179.9, 168.6, 109.5, 99.4, 88.5, 87.8, 85.6, 85.2, 80.0, 77.7, 73.2, 71.6, 68.1, 58.7, 52.2, 51.9, 51.7, 38.4, 38.1, 37.1, 36.6, 36.6, 35.8, 35.3, 35.2, 34.0, 32.9, 32.7, 31.7, 29.0, 28.5, 28.1, 27.7, 17.3, 16.7, 15.5, 14.8, 10.9, 9.3, 8.6 ppm. HRMS (ESI^+^) *m*/*z*: [M + Na]^+^ Calcd for C_42_H_72_N_2_NaO_10_^+^ 787.5079; Found 787.5086.

#### Cyclic Cyclohexyl Derivative **5**

156 mg, 44% yield. Isolated as a white amorphous solid, a single spot by TLC. Stains green with PMA. Melting point: 248–250 °C. ^1^H NMR (600 MHz, CD_2_Cl_2_) δ 6.12 (d, *J* = 8.1 Hz, 1H), 5.33 (d, *J* = 1.7 Hz, 1H), 5.31–5.31 (m, 2H), 5.00 (d, *J* = 18.9 Hz, 1H), 4.72 (dd, *J* = 13.7, 0.8 Hz, 1H), 4.53 (d, *J* = 8.6 Hz, 1H), 4.33 (ddd, *J* = 11.0, 5.7, 3.3 Hz, 1H), 4.29 (d, *J* = 18.9 Hz, 1H), 3.90 (dd, *J* = 11.4, 2.1 Hz, 1H), 3.85 (dd, *J* = 10.4, 3.2 Hz, 1H), 3.80 (dq, *J* = 8.7, 2.9 Hz, 1H), 3.76–3.71 (m, 2H), 3.70 (dd, *J* = 10.9, 4.7 Hz, 1H), 3.43 (t, *J* = 3.3 Hz, 1H), 3.42 (s, 3H), 2.74 (qd, *J* = 7.0, 3.4 Hz, 1H), 2.48 (d, *J* = 13.7 Hz, 1H), 2.24–1.06 (m, 32H), 1.03 (d, *J* = 7.1 Hz, 3H), 0.99 (t, *J* = 7.4 Hz, 3H), 0.90 (d, *J* = 6.7 Hz, 6H), 0.86 (dd, *J* = 14.9, 6.9 Hz, 6H), 0.79 (d, *J* = 6.5 Hz, 3H) ppm. ^13^C­{^1^H} NMR (151 MHz, CD_2_Cl_2_) δ 179.9, 168.1, 109.6, 99.4, 88.5, 87.8, 85.7, 85.5, 80.0, 77.7, 73.3, 71.7, 68.0, 58.7, 51.69, 51.66, 49.0, 38.4, 38.1, 37.1, 36.7, 36.6, 35.7, 35.22, 35.19, 34.1, 33.62, 33.59, 32.9, 32.7, 31.8, 28.5, 28.0, 27.8, 25.9, 25.6, 25.5, 17.3, 16.7, 15.4, 15.0, 10.9, 9.2, 8.5 ppm. HRMS (ESI^+^) *m*/*z*: [M + Na]^+^ Calcd for C_44_H_74_N_2_NaO_10_^+^ 813.5236; Found 813.5243.

#### Cyclic Benzyl Derivative **6**

193 mg, 54% yield. Isolated as a white amorphous solid, a single spot by TLC. UV-active and stains green with PMA. Melting point: 240–243 °C. ^1^H NMR (600 MHz, CD_2_Cl_2_) δ 7.29–7.25 (m, 2H), 7.23–7.17 (m, 3H), 5.33–5.30 (m, 1H), 5.24 (d, *J* = 19.2 Hz, 1H), 4.77 (dd, *J* = 14.6, 7.3 Hz, 2H), 4.51 (d, *J* = 8.4 Hz, 1H), 4.47–4.43 (m, 1H), 4.33 (ddd, *J* = 10.9, 5.8, 3.3 Hz, 1H), 4.11 (dd, *J* = 15.0, 4.8 Hz, 1H), 3.92 (dd, *J* = 11.4, 2.1 Hz, 1H), 3.85 (dd, *J* = 10.4, 3.2 Hz, 1H), 3.81 (dq, *J* = 8.3, 2.7 Hz, 1H), 3.77 (d, *J* = 3.8 Hz, 1H), 3.55 (dd, *J* = 11.1, 4.7 Hz, 1H), 3.45–3.42 (m, 4H), 2.81 (qd, *J* = 7.1, 3.4 Hz, 1H), 2.56–2.53 (m, 1H), 2.22–1.22 (m, 22H), 1.18 (s, 3H), 1.10 (d, *J* = 7.1 Hz, 3H), 0.92 (d, *J* = 6.7 Hz, 3H), 0.88–0.83 (m, 12H), 0.80 (d, *J* = 6.5 Hz, 3H) ppm. ^13^C­{^1^H} NMR (151 MHz, CD_2_Cl_2_) δ 180.0, 169.3, 139.5, 128.8, 127.8, 127.5, 109.7, 99.4, 87.9, 87.8, 85.6, 85.3, 80.1, 77.7, 73.5, 71.8, 68.1, 58.7, 51.4, 43.2, 38.5, 38.1 37.1, 36.73, 36.69, 35.8 35.3 35.2, 34.1 33.0, 32.7, 31.5, 28.9, 27.9, 27.2, 17.2, 16.7, 15.4, 15.2, 10.9, 9.04, 8.5 ppm. HRMS (ESI^+^) *m*/*z*: [M + Na]^+^ Calcd for C_45_H_70_N_2_NaO_10_^+^ 821.4923; Found 821.4948.

### Anticancer Activity Studies

Stock solutions of all tested compounds of a concentration of 10 mM were prepared *ex tempore* for each experiment by dissolving the respective compound in dimethyl sulfoxide (DMSO, Avantor Performance Materials Poland, Gliwice, Poland). The solvent for further dilutions was OR culture medium, which is a mixture of opti-MEM and Roswell Park Memorial Institute (RPMI) medium (1:1) (opti-MEM – GIBCO, Thermo Fisher Scientific, USA, RPMI with l-glutamine – IIET, Wroclaw, Poland), supplemented with 5% fetal bovine serum (FBS, HyClone, Cytiva, USA). The compounds were tested at 8 different concentrations in the range of 100–0.001 μM. As a control, cisplatin and doxorubicin were used in the concentration range of 10–0.01 μM (both drugs from Accord Healthcare, Warsaw, Poland), as well as DMSO solvent in concentrations corresponding to its concentration in the samples in the range of 1–0.001%.

### Cell Lines and Culturing Conditions

Four human cancer cell lines and one murine normal cell line were used to evaluate antiproliferative activity of monensin and its derivatives: human lung carcinoma (A549), human breast adenocarcinoma (MCF7), human colon adenocarcinoma cell lines sensitive and resistant to doxorubicin (LoVo) and (LoVo/DX), respectively, and normal murine embryonic fibroblast cell line (BALB/3T3) clone A31. All cell lines are available in the IIET cell line bank (Wroclaw, Poland). A549 cells were cultured in a mixture of opti-MEM and Roswell Park Memorial Institute (RPMI) medium (1:1) (opti-MEM – GIBCO, Thermo Fisher Scientific, USA, RPMI with l-glutamine – IIET, Wroclaw, Poland), supplemented with 10% fetal bovine serum (FBS, HyClone, Cytiva, USA). MCF7 cells were cultured in a mixture of Eagle’s medium (IIET, Wroclaw, Poland), supplemented with 10% fetal bovine serum, 2 mM l-glutamine, 1% MEM endogenous amino acid solution (Sigma-Aldrich, Germany), and 8 μg/mL insulin from bovine pancreas (Sigma-Aldrich Chemie GmbH, Steinheim, Germany). LoVo and LoVo/DX cells were cultured in OR medium, supplemented with 10% fetal bovine serum (FBS, HyClone, Cytiva, USA) and 1.0 mM of sodium pyruvate (Sigma-Aldrich Chemie GmbH, Steinheim, Germany). Additionally, the medium for LoVo/DX contained 0.1 μg/mL doxorubicin. BALB/3T3 fibroblasts were cultured in Dulbecco’s medium, supplemented with 10% fetal bovine serum (HyClone) and 2 mM l-glutamine. All culture media contained antibiotics: 100 U/mL penicillin and 100 μg/mL streptomycin. All cell lines were cultured in humid atmosphere at 37 °C and 5% CO_2_.

### Cell Viability Assays and SRB

Antiproliferative assays were performed using five cell lines: A549, LoVo, LoVo/DX, MCF7, and BALB/3T3. The cells were plated in 384-well plates (Greiner Bio-One, Kremsmünster, Austria) in the number of: 1500 cells per well for the A549, LoVo/DX and MCF7 cell lines and 2000 cells per well for the LoVo and BALB/3T3 cell lines. After 24 h, solutions of the tested compounds were added. The *in vitro* cytotoxicity of the tested compounds was determined using the sulforhodamine B (SRB) assay.^39^ The cultured cells were exposed to 8 different concentrations of the tested compounds in the range of 0.1–100, 0.01–10, or 0.001–1 μM (depending on the activity of the compound). After 72 h of incubation, the cells were fixed by adding cold 50% (w/v) trichloroacetic acid (TCA) (Avantor Performance Materials, Gliwice, Poland) and were incubated at room temperature for 1 h. Then, the wells were washed with distilled water, and strained with 0.4% (w/v) solution of sulforhodamine B (Sigma-Aldrich, Germany) in 1% (v/v) acetic acid (Avantor Performance Materials, Gliwice, Poland) for 30 min. After incubation time, the plates with stained cells were rinsed with 1% (v/v) acetic acid. The protein-bound dye was solubilized with 10 mM TRIS solution (Avantor Performance Materials, Gliwice, Poland) for 30 min. All steps were performed at room temperature using an EL406 microplate washer (BioTek Instruments Inc., Winooski, Vermont, USA). Absorbance of each solution was read at Synergy H4 Hybrid (BioTek Instruments Inc., Winooski, Vermont, USA) at the 540 nm wavelength. In each experiment, samples containing specific concentrations of the respective compounds were applied in triplicate. The experiments were repeated four or five times. Inhibition of proliferation was determined using the following formula:

$$\%inhibition=\left(\left(\frac{A_{p}-A_{m}}{A_{k}-A_{m}}\right)\times 100\right)-100$$

A_m_ – absorbance of the control medium
A_k_ – absorbance of cell control
A_p_ – absorbance of cells treated with the tested compounds

The experimental results are presented in the form of IC_50_ ± SD (dose causing inhibition of proliferation of 50% of the cancer cell population ± standard deviation). The DMSO was inactive.

## Supplementary Material





## Data Availability

The data underlying this study are available in the published article and its .

## Notes

NMR, and FT–IR, spectra, as well as the details of X-ray crystallography of the reported compounds (PDF)
FAIR data, including the primary NMR FID files, for compounds **3**–**6** (ZIP)
