# Supplementary material for: Access to Cyclic Monensin Derivatives via a Four-Component Ugi Reaction
Source: J Org Chem. 2026 Jul 4;91(28):9933–9. doi: 10.1021/acs.joc.6c01246 (PMC13386529; doi:10.1021/acs.joc.6c01246)

Display Report

Analysis Info

Analysis Name	D:\Data\Gosia\2026\A. Huczynski\26.03.16\MON-UGI-126.03.17_4.d			
Method	Tune_pos_Standard.m			
Sample Name	MON-UGI-1	Instrument	impact HD	1819696.00156
Comment				

Acquisition Parameter

Source Type	ESI	Ion Polarity	Positive	Set Nebulizer	0.3 Bar
Focus	Active	Set Capillary	3600 V	Set Dry Heater	200 °C
Scan Begin	200 m/z	Set End Plate Offset	-500 V	Set Dry Gas	4.0 l/min
Scan End	1200 m/z	Set Charging Voltage	2000 V	Set Divert Valve	Source
		Set Corona	0 nA	Set APCI Heater	0 °C

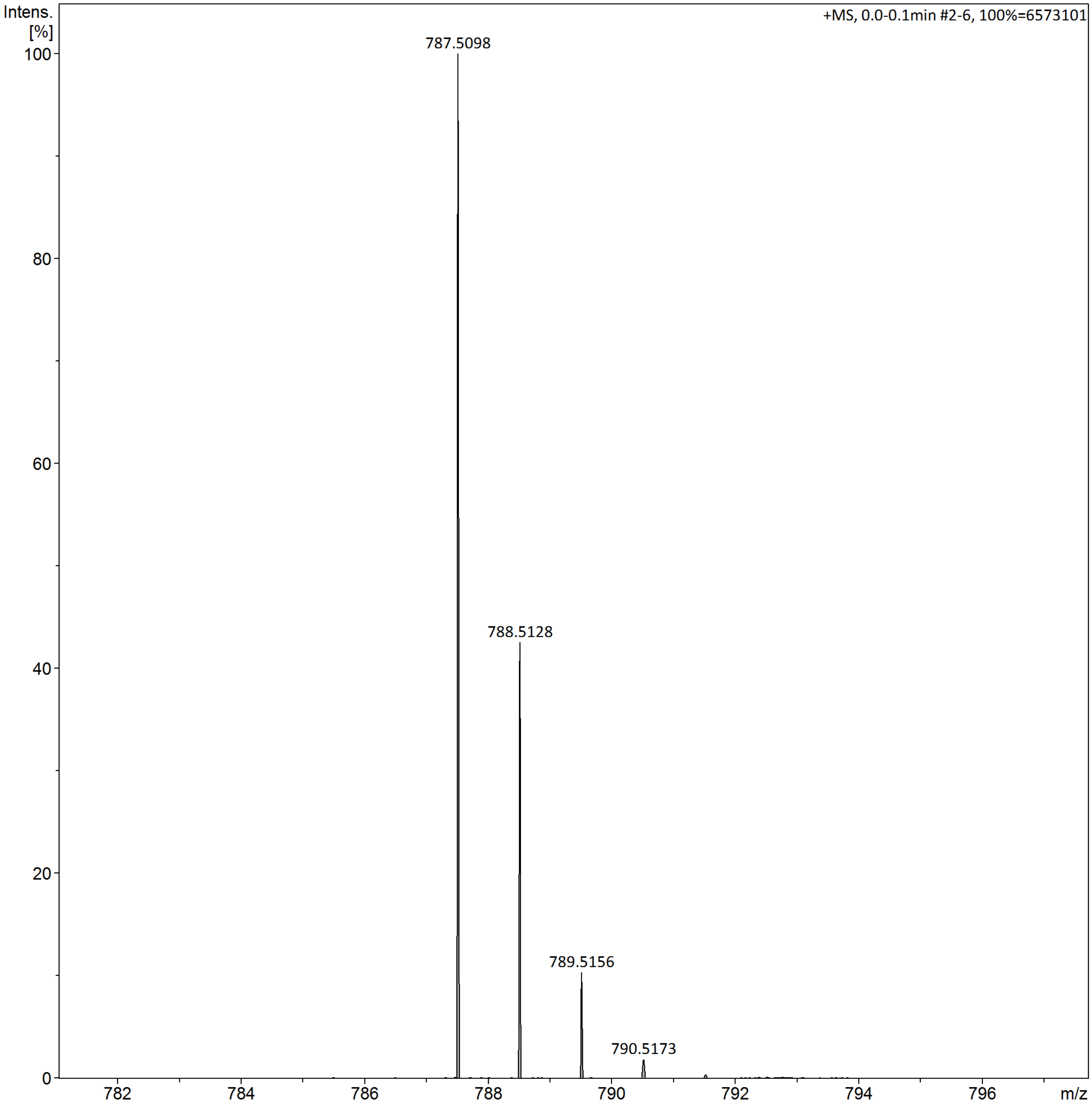

Supplement: Supplementary file 1 [file jo6c01246_si_001.zip › Compouds data/Compound 3/HRMS/MON-UGI-1_zoom1.pdf]
