# Supplementary material for: Access to Cyclic Monensin Derivatives via a Four-Component Ugi Reaction
Source: J Org Chem. 2026 Jul 4;91(28):9933–9. doi: 10.1021/acs.joc.6c01246 (PMC13386529; doi:10.1021/acs.joc.6c01246)

checkCIF/PLATON report

Structure factors have been supplied for datablock(s) MON_UGI1_NaCl_100

THIS REPORT IS FOR GUIDANCE ONLY. IF USED AS PART OF A REVIEW PROCEDURE FOR PUBLICATION, IT SHOULD NOT REPLACE THE EXPERTISE OF AN EXPERIENCED CRYSTALLOGRAPHIC REFEREE.

No syntax errors found. CIF dictionary Interpreting this report

Datablock: MON_UGI1_NaCl_100

Bond precision:	C-C = 0.0058 A		Wavelength=0.71073
Cell:	a=13.7694(11)	b=11.5488(4)	c=14.8152(11)
	alpha=90	beta=117.327(10)	gamma=90
Temperature:	100 K		

	Calculated	Reported
Volume	2093.0(3)	2093.0(3)
Space group	P 21	P 21
Hall group	P 2yb	P 2yb
Moiety formula	C42 H72 N2 O10	C42 H72 N2 O10
Sum formula	C42 H72 N2 O10	C42 H72 N2 O10
Mr	765.02	765.01
Dx,g cm-3	1.214	1.214
Z	2	2
Mu (mm-1)	0.085	0.085
F000	836.0	836.0
F000'	836.41	
h,k,lmax	17,14,18	17,14,18
Nref	9115[4789]	8345
Tmin,Tmax	0.973,0.980	0.985,1.000
Tmin'	0.973	

Correction method= # Reported T Limits: Tmin=0.985 Tmax=1.000
AbsCorr = MULTI-SCAN

```
Data completeness= 1.74/0.92        Theta(max)= 27.000

                                                              wR2(reflections)=
R(reflections)= 0.0505( 6338)                                 0.0921( 8345)

S = 1.001                     Npar= 502
```

```
The following ALERTS were generated. Each ALERT has the format
       test-name_ALERT_alert-type_alert-level.
Click on the hyperlinks for more details of the test.
```

Alert level C

```
STRVA01_ALERT_4_C           Flack test results are ambiguous.
           From the CIF: _refine_ls_abs_structure_Flack    0.600
           From the CIF: _refine_ls_abs_structure_Flack_su    0.500
PLAT029_ALERT_3_C _diffrn_measured_fraction_theta_full value Low .      0.973 Why?
PLAT340_ALERT_3_C Low Bond Precision on  C-C Bonds ..............    0.00579 Ang.
PLAT410_ALERT_2_C Short Intra H...H Contact  H2       ..H37B     .       1.94 Ang.
                                                   x,y,z  =      1_555 Check
PLAT907_ALERT_2_C Flack x > 0.5, Structure Needs to be Inverted? .       0.60 Check
PLAT910_ALERT_3_C Missing FCF Reflection(s) Below Theta(Min)[Deg]=       2.76 Note
               1  0  0,   1  1  0,  -1  0  1,  -1  1  1,   0  0  1,   0  1  1,
               1  0  1,
PLAT911_ALERT_3_C Missing FCF Refl Between Thmin & STh/L=    0.600        100 Report
               7  0  1,   9  0  1,   7  0  2,   7  1  2,   6  1  3,   7  0  3,
               7  1  3,   8  1  3,   9  0  3,  10  0  3,  11  0  3,   6  0  4,
               7  0  4,   8  0  4,   9  0  4,  10  0  4,  11  0  4,   6  2  5,
               7  2  5,  11  0  5,  10  0  6,  11  0  6,   3  0  7,   4  0  7,
               9  0  7,  10  0  7,  10  1  7,   3  0  8,   3  1  8,   4  0  8,
                  (   70 More NOT listed: see .ckf listing file)
```

Alert level G

```
PLAT007_ALERT_5_G Number of Unrefined Donor-H Atoms ..............          2 Report
             H3O     H91
PLAT032_ALERT_4_G Std. Uncertainty on Flack Parameter Value High .      0.500 Report
PLAT398_ALERT_2_G Deviating  C-O-C   Angle From 120 for O6       .      109.0 Degree
PLAT398_ALERT_2_G Deviating  C-O-C   Angle From 120 for O7       .      108.0 Degree
PLAT791_ALERT_4_G Model has Chirality at C2        (Sohncke SpGr)          S Verify
PLAT791_ALERT_4_G Model has Chirality at C3        (Sohncke SpGr)          R Verify
PLAT791_ALERT_4_G Model has Chirality at C4        (Sohncke SpGr)          S Verify
PLAT791_ALERT_4_G Model has Chirality at C5        (Sohncke SpGr)          S Verify
PLAT791_ALERT_4_G Model has Chirality at C6        (Sohncke SpGr)          R Verify
PLAT791_ALERT_4_G Model has Chirality at C7        (Sohncke SpGr)          S Verify
PLAT791_ALERT_4_G Model has Chirality at C9        (Sohncke SpGr)          R Verify
PLAT791_ALERT_4_G Model has Chirality at C12       (Sohncke SpGr)          S Verify
PLAT791_ALERT_4_G Model has Chirality at C13       (Sohncke SpGr)          R Verify
PLAT791_ALERT_4_G Model has Chirality at C16       (Sohncke SpGr)          S Verify
PLAT791_ALERT_4_G Model has Chirality at C17       (Sohncke SpGr)          R Verify
PLAT791_ALERT_4_G Model has Chirality at C18       (Sohncke SpGr)          S Verify
PLAT791_ALERT_4_G Model has Chirality at C20       (Sohncke SpGr)          R Verify
PLAT791_ALERT_4_G Model has Chirality at C21       (Sohncke SpGr)          S Verify
PLAT791_ALERT_4_G Model has Chirality at C22       (Sohncke SpGr)          S Verify
PLAT791_ALERT_4_G Model has Chirality at C24       (Sohncke SpGr)          R Verify
```

```
PLAT791_ALERT_4_G Model has Chirality at C25       (Sohncke SpGr)          R Verify
PLAT899_ALERT_4_G SHELXL2018 is Outdated   and Succeeded by SHELXL     2019/3 Note
PLAT912_ALERT_4_G Missing # of FCF Reflections Above STh/L=  0.600         49 Note
PLAT916_ALERT_2_G Hooft y and Flack x Parameter Values Differ by .       0.30 Check
PLAT941_ALERT_3_G Average HKL Measurement Multiplicity ..........         4.9 Low
PLAT961_ALERT_5_G Dataset Contains no Negative Intensities .......     Please Check
PLAT967_ALERT_5_G Note: Two-Theta Cutoff Value in Embedded .res ..       54.0 Degree
PLAT969_ALERT_5_G The 'Henn et al.' R-Factor-gap value ..........       2.131 Note
              Predicted wR2: Based on SigI**2  4.32 or SHELX Weight  9.20 Note
PLAT978_ALERT_2_G Number C-C Bonds with Positive Residual Density.          1 Info
```

```
 0 ALERT level A = Most likely a serious problem - resolve or explain
 0 ALERT level B = A potentially serious problem, consider carefully
 7 ALERT level C = Check. Ensure it is not caused by an omission or oversight
29 ALERT level G = General information/check it is not something unexpected

 0 ALERT type 1 CIF construction/syntax error, inconsistent or missing data
 6 ALERT type 2 Indicator that the structure model may be wrong or deficient
 5 ALERT type 3 Indicator that the structure quality may be low
21 ALERT type 4 Improvement, methodology, query or suggestion
 4 ALERT type 5 Informative message, check
```

It is advisable to attempt to resolve as many as possible of the alerts in all categories. Often the minor alerts point to easily fixed oversights, errors and omissions in your CIF or refinement strategy, so attention to these fine details can be worthwhile. It is up to the individual to critically assess their own results and, if necessary, seek expert advice.

PLATON version of 23/04/2026; check.def file version of 30/03/2026

---

duplicate check

No duplication found

Datablock MON_UGI1_NaCl_100 - ellipsoid plot

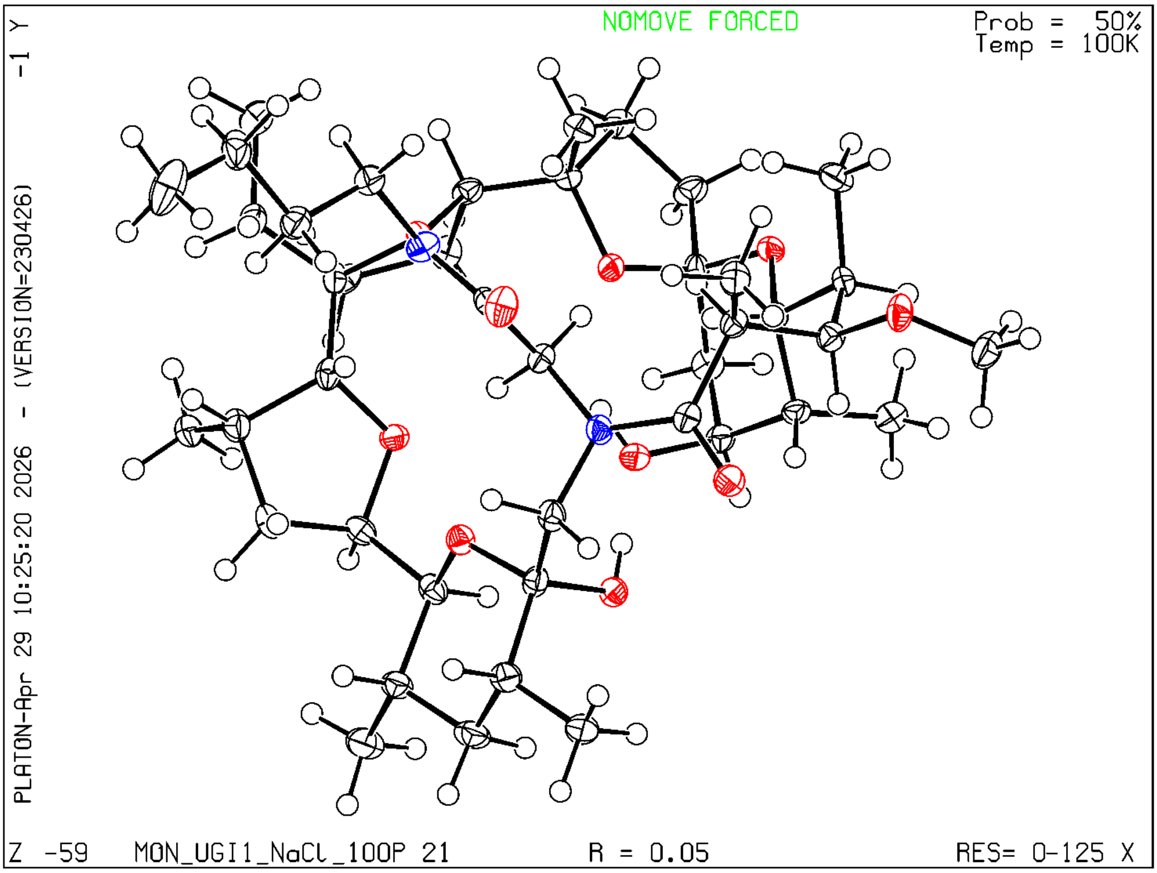

Supplement: Supplementary file 1 [file jo6c01246_si_001.zip › Compouds data/Compound 3/scXRD/checkcif_UGI_I_3.pdf]
