# Supplementary material for: Access to Cyclic Monensin Derivatives via a Four-Component Ugi Reaction
Source: J Org Chem. 2026 Jul 4;91(28):9933–9. doi: 10.1021/acs.joc.6c01246 (PMC13386529; doi:10.1021/acs.joc.6c01246)
Supplement: Supplementary file 1 [file jo6c01246_si_001.zip › Compouds data/Compound 3/scXRD/printcif_MON_UGI_1_NaCl_100.pdf]

Title

Enter author details here

Abstract

Table 1

Experimental details

Crystal data	
Chemical formula	$C_{42}H_{72}N_2O_{10}$
M_r	765.01
Crystal system, space group	Monoclinic, $P2_1$
Temperature (K)	100
a, *b*, *c* (Å)	13.7694 (11), 11.5488 (4), 14.8152 (11)
β (°)	117.327 (10)
V (Å^3)	2093.0 (3)
Z	2
Radiation type	Mo $K\alpha$
μ (mm^{-1})	0.09
Crystal size (mm)	0.33 × 0.31 × 0.24
Data collection	
Diffractometer	Xcalibur, Atlas
Absorption correction	Multi-scan<br>*CrysAlis PRO* 1.171.42.93a (Rigaku Oxford Diffraction, 2023) Empirical absorption correction using spherical harmonics, implemented in SCALE3 ABSPACK scaling algorithm.
T_{min}, T_{max}	0.985, 1.000
No. of measured, independent and observed [$I > 2\sigma(I)$] reflections	22652, 8345, 6338
R_{int}	0.041
(sin $\theta/\lambda)_{max}$ (Å^{-1})	0.639
Refinement	
$R[F^2 > 2\sigma(F^2)]$, $wR(F^2)$, S	0.051, 0.092, 1.00
No. of reflections	8345
No. of parameters	502
No. of restraints	1
H-atom treatment	H atoms treated by a mixture of independent and constrained refinement
$\Delta\rho_{max}$, $\Delta\rho_{min}$ (e Å^{-3})	0.22, −0.27
Absolute structure	Flack x determined using 2144 quotients [(I+)-(I-)]/[(I+)+(I-)] (Parsons, Flack and Wagner, Acta Cryst. B69 (2013) 249-259).
Absolute structure parameter	0.6 (5)

Computer programs: *CrysAlis PRO* 1.171.42.93a (Rigaku Oxford Diffraction, 2023), *SHELXT* 2014/5 (Sheldrick, 2014), *SHELXL2018*/3 (Sheldrick, 2018), Brandenburg & Putz (2006). Diamond 3.0. Crystal and Molecular Structure Visualisation, University of Bonn, Germany.

Table 2

Hydrogen-bond geometry (Å, º)

D—H···*A*	*D*—H	H···*A*	*D*···*A*	*D*—H···*A*
O3—H3*O*···O5	0.84	2.11	2.798 (3)	139
O9—H91···O3	0.84	1.92	2.736 (3)	164
N2—H2*N*···O6	0.93 (4)	2.19 (4)	3.119 (4)	178 (3)

Acknowledgements

Funding information

References

Figure 1

supporting information

Title

Computing details

Data collection: *CrysAlis PRO* 1.171.42.93a (Rigaku Oxford Diffraction, 2023); cell refinement: *CrysAlis PRO* 1.171.42.93a (Rigaku Oxford Diffraction, 2023); data reduction: *CrysAlis PRO* 1.171.42.93a (Rigaku Oxford Diffraction, 2023); program(s) used to solve structure: *SHELXT* 2014/5 (Sheldrick, 2014); program(s) used to refine structure: *SHELXL2018*/3 (Sheldrick, 2018); molecular graphics: Brandenburg & Putz (2006). Diamond 3.0. Crystal and Molecular Structure Visualisation, University of Bonn, Germany.

(MON_UGI1_NaCl_100)

Crystal data

$C_{42}H_{72}N_2O_{10}$
M_r = 765.01
Monoclinic, $P2_1$
a = 13.7694 (11) Å
b = 11.5488 (4) Å
c = 14.8152 (11) Å
β = 117.327 (10)°
V = 2093.0 (3) Å^3
Z = 2
$F(000)$ = 836
D_x = 1.214 Mg m^{-3}
Mo $K\alpha$ radiation, λ = 0.71073 Å
Cell parameters from 6315 reflections
θ = 2.9–26.4°
μ = 0.09 mm^{-1}
T = 100 K
Parallelepiped, colourless
0.33 × 0.31 × 0.24 mm

Data collection

Xcalibur, Atlas diffractometer
Radiation source: fine-focus sealed X-ray tube
Detector resolution: 10.6249 pixels mm^{-1}
ω–scan
Absorption correction: multi-scan *CrysAlis PRO* 1.171.42.93a (Rigaku Oxford Diffraction, 2023) Empirical absorption correction using spherical harmonics, implemented in SCALE3 ABSPACK scaling algorithm.
T_{min} = 0.985, T_{max} = 1.000
22652 measured reflections
8345 independent reflections
6338 reflections with $I > 2\sigma(I)$
R_{int} = 0.041
θ_{max} = 27.0°, θ_{min} = 2.8°
$h = -17 \rightarrow 17$
$k = -14 \rightarrow 14$
$l = -18 \rightarrow 18$

Refinement

Refinement on F^2
Least-squares matrix: full
$R[F^2 > 2\sigma(F^2)]$ = 0.051
$wR(F^2)$ = 0.092
S = 1.00
8345 reflections
502 parameters
1 restraint
Primary atom site location: structure-invariant direct methods
Secondary atom site location: difference Fourier map
Hydrogen site location: mixed
H atoms treated by a mixture of independent and constrained refinement
$w = 1/[\sigma^2(F_o^2) + (0.0367P)^2]$ where $P = (F_o^2 + 2F_c^2)/3$
$(\Delta/\sigma)_{max}$ < 0.001
$\Delta\rho_{max}$ = 0.22 e Å^{-3}
$\Delta\rho_{min}$ = −0.27 e Å^{-3}
Absolute structure: Flack x determined using 2144 quotients [(I+)-(I-)]/[(I+)+(I-)] (Parsons, Flack and Wagner, Acta Cryst. B69 (2013) 249-259).
Absolute structure parameter: 0.6 (5)

Special details

Geometry. All e.s.d.'s (except the e.s.d. in the dihedral angle between two l.s. planes) are estimated using the full covariance matrix. The cell e.s.d.'s are taken into account individually in the estimation of e.s.d.'s in distances, angles and torsion angles; correlations between e.s.d.'s in cell parameters are only used when they are defined by crystal symmetry. An approximate (isotropic) treatment of cell e.s.d.'s is used for estimating e.s.d.'s involving l.s. planes.

Refinement. Refinement of F^2 against ALL reflections. The weighted *R*-factor *wR* and goodness of fit *S* are based on F^2, conventional *R*-factors *R* are based on *F*, with *F* set to zero for negative F^2. The threshold expression of $F^2 > \sigma(F^2)$ is used only for calculating *R*-factors(gt) *etc*. and is not relevant to the choice of reflections for refinement. *R*-factors based on F^2 are statistically about twice as large as those based on *F*, and *R*- factors based on ALL data will be even larger.

Fractional atomic coordinates and isotropic or equivalent isotropic displacement parameters (Å^2)

	x	*y*	*z*	U_{iso}*/U_{eq}
N1	0.3183 (2)	0.6721 (2)	0.6977 (2)	0.0180 (6)
O1	0.38071 (19)	0.84352 (17)	0.77767 (18)	0.0213 (6)
C1	0.3965 (3)	0.7564 (3)	0.7388 (3)	0.0158 (7)
C2	0.5055 (3)	0.7438 (3)	0.7353 (3)	0.0178 (8)
H2	0.511050	0.663049	0.713811	0.021*
C3	0.6003 (3)	0.7641 (3)	0.8436 (3)	0.0181 (8)
H3	0.567602	0.776628	0.890717	0.022*
O2	0.6577 (2)	0.86740 (17)	0.84321 (18)	0.0232 (6)
C4	0.6794 (3)	0.6600 (3)	0.8836 (3)	0.0181 (8)
H4	0.737467	0.679935	0.953337	0.022*
C5	0.6173 (3)	0.5539 (3)	0.8928 (3)	0.0157 (7)
H5	0.548373	0.546111	0.827580	0.019*
C6	0.5860 (3)	0.5605 (3)	0.9799 (3)	0.0169 (7)
H6	0.537278	0.629143	0.967607	0.020*
C7	0.5210 (3)	0.4518 (3)	0.9772 (3)	0.0176 (7)
H7	0.511167	0.451148	1.040040	0.021*
O3	0.41397 (18)	0.45378 (18)	0.89039 (18)	0.0199 (5)
H3O	0.414528	0.412946	0.843709	0.030*
C8	0.5833 (3)	0.3439 (3)	0.9775 (3)	0.0199 (8)
H8A	0.535641	0.275523	0.965872	0.024*
H8B	0.647396	0.335222	1.045029	0.024*
C9	0.6220 (3)	0.3475 (3)	0.8961 (3)	0.0167 (7)
O4	0.68254 (17)	0.45149 (17)	0.90479 (17)	0.0169 (5)
C10	0.6917 (3)	0.2451 (3)	0.8975 (3)	0.0222 (8)
H10A	0.678777	0.178190	0.932451	0.027*
H10B	0.770268	0.265441	0.933227	0.027*
C11	0.6571 (3)	0.2162 (3)	0.7865 (3)	0.0223 (8)
H11A	0.645481	0.131852	0.774576	0.027*
H11B	0.713786	0.241282	0.767097	0.027*
C12	0.5490 (3)	0.2831 (3)	0.7243 (3)	0.0174 (8)
O5	0.52745 (18)	0.34414 (17)	0.79889 (17)	0.0176 (5)
C13	0.4553 (3)	0.1996 (3)	0.6693 (3)	0.0178 (8)
H13	0.473355	0.149132	0.624209	0.021*
C14	0.4225 (3)	0.1229 (3)	0.7340 (3)	0.0203 (8)
H14A	0.423323	0.166552	0.791843	0.024*
H14B	0.471656	0.055173	0.760266	0.024*
C15	0.3070 (3)	0.0859 (3)	0.6597 (3)	0.0187 (8)
H15A	0.262241	0.073336	0.695449	0.022*
H15B	0.308020	0.013637	0.624282	0.022*
C16	0.2615 (3)	0.1876 (3)	0.5840 (3)	0.0178 (7)

O6	0.35567 (18)	0.26293 (18)	0.60683 (17)	0.0175 (5)
C17	0.1771 (3)	0.2624 (3)	0.5971 (3)	0.0178 (8)
H17	0.174289	0.338721	0.564064	0.021*
C18	0.0591 (3)	0.2178 (3)	0.5537 (3)	0.0213 (8)
H18	0.020362	0.229322	0.478423	0.026*
C19	0.0156 (3)	0.3035 (3)	0.6056 (3)	0.0197 (8)
H19A	−0.001190	0.379513	0.570747	0.024*
H19B	−0.050819	0.273307	0.607273	0.024*
C20	0.1106 (3)	0.3127 (3)	0.7120 (3)	0.0183 (8)
H20	0.100766	0.251322	0.754771	0.022*
O7	0.20879 (18)	0.28518 (17)	0.70293 (17)	0.0186 (5)
C21	0.1223 (3)	0.4282 (3)	0.7654 (3)	0.0188 (8)
H21	0.181563	0.422181	0.836936	0.023*
C22	0.0157 (3)	0.4673 (3)	0.7661 (3)	0.0207 (8)
H22	−0.042130	0.473399	0.693953	0.025*
C23	0.0338 (3)	0.5886 (3)	0.8132 (3)	0.0246 (8)
H23A	0.087616	0.583739	0.885934	0.030*
H23B	−0.035865	0.617410	0.809023	0.030*
C24	0.0753 (3)	0.6750 (3)	0.7596 (3)	0.0209 (8)
H24	0.017142	0.683642	0.687858	0.025*
C25	0.1773 (3)	0.6263 (3)	0.7570 (3)	0.0183 (8)
O8	0.15299 (19)	0.51217 (16)	0.71140 (18)	0.0180 (5)
O9	0.26087 (19)	0.62162 (18)	0.85684 (17)	0.0189 (5)
H91	0.310849	0.577796	0.859597	0.028*
C26	0.2091 (3)	0.6969 (3)	0.6870 (3)	0.0190 (8)
H26A	0.154608	0.682171	0.615749	0.023*
H26B	0.205192	0.780225	0.700852	0.023*
C27	0.0937 (3)	0.7936 (3)	0.8106 (3)	0.0250 (9)
H27A	0.111640	0.850188	0.771215	0.037*
H27B	0.154233	0.788766	0.879605	0.037*
H27C	0.027122	0.817903	0.813671	0.037*
C28	−0.0251 (3)	0.3832 (3)	0.8209 (3)	0.0283 (9)
H28A	0.032719	0.370318	0.890208	0.042*
H28B	−0.044588	0.309328	0.784194	0.042*
H28C	−0.089532	0.415870	0.823271	0.042*
C29	0.0439 (3)	0.0927 (3)	0.5798 (3)	0.0240 (8)
H29A	0.079753	0.082911	0.653761	0.036*
H29B	0.076605	0.039420	0.549888	0.036*
H29C	−0.034255	0.075832	0.552426	0.036*
C30	0.2129 (3)	0.1510 (3)	0.4719 (3)	0.0221 (8)
H30A	0.151519	0.097048	0.457221	0.027*
H30B	0.182298	0.220484	0.429066	0.027*
C31	0.2925 (3)	0.0928 (3)	0.4407 (3)	0.0311 (9)
H31A	0.325443	0.025169	0.484020	0.047*
H31B	0.350010	0.147803	0.448479	0.047*
H31C	0.253082	0.068136	0.369620	0.047*
C32	0.5612 (3)	0.3685 (3)	0.6515 (3)	0.0228 (8)
H32A	0.623584	0.419656	0.689737	0.034*
H32B	0.494452	0.414976	0.617712	0.034*
H32C	0.573392	0.325848	0.600473	0.034*
C33	0.6852 (3)	0.5751 (3)	1.0838 (3)	0.0279 (9)
H33A	0.746571	0.530027	1.086048	0.042*

H33B	0.667089	0.547520	1.136844	0.042*
H33C	0.705560	0.657079	1.095246	0.042*
C34	0.7356 (3)	0.6371 (3)	0.8167 (3)	0.0235 (8)
H34A	0.680932	0.612240	0.749029	0.035*
H34B	0.790609	0.576132	0.847546	0.035*
H34C	0.771048	0.708176	0.810696	0.035*
C35	0.7205 (3)	0.9134 (3)	0.9417 (3)	0.0282 (9)
H35A	0.780582	0.860287	0.981522	0.042*
H35B	0.674018	0.923056	0.975198	0.042*
H35C	0.750428	0.988704	0.936602	0.042*
C36	0.5038 (3)	0.8282 (3)	0.6541 (3)	0.0233 (8)
H36A	0.499825	0.907956	0.674860	0.035*
H36B	0.439880	0.812080	0.589012	0.035*
H36C	0.570543	0.818368	0.646881	0.035*
C37	0.3258 (3)	0.5691 (3)	0.6444 (3)	0.0174 (8)
H37A	0.279993	0.507305	0.651598	0.021*
H37B	0.402418	0.541445	0.677081	0.021*
C38	0.2897 (3)	0.5887 (3)	0.5315 (3)	0.0198 (8)
O10	0.2720 (2)	0.68522 (19)	0.49215 (18)	0.0263 (6)
N2	0.2800 (2)	0.4900 (2)	0.4793 (2)	0.0217 (7)
H2N	0.303 (3)	0.421 (3)	0.516 (3)	0.026*
C39	0.2566 (3)	0.4899 (3)	0.3719 (3)	0.0240 (8)
H39A	0.272968	0.411917	0.354621	0.029*
H39B	0.306448	0.545395	0.363293	0.029*
C40	0.1399 (3)	0.5210 (3)	0.2974 (3)	0.0233 (8)
H40A	0.125493	0.602143	0.309121	0.028*
H40B	0.089151	0.470684	0.309968	0.028*
C41	0.1176 (3)	0.5073 (3)	0.1873 (3)	0.0328 (10)
H41A	0.174509	0.550114	0.177779	0.039*
H41B	0.124705	0.424387	0.174447	0.039*
C42	0.0063 (4)	0.5495 (3)	0.1088 (3)	0.0420 (11)
H42A	−0.050975	0.507659	0.117134	0.063*
H42B	−0.001639	0.535414	0.040506	0.063*
H42C	−0.000292	0.632618	0.118058	0.063*

Atomic displacement parameters (Å^2)

	U^{11}	U^{22}	U^{33}	U^{12}	U^{13}	U^{23}
N1	0.0164 (17)	0.0157 (13)	0.0209 (18)	−0.0011 (12)	0.0076 (15)	−0.0025 (12)
O1	0.0218 (14)	0.0159 (12)	0.0271 (15)	−0.0005 (10)	0.0119 (13)	−0.0023 (11)
C1	0.020 (2)	0.0147 (16)	0.0102 (18)	0.0004 (15)	0.0053 (17)	0.0040 (14)
C2	0.020 (2)	0.0147 (17)	0.018 (2)	−0.0008 (15)	0.0087 (18)	−0.0016 (14)
C3	0.022 (2)	0.0154 (16)	0.019 (2)	−0.0036 (15)	0.0104 (19)	−0.0009 (15)
O2	0.0255 (15)	0.0193 (12)	0.0217 (15)	−0.0091 (11)	0.0081 (13)	−0.0039 (10)
C4	0.013 (2)	0.0195 (17)	0.020 (2)	−0.0042 (14)	0.0058 (18)	−0.0039 (14)
C5	0.0130 (19)	0.0172 (16)	0.015 (2)	0.0038 (14)	0.0042 (17)	0.0005 (14)
C6	0.0164 (19)	0.0169 (16)	0.018 (2)	0.0037 (14)	0.0085 (18)	−0.0015 (14)
C7	0.017 (2)	0.0212 (17)	0.0137 (19)	0.0028 (15)	0.0058 (17)	0.0010 (15)
O3	0.0177 (14)	0.0217 (12)	0.0211 (14)	0.0017 (10)	0.0097 (12)	−0.0025 (11)
C8	0.023 (2)	0.0179 (17)	0.018 (2)	0.0030 (15)	0.0087 (18)	0.0047 (15)
C9	0.0127 (19)	0.0175 (16)	0.015 (2)	0.0004 (15)	0.0023 (17)	0.0000 (14)
O4	0.0142 (13)	0.0166 (11)	0.0200 (14)	0.0007 (10)	0.0080 (12)	−0.0022 (10)

C10	0.024 (2)	0.0200 (18)	0.022 (2)	0.0068 (15)	0.010 (2)	−0.0020 (15)
C11	0.020 (2)	0.0249 (18)	0.022 (2)	0.0020 (15)	0.0095 (19)	−0.0017 (15)
C12	0.016 (2)	0.0174 (17)	0.019 (2)	0.0002 (14)	0.0084 (18)	−0.0033 (14)
O5	0.0150 (14)	0.0184 (12)	0.0181 (14)	0.0005 (10)	0.0064 (12)	−0.0037 (10)
C13	0.019 (2)	0.0176 (17)	0.017 (2)	0.0060 (14)	0.0079 (18)	−0.0002 (15)
C14	0.023 (2)	0.0164 (16)	0.019 (2)	0.0010 (15)	0.0072 (18)	0.0015 (14)
C15	0.026 (2)	0.0135 (16)	0.018 (2)	−0.0007 (15)	0.0108 (18)	−0.0025 (14)
C16	0.017 (2)	0.0186 (16)	0.015 (2)	−0.0051 (15)	0.0045 (18)	−0.0023 (15)
O6	0.0136 (13)	0.0195 (11)	0.0170 (14)	−0.0007 (10)	0.0050 (12)	0.0024 (10)
C17	0.019 (2)	0.0183 (17)	0.0126 (19)	−0.0027 (15)	0.0046 (18)	0.0010 (14)
C18	0.019 (2)	0.0250 (17)	0.018 (2)	−0.0033 (15)	0.0062 (19)	−0.0006 (15)
C19	0.014 (2)	0.0207 (17)	0.021 (2)	−0.0025 (14)	0.0043 (18)	0.0033 (15)
C20	0.016 (2)	0.0182 (17)	0.021 (2)	0.0008 (14)	0.0092 (18)	0.0041 (15)
O7	0.0165 (14)	0.0235 (12)	0.0159 (14)	0.0024 (10)	0.0075 (12)	−0.0018 (10)
C21	0.015 (2)	0.0226 (18)	0.017 (2)	0.0006 (15)	0.0057 (18)	0.0034 (15)
C22	0.017 (2)	0.0239 (18)	0.022 (2)	0.0023 (15)	0.0102 (18)	0.0001 (15)
C23	0.018 (2)	0.033 (2)	0.026 (2)	0.0059 (16)	0.0122 (19)	0.0017 (17)
C24	0.017 (2)	0.0270 (18)	0.017 (2)	0.0054 (16)	0.0056 (18)	0.0017 (16)
C25	0.017 (2)	0.0181 (17)	0.016 (2)	0.0006 (15)	0.0037 (18)	−0.0027 (14)
O8	0.0178 (14)	0.0165 (11)	0.0191 (14)	−0.0003 (10)	0.0079 (12)	0.0001 (10)
O9	0.0133 (13)	0.0222 (12)	0.0166 (14)	0.0052 (10)	0.0029 (12)	−0.0010 (10)
C26	0.016 (2)	0.0182 (17)	0.019 (2)	0.0022 (14)	0.0044 (18)	−0.0002 (15)
C27	0.019 (2)	0.0263 (19)	0.028 (2)	0.0049 (16)	0.010 (2)	−0.0041 (16)
C28	0.026 (2)	0.031 (2)	0.035 (3)	0.0002 (17)	0.019 (2)	−0.0002 (17)
C29	0.021 (2)	0.0235 (19)	0.029 (2)	−0.0066 (16)	0.0130 (19)	−0.0063 (17)
C30	0.022 (2)	0.0261 (19)	0.016 (2)	−0.0044 (16)	0.0062 (19)	−0.0023 (15)
C31	0.031 (2)	0.044 (2)	0.020 (2)	−0.0046 (19)	0.012 (2)	−0.0072 (18)
C32	0.020 (2)	0.0259 (19)	0.026 (2)	−0.0021 (16)	0.0135 (19)	−0.0019 (16)
C33	0.026 (2)	0.036 (2)	0.024 (2)	−0.0029 (17)	0.013 (2)	−0.0073 (17)
C34	0.021 (2)	0.0203 (17)	0.036 (2)	−0.0015 (15)	0.018 (2)	−0.0016 (16)
C35	0.034 (3)	0.0248 (19)	0.025 (2)	−0.0066 (17)	0.012 (2)	−0.0087 (16)
C36	0.025 (2)	0.0255 (19)	0.019 (2)	−0.0057 (16)	0.0098 (19)	0.0006 (16)
C37	0.020 (2)	0.0157 (16)	0.016 (2)	−0.0012 (14)	0.0079 (18)	−0.0020 (14)
C38	0.0133 (19)	0.0240 (19)	0.021 (2)	−0.0023 (15)	0.0069 (18)	−0.0004 (16)
O10	0.0307 (17)	0.0222 (13)	0.0206 (15)	−0.0018 (11)	0.0071 (14)	0.0019 (11)
N2	0.0255 (19)	0.0221 (16)	0.0193 (18)	0.0010 (14)	0.0118 (16)	−0.0003 (13)
C39	0.024 (2)	0.0289 (19)	0.022 (2)	−0.0054 (16)	0.013 (2)	−0.0058 (16)
C40	0.025 (2)	0.0240 (19)	0.020 (2)	−0.0018 (16)	0.0099 (19)	−0.0021 (15)
C41	0.032 (3)	0.045 (2)	0.023 (2)	−0.0203 (19)	0.014 (2)	−0.0060 (19)
C42	0.063 (3)	0.027 (2)	0.022 (3)	−0.006 (2)	0.007 (3)	0.0002 (17)

Geometric parameters (Å, º)

N1—C1	1.370 (4)	C21—H21	1.0000
N1—C37	1.458 (4)	C22—C28	1.528 (4)
N1—C26	1.467 (4)	C22—C23	1.533 (5)
O1—C1	1.227 (3)	C22—H22	1.0000
C1—C2	1.532 (4)	C23—C24	1.540 (4)
C2—C36	1.539 (4)	C23—H23A	0.9900
C2—C3	1.553 (5)	C23—H23B	0.9900
C2—H2	1.0000	C24—C27	1.528 (5)
C3—O2	1.433 (4)	C24—C25	1.529 (4)

C3—C4	1.546 (4)	C24—H24	1.0000
C3—H3	1.0000	C25—O9	1.397 (4)
O2—C35	1.414 (4)	C25—O8	1.448 (4)
C4—C34	1.535 (4)	C25—C26	1.533 (4)
C4—C5	1.536 (4)	O9—H91	0.8400
C4—H4	1.0000	C26—H26A	0.9900
C5—O4	1.445 (4)	C26—H26B	0.9900
C5—C6	1.539 (4)	C27—H27A	0.9800
C5—H5	1.0000	C27—H27B	0.9800
C6—C33	1.528 (5)	C27—H27C	0.9800
C6—C7	1.531 (4)	C28—H28A	0.9800
C6—H6	1.0000	C28—H28B	0.9800
C7—O3	1.445 (4)	C28—H28C	0.9800
C7—C8	1.511 (4)	C29—H29A	0.9800
C7—H7	1.0000	C29—H29B	0.9800
O3—H3O	0.8400	C29—H29C	0.9800
C8—C9	1.526 (4)	C30—C31	1.527 (4)
C8—H8A	0.9900	C30—H30A	0.9900
C8—H8B	0.9900	C30—H30B	0.9900
C9—O5	1.432 (4)	C31—H31A	0.9800
C9—O4	1.434 (4)	C31—H31B	0.9800
C9—C10	1.517 (4)	C31—H31C	0.9800
C10—C11	1.525 (5)	C32—H32A	0.9800
C10—H10A	0.9900	C32—H32B	0.9800
C10—H10B	0.9900	C32—H32C	0.9800
C11—C12	1.551 (5)	C33—H33A	0.9800
C11—H11A	0.9900	C33—H33B	0.9800
C11—H11B	0.9900	C33—H33C	0.9800
C12—O5	1.452 (3)	C34—H34A	0.9800
C12—C13	1.514 (5)	C34—H34B	0.9800
C12—C32	1.526 (4)	C34—H34C	0.9800
C13—O6	1.452 (4)	C35—H35A	0.9800
C13—C14	1.518 (4)	C35—H35B	0.9800
C13—H13	1.0000	C35—H35C	0.9800
C14—C15	1.522 (5)	C36—H36A	0.9800
C14—H14A	0.9900	C36—H36B	0.9800
C14—H14B	0.9900	C36—H36C	0.9800
C15—C16	1.545 (4)	C37—C38	1.527 (5)
C15—H15A	0.9900	C37—H37A	0.9900
C15—H15B	0.9900	C37—H37B	0.9900
C16—O6	1.466 (4)	C38—O10	1.229 (4)
C16—C17	1.531 (4)	C38—N2	1.350 (4)
C16—C30	1.537 (5)	N2—C39	1.470 (4)
C17—O7	1.445 (4)	N2—H2N	0.93 (4)
C17—C18	1.536 (5)	C39—C40	1.516 (5)
C17—H17	1.0000	C39—H39A	0.9900
C18—C19	1.534 (4)	C39—H39B	0.9900
C18—C29	1.534 (4)	C40—C41	1.523 (5)
C18—H18	1.0000	C40—H40A	0.9900
C19—C20	1.522 (5)	C40—H40B	0.9900
C19—H19A	0.9900	C41—C42	1.517 (5)
C19—H19B	0.9900	C41—H41A	0.9900

C20—O7	1.455 (4)	C41—H41B	0.9900
C20—C21	1.521 (4)	C42—H42A	0.9800
C20—H20	1.0000	C42—H42B	0.9800
C21—O8	1.439 (3)	C42—H42C	0.9800
C21—C22	1.541 (4)		

C1—N1—C37	125.8 (3)	C20—C21—H21	109.3
C1—N1—C26	117.8 (2)	C22—C21—H21	109.3
C37—N1—C26	115.2 (3)	C28—C22—C23	111.2 (3)
O1—C1—N1	121.3 (3)	C28—C22—C21	113.6 (3)
O1—C1—C2	118.7 (3)	C23—C22—C21	108.3 (3)
N1—C1—C2	120.0 (3)	C28—C22—H22	107.8
C1—C2—C36	107.6 (3)	C23—C22—H22	107.8
C1—C2—C3	108.8 (3)	C21—C22—H22	107.8
C36—C2—C3	114.6 (3)	C22—C23—C24	112.3 (3)
C1—C2—H2	108.6	C22—C23—H23A	109.1
C36—C2—H2	108.6	C24—C23—H23A	109.1
C3—C2—H2	108.6	C22—C23—H23B	109.1
O2—C3—C4	110.9 (2)	C24—C23—H23B	109.1
O2—C3—C2	109.0 (2)	H23A—C23—H23B	107.9
C4—C3—C2	112.9 (3)	C27—C24—C25	113.4 (3)
O2—C3—H3	108.0	C27—C24—C23	110.1 (3)
C4—C3—H3	108.0	C25—C24—C23	109.7 (3)
C2—C3—H3	108.0	C27—C24—H24	107.9
C35—O2—C3	112.7 (2)	C25—C24—H24	107.9
C34—C4—C5	112.6 (2)	C23—C24—H24	107.9
C34—C4—C3	111.2 (3)	O9—C25—O8	111.1 (3)
C5—C4—C3	109.4 (2)	O9—C25—C24	107.9 (3)
C34—C4—H4	107.8	O8—C25—C24	109.2 (3)
C5—C4—H4	107.8	O9—C25—C26	112.6 (3)
C3—C4—H4	107.8	O8—C25—C26	104.2 (2)
O4—C5—C4	108.9 (2)	C24—C25—C26	111.8 (3)
O4—C5—C6	109.5 (2)	C21—O8—C25	114.7 (2)
C4—C5—C6	114.6 (3)	C25—O9—H91	109.5
O4—C5—H5	107.9	N1—C26—C25	114.9 (3)
C4—C5—H5	107.9	N1—C26—H26A	108.5
C6—C5—H5	107.9	C25—C26—H26A	108.5
C33—C6—C7	111.0 (3)	N1—C26—H26B	108.5
C33—C6—C5	112.7 (3)	C25—C26—H26B	108.5
C7—C6—C5	108.9 (3)	H26A—C26—H26B	107.5
C33—C6—H6	108.1	C24—C27—H27A	109.5
C7—C6—H6	108.1	C24—C27—H27B	109.5
C5—C6—H6	108.1	H27A—C27—H27B	109.5
O3—C7—C8	111.1 (3)	C24—C27—H27C	109.5
O3—C7—C6	110.8 (3)	H27A—C27—H27C	109.5
C8—C7—C6	110.6 (3)	H27B—C27—H27C	109.5
O3—C7—H7	108.1	C22—C28—H28A	109.5
C8—C7—H7	108.1	C22—C28—H28B	109.5
C6—C7—H7	108.1	H28A—C28—H28B	109.5
C7—O3—H3O	109.5	C22—C28—H28C	109.5
C7—C8—C9	112.3 (3)	H28A—C28—H28C	109.5
C7—C8—H8A	109.1	H28B—C28—H28C	109.5

C9—C8—H8A	109.1	C18—C29—H29A	109.5
C7—C8—H8B	109.1	C18—C29—H29B	109.5
C9—C8—H8B	109.1	H29A—C29—H29B	109.5
H8A—C8—H8B	107.9	C18—C29—H29C	109.5
O5—C9—O4	109.6 (2)	H29A—C29—H29C	109.5
O5—C9—C10	105.7 (3)	H29B—C29—H29C	109.5
O4—C9—C10	108.3 (2)	C31—C30—C16	115.6 (3)
O5—C9—C8	107.9 (3)	C31—C30—H30A	108.4
O4—C9—C8	110.6 (2)	C16—C30—H30A	108.4
C10—C9—C8	114.6 (3)	C31—C30—H30B	108.4
C9—O4—C5	111.8 (2)	C16—C30—H30B	108.4
C9—C10—C11	105.9 (3)	H30A—C30—H30B	107.5
C9—C10—H10A	110.6	C30—C31—H31A	109.5
C11—C10—H10A	110.6	C30—C31—H31B	109.5
C9—C10—H10B	110.6	H31A—C31—H31B	109.5
C11—C10—H10B	110.6	C30—C31—H31C	109.5
H10A—C10—H10B	108.7	H31A—C31—H31C	109.5
C10—C11—C12	105.7 (3)	H31B—C31—H31C	109.5
C10—C11—H11A	110.6	C12—C32—H32A	109.5
C12—C11—H11A	110.6	C12—C32—H32B	109.5
C10—C11—H11B	110.6	H32A—C32—H32B	109.5
C12—C11—H11B	110.6	C12—C32—H32C	109.5
H11A—C11—H11B	108.7	H32A—C32—H32C	109.5
O5—C12—C13	107.3 (2)	H32B—C32—H32C	109.5
O5—C12—C32	110.6 (2)	C6—C33—H33A	109.5
C13—C12—C32	112.0 (3)	C6—C33—H33B	109.5
O5—C12—C11	105.4 (3)	H33A—C33—H33B	109.5
C13—C12—C11	110.5 (3)	C6—C33—H33C	109.5
C32—C12—C11	110.8 (3)	H33A—C33—H33C	109.5
C9—O5—C12	111.7 (2)	H33B—C33—H33C	109.5
O6—C13—C12	110.1 (2)	C4—C34—H34A	109.5
O6—C13—C14	103.2 (2)	C4—C34—H34B	109.5
C12—C13—C14	117.3 (3)	H34A—C34—H34B	109.5
O6—C13—H13	108.6	C4—C34—H34C	109.5
C12—C13—H13	108.6	H34A—C34—H34C	109.5
C14—C13—H13	108.6	H34B—C34—H34C	109.5
C13—C14—C15	102.9 (3)	O2—C35—H35A	109.5
C13—C14—H14A	111.2	O2—C35—H35B	109.5
C15—C14—H14A	111.2	H35A—C35—H35B	109.5
C13—C14—H14B	111.2	O2—C35—H35C	109.5
C15—C14—H14B	111.2	H35A—C35—H35C	109.5
H14A—C14—H14B	109.1	H35B—C35—H35C	109.5
C14—C15—C16	104.6 (2)	C2—C36—H36A	109.5
C14—C15—H15A	110.8	C2—C36—H36B	109.5
C16—C15—H15A	110.8	H36A—C36—H36B	109.5
C14—C15—H15B	110.8	C2—C36—H36C	109.5
C16—C15—H15B	110.8	H36A—C36—H36C	109.5
H15A—C15—H15B	108.9	H36B—C36—H36C	109.5
O6—C16—C17	106.2 (2)	N1—C37—C38	113.8 (3)
O6—C16—C30	108.2 (2)	N1—C37—H37A	108.8
C17—C16—C30	108.3 (3)	C38—C37—H37A	108.8
O6—C16—C15	105.2 (3)	N1—C37—H37B	108.8

C17—C16—C15	114.5 (2)	C38—C37—H37B	108.8
C30—C16—C15	113.9 (3)	H37A—C37—H37B	107.7
C13—O6—C16	109.0 (2)	O10—C38—N2	123.2 (3)
O7—C17—C16	111.8 (3)	O10—C38—C37	123.2 (3)
O7—C17—C18	104.5 (2)	N2—C38—C37	113.5 (3)
C16—C17—C18	118.4 (3)	C38—N2—C39	122.4 (3)
O7—C17—H17	107.2	C38—N2—H2N	118 (2)
C16—C17—H17	107.2	C39—N2—H2N	119 (2)
C18—C17—H17	107.2	N2—C39—C40	114.9 (3)
C19—C18—C29	110.6 (3)	N2—C39—H39A	108.5
C19—C18—C17	98.9 (3)	C40—C39—H39A	108.5
C29—C18—C17	116.8 (3)	N2—C39—H39B	108.5
C19—C18—H18	110.0	C40—C39—H39B	108.5
C29—C18—H18	110.0	H39A—C39—H39B	107.5
C17—C18—H18	110.0	C39—C40—C41	112.4 (3)
C20—C19—C18	102.5 (3)	C39—C40—H40A	109.1
C20—C19—H19A	111.3	C41—C40—H40A	109.1
C18—C19—H19A	111.3	C39—C40—H40B	109.1
C20—C19—H19B	111.3	C41—C40—H40B	109.1
C18—C19—H19B	111.3	H40A—C40—H40B	107.9
H19A—C19—H19B	109.2	C42—C41—C40	115.1 (3)
O7—C20—C21	111.0 (3)	C42—C41—H41A	108.5
O7—C20—C19	106.4 (3)	C40—C41—H41A	108.5
C21—C20—C19	115.8 (3)	C42—C41—H41B	108.5
O7—C20—H20	107.8	C40—C41—H41B	108.5
C21—C20—H20	107.8	H41A—C41—H41B	107.5
C19—C20—H20	107.8	C41—C42—H42A	109.5
C17—O7—C20	108.0 (2)	C41—C42—H42B	109.5
O8—C21—C20	106.8 (2)	H42A—C42—H42B	109.5
O8—C21—C22	109.3 (2)	C41—C42—H42C	109.5
C20—C21—C22	112.8 (3)	H42A—C42—H42C	109.5
O8—C21—H21	109.3	H42B—C42—H42C	109.5

C37—N1—C1—O1	176.0 (3)	C14—C15—C16—C30	128.3 (3)
C26—N1—C1—O1	9.3 (5)	C12—C13—O6—C16	−160.1 (2)
C37—N1—C1—C2	−3.0 (5)	C14—C13—O6—C16	−34.1 (3)
C26—N1—C1—C2	−169.7 (3)	C17—C16—O6—C13	136.9 (3)
O1—C1—C2—C36	−73.3 (4)	C30—C16—O6—C13	−107.0 (3)
N1—C1—C2—C36	105.8 (3)	C15—C16—O6—C13	15.1 (3)
O1—C1—C2—C3	51.4 (4)	O6—C16—C17—O7	−73.3 (3)
N1—C1—C2—C3	−129.6 (3)	C30—C16—C17—O7	170.7 (2)
C1—C2—C3—O2	−114.2 (3)	C15—C16—C17—O7	42.4 (4)
C36—C2—C3—O2	6.3 (3)	O6—C16—C17—C18	165.2 (3)
C1—C2—C3—C4	122.1 (3)	C30—C16—C17—C18	49.2 (4)
C36—C2—C3—C4	−117.4 (3)	C15—C16—C17—C18	−79.2 (4)
C4—C3—O2—C35	−74.5 (3)	O7—C17—C18—C19	42.4 (3)
C2—C3—O2—C35	160.6 (3)	C16—C17—C18—C19	167.6 (3)
O2—C3—C4—C34	−60.4 (4)	O7—C17—C18—C29	−76.2 (3)
C2—C3—C4—C34	62.2 (3)	C16—C17—C18—C29	49.0 (4)
O2—C3—C4—C5	174.6 (2)	C29—C18—C19—C20	82.4 (3)
C2—C3—C4—C5	−62.8 (3)	C17—C18—C19—C20	−40.7 (3)
C34—C4—C5—O4	41.4 (4)	C18—C19—C20—O7	26.0 (3)

C3—C4—C5—O4	165.6 (3)	C18—C19—C20—C21	149.8 (3)
C34—C4—C5—C6	164.5 (3)	C16—C17—O7—C20	−157.0 (2)
C3—C4—C5—C6	−71.4 (4)	C18—C17—O7—C20	−27.7 (3)
O4—C5—C6—C33	64.1 (3)	C21—C20—O7—C17	−125.8 (3)
C4—C5—C6—C33	−58.7 (4)	C19—C20—O7—C17	1.0 (3)
O4—C5—C6—C7	−59.5 (3)	O7—C20—C21—O8	53.5 (3)
C4—C5—C6—C7	177.8 (3)	C19—C20—C21—O8	−67.9 (3)
C33—C6—C7—O3	165.0 (2)	O7—C20—C21—C22	173.6 (3)
C5—C6—C7—O3	−70.4 (3)	C19—C20—C21—C22	52.1 (4)
C33—C6—C7—C8	−71.3 (3)	O8—C21—C22—C28	179.4 (3)
C5—C6—C7—C8	53.3 (4)	C20—C21—C22—C28	60.7 (4)
O3—C7—C8—C9	72.7 (3)	O8—C21—C22—C23	−56.5 (4)
C6—C7—C8—C9	−50.8 (4)	C20—C21—C22—C23	−175.2 (3)
C7—C8—C9—O5	−66.6 (3)	C28—C22—C23—C24	179.8 (3)
C7—C8—C9—O4	53.3 (4)	C21—C22—C23—C24	54.3 (4)
C7—C8—C9—C10	176.0 (3)	C22—C23—C24—C27	−179.1 (3)
O5—C9—O4—C5	58.8 (3)	C22—C23—C24—C25	−53.7 (4)
C10—C9—O4—C5	173.6 (3)	C27—C24—C25—O9	56.7 (4)
C8—C9—O4—C5	−60.1 (3)	C23—C24—C25—O9	−66.8 (3)
C4—C5—O4—C9	−169.9 (3)	C27—C24—C25—O8	177.6 (3)
C6—C5—O4—C9	64.0 (3)	C23—C24—C25—O8	54.1 (4)
O5—C9—C10—C11	22.7 (3)	C27—C24—C25—C26	−67.6 (4)
O4—C9—C10—C11	−94.7 (3)	C23—C24—C25—C26	168.9 (3)
C8—C9—C10—C11	141.4 (3)	C20—C21—O8—C25	−174.9 (3)
C9—C10—C11—C12	−13.7 (3)	C22—C21—O8—C25	62.8 (4)
C10—C11—C12—O5	−0.1 (3)	O9—C25—O8—C21	57.5 (3)
C10—C11—C12—C13	−115.7 (3)	C24—C25—O8—C21	−61.4 (3)
C10—C11—C12—C32	119.5 (3)	C26—C25—O8—C21	179.0 (3)
O4—C9—O5—C12	92.4 (3)	C1—N1—C26—C25	−111.3 (3)
C10—C9—O5—C12	−24.1 (3)	C37—N1—C26—C25	80.6 (4)
C8—C9—O5—C12	−147.1 (2)	O9—C25—C26—N1	45.4 (4)
C13—C12—O5—C9	133.0 (3)	O8—C25—C26—N1	−75.1 (3)
C32—C12—O5—C9	−104.6 (3)	C24—C25—C26—N1	167.0 (3)
C11—C12—O5—C9	15.2 (3)	O6—C16—C30—C31	55.4 (4)
O5—C12—C13—O6	68.8 (3)	C17—C16—C30—C31	170.1 (3)
C32—C12—C13—O6	−52.8 (3)	C15—C16—C30—C31	−61.2 (4)
C11—C12—C13—O6	−176.8 (2)	C1—N1—C37—C38	−85.4 (4)
O5—C12—C13—C14	−48.8 (3)	C26—N1—C37—C38	81.6 (3)
C32—C12—C13—C14	−170.3 (3)	N1—C37—C38—O10	10.6 (5)
C11—C12—C13—C14	65.6 (3)	N1—C37—C38—N2	−170.0 (3)
O6—C13—C14—C15	38.9 (3)	O10—C38—N2—C39	5.4 (5)
C12—C13—C14—C15	160.2 (3)	C37—C38—N2—C39	−174.0 (3)
C13—C14—C15—C16	−29.7 (3)	C38—N2—C39—C40	−73.7 (4)
C14—C15—C16—O6	9.9 (3)	N2—C39—C40—C41	−174.1 (3)
C14—C15—C16—C17	−106.3 (3)	C39—C40—C41—C42	−173.6 (3)

Hydrogen-bond geometry (Å, º)

D—H···*A*	*D*—H	H···*A*	*D*···*A*	*D*—H···*A*
O3—H3*O*···O5	0.84	2.11	2.798 (3)	139
O9—H91···O3	0.84	1.92	2.736 (3)	164
N2—H2*N*···O6	0.93 (4)	2.19 (4)	3.119 (4)	178 (3)
