# Supplementary material for: Access to Cyclic Monensin Derivatives via a Four-Component Ugi Reaction
Source: J Org Chem. 2026 Jul 4;91(28):9933–9. doi: 10.1021/acs.joc.6c01246 (PMC13386529; doi:10.1021/acs.joc.6c01246)
Supplement: Supplementary file 1 [file jo6c01246_si_001.zip › Compouds data/Compound 4/scXRD/printcif_MON_UGI8_100.pdf]

Title

Enter author details here

Abstract

Table 1

Experimental details

Crystal data	
Chemical formula	$C_{42}H_{72}N_2O_{10}$
M_r	765.01
Crystal system, space group	Monoclinic, $P2_1$
Temperature (K)	100
a, b, c (Å)	13.5682 (18), 11.2722 (7), 15.2350 (14)
β (°)	109.469 (13)
V (Å^3)	2196.9 (4)
Z	2
Radiation type	Mo $K\alpha$
μ (mm^{-1})	0.08
Crystal size (mm)	0.29 × 0.25 × 0.15
Data collection	
Diffractometer	Xcalibur, Atlas
Absorption correction	Multi-scan<br>*CrysAlis PRO* 1.171.42.93a (Rigaku Oxford Diffraction, 2023) Empirical absorption correction using spherical harmonics, implemented in SCALE3 ABSPACK scaling algorithm.
T_{min}, T_{max}	0.988, 1.000
No. of measured, independent and observed [$I > 2\sigma(I)$] reflections	20268, 9716, 4595
R_{int}	0.071
(sin $\theta/\lambda)_{max}$ (Å^{-1})	0.650
Refinement	
$R[F^2 > 2\sigma(F^2)]$, $wR(F^2)$, S	0.078, 0.124, 1.00
No. of reflections	9716
No. of parameters	504
No. of restraints	2
H-atom treatment	H atoms treated by a mixture of independent and constrained refinement
$\Delta\rho_{max}$, $\Delta\rho_{min}$ (e Å^{-3})	0.25, −0.29
Absolute structure	Flack x determined using 1266 quotients [(I+)-(I-)]/[(I+)+(I-)] (Parsons, Flack and Wagner, Acta Cryst. B69 (2013) 249-259).
Absolute structure parameter	−0.2 (10)

Computer programs: *CrysAlis PRO* 1.171.42.93a (Rigaku Oxford Diffraction, 2023), *SHELXT* 2014/5 (Sheldrick, 2014), *SHELXL2018*/3 (Sheldrick, 2018), Brandenburg & Putz (2006). Diamond 3.0. Crystal and Molecular Structure Visualisation, University of Bonn, Germany.

Table 2

Hydrogen-bond geometry (Å, º)

D—H···*A*	*D*—H	H···*A*	*D*···*A*	*D*—H···*A*
O3—H3*O*···O5	0.84	2.11	2.796 (6)	139
O9—H9*O*···O3	0.84	1.93	2.746 (5)	164
N2—H2*N*···O6	0.86	2.45	3.300 (6)	170

Acknowledgements

Funding information

References

Figure 1

supporting information

Title

Computing details

Data collection: *CrysAlis PRO* 1.171.42.93a (Rigaku Oxford Diffraction, 2023); cell refinement: *CrysAlis PRO* 1.171.42.93a (Rigaku Oxford Diffraction, 2023); data reduction: *CrysAlis PRO* 1.171.42.93a (Rigaku Oxford Diffraction, 2023); program(s) used to solve structure: *SHELXT* 2014/5 (Sheldrick, 2014); program(s) used to refine structure: *SHELXL2018*/3 (Sheldrick, 2018); molecular graphics: Brandenburg & Putz (2006). Diamond 3.0. Crystal and Molecular Structure Visualisation, University of Bonn, Germany.

(monugi8_100)

Crystal data

$C_{42}H_{72}N_2O_{10}$
M_r = 765.01
Monoclinic, $P2_1$
a = 13.5682 (18) Å
b = 11.2722 (7) Å
c = 15.2350 (14) Å
β = 109.469 (13)°
V = 2196.9 (4) Å^3
Z = 2
F(000) = 836
D_x = 1.156 Mg m^{-3}
Mo $K\alpha$ radiation, λ = 0.71073 Å
Cell parameters from 2893 reflections
θ = 2.8–22.6°
μ = 0.08 mm^{-1}
T = 100 K
Parallelepiped, colourless
0.29 × 0.25 × 0.15 mm

Data collection

Xcalibur, Atlas diffractometer
Radiation source: fine-focus sealed X-ray tube
Detector resolution: 10.6249 pixels mm^{-1}
ω–scan
Absorption correction: multi-scan *CrysAlis PRO* 1.171.42.93a (Rigaku Oxford Diffraction, 2023) Empirical absorption correction using spherical harmonics, implemented in SCALE3 ABSPACK scaling algorithm.
T_{min} = 0.988, T_{max} = 1.000
20268 measured reflections
9716 independent reflections
4595 reflections with $I > 2\sigma(I)$
R_{int} = 0.071
θ_{max} = 27.5°, θ_{min} = 2.5°
h = −15→17
k = −14→14
l = −19→19

Refinement

Refinement on F^2
Least-squares matrix: full
$R[F^2 > 2\sigma(F^2)]$ = 0.078
$wR(F^2)$ = 0.124
S = 1.00
9716 reflections
504 parameters
2 restraints
Primary atom site location: structure-invariant direct methods
Secondary atom site location: difference Fourier map
Hydrogen site location: mixed
H atoms treated by a mixture of independent and constrained refinement
$w = 1/[\sigma^2(F_o^2) + (0.0127P)^2]$ where $P = (F_o^2 + 2F_c^2)/3$
$(\Delta/\sigma)_{max}$ < 0.001
$\Delta\rho_{max}$ = 0.25 e Å^{-3}
$\Delta\rho_{min}$ = −0.29 e Å^{-3}
Absolute structure: Flack x determined using 1266 quotients [(I+)-(I-)]/[(I+)+(I-)] (Parsons, Flack and Wagner, Acta Cryst. B69 (2013) 249-259).
Absolute structure parameter: −0.2 (10)

Special details

Geometry. All e.s.d.'s (except the e.s.d. in the dihedral angle between two l.s. planes) are estimated using the full covariance matrix. The cell e.s.d.'s are taken into account individually in the estimation of e.s.d.'s in distances, angles and torsion angles; correlations between e.s.d.'s in cell parameters are only used when they are defined by crystal symmetry. An approximate (isotropic) treatment of cell e.s.d.'s is used for estimating e.s.d.'s involving l.s. planes.

Refinement. Refinement of F^2 against ALL reflections. The weighted *R*-factor *wR* and goodness of fit *S* are based on F^2, conventional *R*-factors *R* are based on *F*, with *F* set to zero for negative F^2. The threshold expression of $F^2 > \sigma(F^2)$ is used only for calculating *R*-factors(gt) *etc*. and is not relevant to the choice of reflections for refinement. *R*-factors based on F^2 are statistically about twice as large as those based on *F*, and *R*- factors based on ALL data will be even larger.

Fractional atomic coordinates and isotropic or equivalent isotropic displacement parameters (Å^2)

	x	*y*	*z*	U_{iso}*/U_{eq}
N1	0.6060 (4)	0.6869 (4)	0.2854 (3)	0.0280 (13)
O1	0.5934 (3)	0.8526 (3)	0.3654 (3)	0.0320 (11)
C1	0.6451 (5)	0.7667 (5)	0.3572 (4)	0.0271 (15)
C2	0.7564 (5)	0.7506 (5)	0.4255 (4)	0.0280 (16)
H2	0.780230	0.668199	0.418832	0.034*
C3	0.7532 (5)	0.7661 (4)	0.5272 (4)	0.0286 (16)
H3	0.679385	0.781763	0.523404	0.034*
O2	0.8165 (3)	0.8688 (3)	0.5687 (3)	0.0349 (12)
C4	0.7927 (5)	0.6551 (5)	0.5878 (4)	0.0255 (15)
H4	0.787027	0.672742	0.650273	0.031*
C5	0.7180 (5)	0.5517 (5)	0.5469 (4)	0.0256 (15)
H5	0.712362	0.544085	0.479972	0.031*
C6	0.6070 (5)	0.5655 (5)	0.5496 (4)	0.0292 (16)
H6	0.575402	0.638108	0.513367	0.035*
C7	0.5417 (5)	0.4585 (5)	0.5027 (4)	0.0299 (16)
H7	0.473713	0.461727	0.514833	0.036*
O3	0.5196 (4)	0.4635 (3)	0.4026 (3)	0.0338 (12)
H3O	0.561841	0.419763	0.388068	0.051*
C8	0.5961 (5)	0.3437 (5)	0.5429 (4)	0.0274 (15)
H8A	0.593971	0.333360	0.606744	0.033*
H8B	0.557937	0.276456	0.504672	0.033*
C9	0.7084 (5)	0.3413 (5)	0.5458 (4)	0.0282 (16)
O4	0.7650 (3)	0.4432 (3)	0.5931 (3)	0.0264 (10)
C10	0.7690 (5)	0.2302 (5)	0.5890 (4)	0.0318 (17)
H10A	0.721277	0.161514	0.580461	0.038*
H10B	0.805763	0.241914	0.656409	0.038*
C11	0.8476 (5)	0.2099 (5)	0.5378 (4)	0.0393 (18)
H11A	0.853573	0.124441	0.525530	0.047*
H11B	0.917548	0.240511	0.574806	0.047*
C12	0.8018 (5)	0.2804 (5)	0.4445 (4)	0.0313 (16)
O5	0.7098 (3)	0.3416 (3)	0.4518 (3)	0.0283 (10)
C13	0.7646 (5)	0.1959 (5)	0.3628 (4)	0.0326 (17)
H13	0.823026	0.141253	0.363838	0.039*
C14	0.6681 (5)	0.1232 (5)	0.3554 (4)	0.0318 (17)
H14A	0.615310	0.171565	0.371125	0.038*
H14B	0.685987	0.053249	0.397003	0.038*
C15	0.6283 (6)	0.0857 (5)	0.2525 (4)	0.0363 (18)
H15A	0.551400	0.075618	0.229804	0.044*
H15B	0.661286	0.010597	0.243244	0.044*
C16	0.6613 (5)	0.1903 (5)	0.2018 (4)	0.0306 (16)

O6	0.7323 (3)	0.2631 (3)	0.2757 (3)	0.0300 (11)
C17	0.5719 (5)	0.2719 (5)	0.1486 (4)	0.0321 (16)
H17	0.604003	0.348710	0.139423	0.039*
C18	0.4990 (6)	0.2319 (5)	0.0523 (4)	0.0358 (17)
H18	0.533333	0.245414	0.004349	0.043*
C19	0.4101 (5)	0.3218 (5)	0.0395 (4)	0.0349 (18)
H19A	0.344442	0.294357	−0.007859	0.042*
H19B	0.428802	0.400695	0.021281	0.042*
C20	0.4000 (5)	0.3249 (5)	0.1372 (4)	0.0303 (16)
H20	0.351681	0.259777	0.141261	0.036*
O7	0.5038 (4)	0.2979 (3)	0.2013 (3)	0.0317 (11)
C21	0.3586 (5)	0.4420 (5)	0.1626 (4)	0.0311 (16)
H21	0.348390	0.432770	0.224271	0.037*
C22	0.2562 (5)	0.4808 (5)	0.0911 (5)	0.0332 (17)
H22	0.267781	0.488901	0.029925	0.040*
C23	0.2263 (5)	0.6039 (5)	0.1182 (4)	0.0363 (18)
H23A	0.163040	0.632579	0.068543	0.044*
H23B	0.209226	0.596903	0.176269	0.044*
C24	0.3143 (5)	0.6943 (5)	0.1326 (4)	0.0337 (17)
H24	0.326539	0.704973	0.071886	0.040*
C25	0.4151 (5)	0.6454 (5)	0.2035 (4)	0.0289 (16)
O8	0.4385 (3)	0.5301 (3)	0.1709 (3)	0.0296 (11)
O9	0.3990 (4)	0.6353 (3)	0.2889 (3)	0.0316 (11)
H9O	0.445538	0.591925	0.324570	0.047*
C26	0.5108 (5)	0.7202 (5)	0.2093 (4)	0.0296 (16)
H26A	0.524484	0.713270	0.149630	0.035*
H26B	0.494754	0.804403	0.217159	0.035*
C27	0.2817 (5)	0.8153 (5)	0.1617 (4)	0.0422 (19)
H27A	0.275787	0.808712	0.223827	0.063*
H27B	0.214178	0.839169	0.116939	0.063*
H27C	0.334643	0.875018	0.162653	0.063*
C28	0.1673 (5)	0.3912 (5)	0.0785 (4)	0.0443 (19)
H28A	0.184000	0.317221	0.052718	0.067*
H28B	0.102043	0.424028	0.035684	0.067*
H28C	0.159073	0.375273	0.138866	0.067*
C29	0.4550 (5)	0.1049 (5)	0.0439 (4)	0.042 (2)
H29A	0.512561	0.047797	0.057011	0.063*
H29B	0.406424	0.092157	−0.019377	0.063*
H29C	0.418023	0.094006	0.088647	0.063*
C30	0.7197 (5)	0.1499 (5)	0.1355 (4)	0.0360 (18)
H30A	0.673570	0.095329	0.088684	0.043*
H30B	0.731942	0.220343	0.101699	0.043*
C31	0.8249 (5)	0.0876 (5)	0.1820 (5)	0.044 (2)
H31A	0.858099	0.070891	0.135201	0.066*
H31B	0.813261	0.012942	0.210086	0.066*
H31C	0.870562	0.139064	0.230445	0.066*
C32	0.8812 (5)	0.3693 (5)	0.4340 (4)	0.0349 (17)
H32A	0.848128	0.420659	0.380345	0.052*
H32B	0.939863	0.326855	0.424427	0.052*
H32C	0.906997	0.417777	0.490480	0.052*
C33	0.6066 (5)	0.5809 (5)	0.6500 (4)	0.0391 (18)
H33A	0.664176	0.535072	0.692617	0.059*

H33B	0.540025	0.552366	0.654001	0.059*
H33C	0.615419	0.664971	0.667153	0.059*
C34	0.9075 (5)	0.6283 (5)	0.6036 (4)	0.0368 (18)
H34A	0.917414	0.615246	0.543545	0.055*
H34B	0.928311	0.556918	0.642110	0.055*
H34C	0.950475	0.695493	0.635445	0.055*
C35	0.7872 (6)	0.9150 (5)	0.6435 (4)	0.044 (2)
H35A	0.711283	0.926247	0.622806	0.065*
H35B	0.822088	0.991331	0.663364	0.065*
H35C	0.808143	0.859190	0.695805	0.065*
C36	0.8281 (5)	0.8384 (5)	0.3993 (4)	0.0346 (17)
H36A	0.824580	0.824257	0.334833	0.052*
H36B	0.900142	0.827569	0.440944	0.052*
H36C	0.805371	0.919674	0.405222	0.052*
C37	0.6619 (5)	0.5845 (5)	0.2682 (4)	0.0309 (17)
H37A	0.610365	0.524852	0.232695	0.037*
H37B	0.702720	0.548387	0.328608	0.037*
C38	0.7362 (6)	0.6134 (5)	0.2141 (4)	0.0345 (18)
O10	0.7417 (4)	0.7135 (3)	0.1844 (3)	0.0389 (12)
N2	0.7939 (5)	0.5205 (4)	0.2047 (4)	0.0352 (14)
H2N	0.774 (4)	0.451 (3)	0.216 (4)	0.042*
C39	0.8811 (6)	0.5248 (6)	0.1667 (5)	0.0394 (18)
C40	0.9312 (6)	0.4007 (5)	0.1802 (5)	0.052 (2)
H40A	0.956422	0.380792	0.246652	0.079*
H40B	0.990045	0.400197	0.156479	0.079*
H40C	0.879135	0.342109	0.146229	0.079*
C41	0.8379 (6)	0.5550 (6)	0.0621 (4)	0.057 (2)
H41A	0.787166	0.494435	0.029342	0.086*
H41B	0.895505	0.557082	0.036798	0.086*
H41C	0.803598	0.632704	0.053653	0.086*
C42	0.9619 (6)	0.6164 (6)	0.2196 (5)	0.056 (2)
H42A	0.929202	0.695020	0.212106	0.083*
H42B	1.019882	0.617605	0.194799	0.083*
H42C	0.988348	0.595519	0.285768	0.083*

Atomic displacement parameters (Å^2)

	U^{11}	U^{22}	U^{33}	U^{12}	U^{13}	U^{23}
N1	0.031 (4)	0.029 (3)	0.024 (3)	0.004 (2)	0.010 (3)	−0.003 (2)
O1	0.037 (3)	0.024 (2)	0.037 (3)	0.004 (2)	0.014 (2)	−0.001 (2)
C1	0.043 (5)	0.021 (3)	0.024 (4)	−0.005 (3)	0.019 (3)	0.002 (3)
C2	0.040 (5)	0.019 (3)	0.025 (4)	0.003 (3)	0.011 (3)	−0.002 (3)
C3	0.036 (5)	0.023 (3)	0.027 (4)	−0.004 (3)	0.010 (3)	−0.003 (3)
O2	0.054 (4)	0.025 (2)	0.026 (3)	−0.007 (2)	0.014 (3)	−0.0042 (19)
C4	0.035 (5)	0.029 (3)	0.014 (3)	0.000 (3)	0.010 (3)	0.002 (3)
C5	0.037 (5)	0.024 (3)	0.021 (3)	0.001 (3)	0.016 (3)	0.000 (3)
C6	0.036 (5)	0.024 (3)	0.028 (4)	0.001 (3)	0.010 (3)	0.009 (3)
C7	0.035 (5)	0.030 (3)	0.026 (4)	0.001 (3)	0.011 (3)	0.002 (3)
O3	0.042 (3)	0.034 (3)	0.024 (3)	0.005 (2)	0.009 (2)	0.002 (2)
C8	0.033 (4)	0.027 (3)	0.025 (4)	−0.001 (3)	0.012 (3)	0.001 (3)
C9	0.050 (5)	0.014 (3)	0.023 (4)	0.000 (3)	0.015 (4)	0.001 (3)
O4	0.038 (3)	0.021 (2)	0.022 (2)	0.004 (2)	0.012 (2)	−0.0016 (18)

C10	0.042 (5)	0.026 (3)	0.028 (4)	0.006 (3)	0.013 (4)	0.005 (3)
C11	0.051 (5)	0.031 (4)	0.036 (4)	0.003 (3)	0.015 (4)	−0.001 (3)
C12	0.039 (5)	0.035 (4)	0.018 (4)	0.001 (3)	0.007 (3)	−0.004 (3)
O5	0.034 (3)	0.032 (2)	0.023 (2)	0.004 (2)	0.013 (2)	0.0018 (19)
C13	0.040 (5)	0.038 (4)	0.020 (4)	0.000 (3)	0.011 (3)	0.000 (3)
C14	0.042 (5)	0.028 (3)	0.025 (4)	0.003 (3)	0.011 (4)	−0.002 (3)
C15	0.055 (5)	0.021 (3)	0.038 (4)	−0.001 (3)	0.022 (4)	−0.003 (3)
C16	0.042 (5)	0.028 (3)	0.022 (4)	−0.003 (3)	0.011 (4)	−0.008 (3)
O6	0.042 (3)	0.031 (2)	0.018 (2)	−0.007 (2)	0.011 (2)	−0.0009 (19)
C17	0.037 (5)	0.029 (3)	0.032 (4)	0.003 (3)	0.014 (4)	−0.002 (3)
C18	0.047 (5)	0.045 (4)	0.020 (4)	−0.005 (3)	0.018 (4)	−0.003 (3)
C19	0.046 (5)	0.033 (4)	0.027 (4)	−0.006 (3)	0.015 (4)	−0.007 (3)
C20	0.032 (5)	0.028 (3)	0.032 (4)	0.000 (3)	0.011 (3)	−0.002 (3)
O7	0.045 (3)	0.032 (2)	0.021 (2)	0.003 (2)	0.016 (2)	0.0001 (18)
C21	0.028 (4)	0.042 (4)	0.025 (4)	−0.001 (3)	0.010 (3)	−0.004 (3)
C22	0.025 (5)	0.047 (4)	0.028 (4)	0.000 (3)	0.009 (4)	−0.002 (3)
C23	0.034 (5)	0.049 (4)	0.025 (4)	0.011 (3)	0.008 (4)	0.005 (3)
C24	0.030 (5)	0.040 (4)	0.027 (4)	0.003 (3)	0.005 (3)	0.003 (3)
C25	0.038 (5)	0.030 (3)	0.020 (4)	0.005 (3)	0.011 (3)	0.001 (3)
O8	0.038 (3)	0.031 (2)	0.023 (2)	−0.003 (2)	0.014 (2)	−0.0032 (19)
O9	0.041 (3)	0.034 (3)	0.022 (3)	0.005 (2)	0.014 (2)	0.0004 (19)
C26	0.040 (5)	0.032 (3)	0.019 (4)	0.003 (3)	0.013 (3)	−0.002 (3)
C27	0.048 (5)	0.048 (4)	0.031 (4)	0.009 (4)	0.014 (4)	0.009 (3)
C28	0.044 (5)	0.062 (5)	0.030 (4)	−0.001 (4)	0.016 (4)	−0.003 (3)
C29	0.061 (6)	0.041 (4)	0.026 (4)	−0.004 (4)	0.016 (4)	−0.011 (3)
C30	0.039 (5)	0.047 (4)	0.025 (4)	−0.001 (3)	0.015 (4)	−0.006 (3)
C31	0.048 (6)	0.047 (4)	0.041 (5)	0.005 (4)	0.019 (4)	−0.006 (3)
C32	0.043 (5)	0.031 (4)	0.032 (4)	−0.001 (3)	0.015 (4)	−0.002 (3)
C33	0.051 (5)	0.037 (4)	0.034 (4)	0.002 (3)	0.021 (4)	−0.005 (3)
C34	0.043 (5)	0.031 (4)	0.029 (4)	−0.010 (3)	0.002 (4)	0.001 (3)
C35	0.071 (6)	0.031 (4)	0.029 (4)	−0.008 (3)	0.016 (4)	−0.010 (3)
C36	0.040 (5)	0.034 (4)	0.031 (4)	0.003 (3)	0.013 (4)	0.001 (3)
C37	0.036 (5)	0.034 (4)	0.023 (4)	0.001 (3)	0.010 (3)	0.006 (3)
C38	0.054 (6)	0.038 (4)	0.015 (4)	−0.002 (4)	0.017 (4)	−0.003 (3)
O10	0.049 (3)	0.036 (3)	0.036 (3)	0.002 (2)	0.020 (3)	0.007 (2)
N2	0.046 (4)	0.026 (3)	0.039 (4)	0.003 (3)	0.022 (3)	−0.001 (3)
C39	0.041 (5)	0.047 (4)	0.037 (4)	−0.002 (4)	0.022 (4)	−0.005 (3)
C40	0.046 (6)	0.053 (4)	0.065 (6)	0.005 (4)	0.028 (5)	−0.004 (4)
C41	0.054 (6)	0.090 (6)	0.032 (5)	0.014 (5)	0.021 (4)	0.007 (4)
C42	0.045 (6)	0.056 (5)	0.068 (6)	−0.004 (4)	0.022 (5)	−0.003 (4)

Geometric parameters (Å, º)

N1—C1	1.378 (7)	C21—H21	1.0000
N1—C37	1.453 (7)	C22—C28	1.535 (8)
N1—C26	1.468 (7)	C22—C23	1.540 (7)
O1—C1	1.227 (6)	C22—H22	1.0000
C1—C2	1.533 (8)	C23—C24	1.529 (8)
C2—C36	1.531 (8)	C23—H23A	0.9900
C2—C3	1.573 (7)	C23—H23B	0.9900
C2—H2	1.0000	C24—C25	1.536 (8)
C3—O2	1.454 (6)	C24—C27	1.544 (8)

C3—C4	1.541 (7)	C24—H24	1.0000
C3—H3	1.0000	C25—O9	1.393 (6)
O2—C35	1.425 (6)	C25—O8	1.463 (6)
C4—C34	1.524 (8)	C25—C26	1.526 (8)
C4—C5	1.535 (7)	O9—H9O	0.8400
C4—H4	1.0000	C26—H26A	0.9900
C5—O4	1.448 (6)	C26—H26B	0.9900
C5—C6	1.529 (8)	C27—H27A	0.9800
C5—H5	1.0000	C27—H27B	0.9800
C6—C7	1.526 (7)	C27—H27C	0.9800
C6—C33	1.541 (7)	C28—H28A	0.9800
C6—H6	1.0000	C28—H28B	0.9800
C7—O3	1.454 (6)	C28—H28C	0.9800
C7—C8	1.514 (7)	C29—H29A	0.9800
C7—H7	1.0000	C29—H29B	0.9800
O3—H3O	0.8400	C29—H29C	0.9800
C8—C9	1.511 (8)	C30—C31	1.537 (8)
C8—H8A	0.9900	C30—H30A	0.9900
C8—H8B	0.9900	C30—H30B	0.9900
C9—O4	1.434 (6)	C31—H31A	0.9800
C9—O5	1.438 (6)	C31—H31B	0.9800
C9—C10	1.522 (7)	C31—H31C	0.9800
C10—C11	1.534 (8)	C32—H32A	0.9800
C10—H10A	0.9900	C32—H32B	0.9800
C10—H10B	0.9900	C32—H32C	0.9800
C11—C12	1.565 (8)	C33—H33A	0.9800
C11—H11A	0.9900	C33—H33B	0.9800
C11—H11B	0.9900	C33—H33C	0.9800
C12—O5	1.463 (7)	C34—H34A	0.9800
C12—C13	1.514 (7)	C34—H34B	0.9800
C12—C32	1.518 (8)	C34—H34C	0.9800
C13—O6	1.463 (6)	C35—H35A	0.9800
C13—C14	1.517 (8)	C35—H35B	0.9800
C13—H13	1.0000	C35—H35C	0.9800
C14—C15	1.538 (7)	C36—H36A	0.9800
C14—H14A	0.9900	C36—H36B	0.9800
C14—H14B	0.9900	C36—H36C	0.9800
C15—C16	1.554 (8)	C37—C38	1.535 (8)
C15—H15A	0.9900	C37—H37A	0.9900
C15—H15B	0.9900	C37—H37B	0.9900
C16—O6	1.465 (7)	C38—O10	1.227 (6)
C16—C17	1.523 (8)	C38—N2	1.344 (7)
C16—C30	1.546 (8)	N2—C39	1.481 (8)
C17—O7	1.443 (7)	N2—H2N	0.86
C17—C18	1.540 (8)	C39—C42	1.525 (8)
C17—H17	1.0000	C39—C40	1.539 (8)
C18—C19	1.538 (8)	C39—C41	1.541 (8)
C18—C29	1.539 (8)	C40—H40A	0.9800
C18—H18	1.0000	C40—H40B	0.9800
C19—C20	1.540 (8)	C40—H40C	0.9800
C19—H19A	0.9900	C41—H41A	0.9800
C19—H19B	0.9900	C41—H41B	0.9800

C20—O7	1.453 (7)	C41—H41C	0.9800
C20—C21	1.534 (8)	C42—H42A	0.9800
C20—H20	1.0000	C42—H42B	0.9800
C21—O8	1.445 (7)	C42—H42C	0.9800
C21—C22	1.516 (8)		

C1—N1—C37	125.2 (5)	C22—C21—H21	108.9
C1—N1—C26	117.7 (5)	C20—C21—H21	108.9
C37—N1—C26	115.8 (5)	C21—C22—C28	113.2 (5)
O1—C1—N1	120.9 (6)	C21—C22—C23	109.1 (5)
O1—C1—C2	119.4 (5)	C28—C22—C23	111.1 (5)
N1—C1—C2	119.6 (5)	C21—C22—H22	107.7
C36—C2—C1	108.3 (5)	C28—C22—H22	107.7
C36—C2—C3	114.4 (5)	C23—C22—H22	107.7
C1—C2—C3	108.1 (5)	C24—C23—C22	112.1 (5)
C36—C2—H2	108.6	C24—C23—H23A	109.2
C1—C2—H2	108.6	C22—C23—H23A	109.2
C3—C2—H2	108.6	C24—C23—H23B	109.2
O2—C3—C4	110.4 (4)	C22—C23—H23B	109.2
O2—C3—C2	108.1 (4)	H23A—C23—H23B	107.9
C4—C3—C2	112.5 (5)	C23—C24—C25	109.4 (5)
O2—C3—H3	108.6	C23—C24—C27	110.3 (5)
C4—C3—H3	108.6	C25—C24—C27	112.8 (5)
C2—C3—H3	108.6	C23—C24—H24	108.1
C35—O2—C3	111.0 (4)	C25—C24—H24	108.1
C34—C4—C5	114.5 (5)	C27—C24—H24	108.1
C34—C4—C3	112.9 (5)	O9—C25—O8	111.4 (4)
C5—C4—C3	108.8 (5)	O9—C25—C26	112.7 (5)
C34—C4—H4	106.7	O8—C25—C26	103.5 (5)
C5—C4—H4	106.7	O9—C25—C24	107.8 (5)
C3—C4—H4	106.7	O8—C25—C24	108.8 (5)
O4—C5—C6	110.5 (4)	C26—C25—C24	112.5 (5)
O4—C5—C4	108.7 (5)	C21—O8—C25	113.5 (5)
C6—C5—C4	115.8 (5)	C25—O9—H9O	109.5
O4—C5—H5	107.2	N1—C26—C25	114.3 (5)
C6—C5—H5	107.2	N1—C26—H26A	108.7
C4—C5—H5	107.2	C25—C26—H26A	108.7
C7—C6—C5	109.3 (5)	N1—C26—H26B	108.7
C7—C6—C33	111.1 (5)	C25—C26—H26B	108.7
C5—C6—C33	111.6 (5)	H26A—C26—H26B	107.6
C7—C6—H6	108.3	C24—C27—H27A	109.5
C5—C6—H6	108.3	C24—C27—H27B	109.5
C33—C6—H6	108.3	H27A—C27—H27B	109.5
O3—C7—C8	111.1 (4)	C24—C27—H27C	109.5
O3—C7—C6	110.2 (4)	H27A—C27—H27C	109.5
C8—C7—C6	111.0 (5)	H27B—C27—H27C	109.5
O3—C7—H7	108.2	C22—C28—H28A	109.5
C8—C7—H7	108.2	C22—C28—H28B	109.5
C6—C7—H7	108.2	H28A—C28—H28B	109.5
C7—O3—H3O	109.5	C22—C28—H28C	109.5
C9—C8—C7	112.2 (5)	H28A—C28—H28C	109.5
C9—C8—H8A	109.2	H28B—C28—H28C	109.5

C7—C8—H8A	109.2
C9—C8—H8B	109.2
C7—C8—H8B	109.2
H8A—C8—H8B	107.9
O4—C9—O5	108.4 (4)
O4—C9—C8	111.5 (4)
O5—C9—C8	108.5 (5)
O4—C9—C10	108.7 (5)
O5—C9—C10	104.4 (4)
C8—C9—C10	114.9 (5)
C9—O4—C5	110.9 (4)
C9—C10—C11	105.7 (5)
C9—C10—H10A	110.6
C11—C10—H10A	110.6
C9—C10—H10B	110.6
C11—C10—H10B	110.6
H10A—C10—H10B	108.7
C10—C11—C12	104.6 (5)
C10—C11—H11A	110.8
C12—C11—H11A	110.8
C10—C11—H11B	110.8
C12—C11—H11B	110.8
H11A—C11—H11B	108.9
O5—C12—C13	107.4 (5)
O5—C12—C32	110.6 (5)
C13—C12—C32	112.5 (5)
O5—C12—C11	105.1 (5)
C13—C12—C11	110.5 (5)
C32—C12—C11	110.5 (5)
C9—O5—C12	111.6 (4)
O6—C13—C12	109.7 (4)
O6—C13—C14	102.9 (5)
C12—C13—C14	116.3 (5)
O6—C13—H13	109.2
C12—C13—H13	109.2
C14—C13—H13	109.2
C13—C14—C15	103.0 (5)
C13—C14—H14A	111.2
C15—C14—H14A	111.2
C13—C14—H14B	111.2
C15—C14—H14B	111.2
H14A—C14—H14B	109.1
C14—C15—C16	103.7 (5)
C14—C15—H15A	111.0
C16—C15—H15A	111.0
C14—C15—H15B	111.0
C16—C15—H15B	111.0
H15A—C15—H15B	109.0
O6—C16—C17	105.9 (4)
O6—C16—C30	108.5 (5)
C17—C16—C30	108.7 (5)
O6—C16—C15	105.5 (4)
C18—C29—H29A	109.5
C18—C29—H29B	109.5
H29A—C29—H29B	109.5
C18—C29—H29C	109.5
H29A—C29—H29C	109.5
H29B—C29—H29C	109.5
C31—C30—C16	115.6 (5)
C31—C30—H30A	108.4
C16—C30—H30A	108.4
C31—C30—H30B	108.4
C16—C30—H30B	108.4
H30A—C30—H30B	107.4
C30—C31—H31A	109.5
C30—C31—H31B	109.5
H31A—C31—H31B	109.5
C30—C31—H31C	109.5
H31A—C31—H31C	109.5
H31B—C31—H31C	109.5
C12—C32—H32A	109.5
C12—C32—H32B	109.5
H32A—C32—H32B	109.5
C12—C32—H32C	109.5
H32A—C32—H32C	109.5
H32B—C32—H32C	109.5
C6—C33—H33A	109.5
C6—C33—H33B	109.5
H33A—C33—H33B	109.5
C6—C33—H33C	109.5
H33A—C33—H33C	109.5
H33B—C33—H33C	109.5
C4—C34—H34A	109.5
C4—C34—H34B	109.5
H34A—C34—H34B	109.5
C4—C34—H34C	109.5
H34A—C34—H34C	109.5
H34B—C34—H34C	109.5
O2—C35—H35A	109.5
O2—C35—H35B	109.5
H35A—C35—H35B	109.5
O2—C35—H35C	109.5
H35A—C35—H35C	109.5
H35B—C35—H35C	109.5
C2—C36—H36A	109.5
C2—C36—H36B	109.5
H36A—C36—H36B	109.5
C2—C36—H36C	109.5
H36A—C36—H36C	109.5
H36B—C36—H36C	109.5
N1—C37—C38	113.8 (5)
N1—C37—H37A	108.8
C38—C37—H37A	108.8
N1—C37—H37B	108.8

C17—C16—C15	114.3 (6)
C30—C16—C15	113.4 (5)
C13—O6—C16	109.1 (4)
O7—C17—C16	111.8 (5)
O7—C17—C18	105.2 (5)
C16—C17—C18	118.4 (5)
O7—C17—H17	106.9
C16—C17—H17	106.9
C18—C17—H17	106.9
C19—C18—C29	109.6 (6)
C19—C18—C17	98.9 (5)
C29—C18—C17	117.4 (5)
C19—C18—H18	110.1
C29—C18—H18	110.1
C17—C18—H18	110.1
C18—C19—C20	102.2 (5)
C18—C19—H19A	111.3
C20—C19—H19A	111.3
C18—C19—H19B	111.3
C20—C19—H19B	111.3
H19A—C19—H19B	109.2
O7—C20—C21	111.4 (5)
O7—C20—C19	105.5 (5)
C21—C20—C19	114.9 (5)
O7—C20—H20	108.3
C21—C20—H20	108.3
C19—C20—H20	108.3
C17—O7—C20	109.0 (4)
O8—C21—C22	110.3 (5)
O8—C21—C20	106.6 (5)
C22—C21—C20	113.1 (5)
O8—C21—H21	108.9
C38—C37—H37B	108.8
H37A—C37—H37B	107.7
O10—C38—N2	124.6 (6)
O10—C38—C37	121.7 (6)
N2—C38—C37	113.7 (5)
C38—N2—C39	125.8 (5)
C38—N2—H2N	117 (4)
C39—N2—H2N	117 (4)
N2—C39—C42	110.0 (5)
N2—C39—C40	107.0 (5)
C42—C39—C40	109.7 (6)
N2—C39—C41	109.5 (6)
C42—C39—C41	110.8 (6)
C40—C39—C41	109.8 (5)
C39—C40—H40A	109.5
C39—C40—H40B	109.5
H40A—C40—H40B	109.5
C39—C40—H40C	109.5
H40A—C40—H40C	109.5
H40B—C40—H40C	109.5
C39—C41—H41A	109.5
C39—C41—H41B	109.5
H41A—C41—H41B	109.5
C39—C41—H41C	109.5
H41A—C41—H41C	109.5
H41B—C41—H41C	109.5
C39—C42—H42A	109.5
C39—C42—H42B	109.5
H42A—C42—H42B	109.5
C39—C42—H42C	109.5
H42A—C42—H42C	109.5
H42B—C42—H42C	109.5

C37—N1—C1—O1	178.3 (5)
C26—N1—C1—O1	12.1 (8)
C37—N1—C1—C2	0.7 (8)
C26—N1—C1—C2	−165.5 (5)
O1—C1—C2—C36	−75.8 (7)
N1—C1—C2—C36	101.9 (6)
O1—C1—C2—C3	48.8 (7)
N1—C1—C2—C3	−133.6 (5)
C36—C2—C3—O2	3.8 (7)
C1—C2—C3—O2	−117.0 (5)
C36—C2—C3—C4	−118.3 (6)
C1—C2—C3—C4	120.9 (5)
C4—C3—O2—C35	−78.6 (6)
C2—C3—O2—C35	158.1 (5)
O2—C3—C4—C34	−57.0 (6)
C2—C3—C4—C34	63.8 (7)
O2—C3—C4—C5	174.7 (5)
C2—C3—C4—C5	−64.5 (6)
C34—C4—C5—O4	42.3 (6)
C14—C15—C16—C30	129.9 (6)
C12—C13—O6—C16	−158.5 (5)
C14—C13—O6—C16	−34.0 (6)
C17—C16—O6—C13	135.7 (5)
C30—C16—O6—C13	−107.8 (5)
C15—C16—O6—C13	14.1 (6)
O6—C16—C17—O7	−72.8 (6)
C30—C16—C17—O7	170.7 (5)
C15—C16—C17—O7	42.9 (7)
O6—C16—C17—C18	164.6 (5)
C30—C16—C17—C18	48.2 (7)
C15—C16—C17—C18	−79.7 (7)
O7—C17—C18—C19	40.4 (5)
C16—C17—C18—C19	166.2 (5)
O7—C17—C18—C29	−77.3 (6)
C16—C17—C18—C29	48.5 (8)
C29—C18—C19—C20	82.1 (6)
C17—C18—C19—C20	−41.3 (6)
C18—C19—C20—O7	29.1 (6)

C3—C4—C5—O4	169.6 (4)	C18—C19—C20—C21	152.2 (5)
C34—C4—C5—C6	167.3 (5)	C16—C17—O7—C20	−153.3 (5)
C3—C4—C5—C6	−65.3 (6)	C18—C17—O7—C20	−23.6 (5)
O4—C5—C6—C7	−58.4 (6)	C21—C20—O7—C17	−128.9 (5)
C4—C5—C6—C7	177.5 (5)	C19—C20—O7—C17	−3.6 (6)
O4—C5—C6—C33	64.9 (6)	O7—C20—C21—O8	51.9 (6)
C4—C5—C6—C33	−59.2 (6)	C19—C20—C21—O8	−68.0 (7)
C5—C6—C7—O3	−71.7 (6)	O7—C20—C21—C22	173.3 (5)
C33—C6—C7—O3	164.7 (5)	C19—C20—C21—C22	53.4 (7)
C5—C6—C7—C8	51.7 (6)	O8—C21—C22—C28	180.0 (5)
C33—C6—C7—C8	−71.8 (6)	C20—C21—C22—C28	60.7 (7)
O3—C7—C8—C9	73.1 (6)	O8—C21—C22—C23	−55.7 (6)
C6—C7—C8—C9	−49.8 (6)	C20—C21—C22—C23	−175.0 (5)
C7—C8—C9—O4	53.5 (6)	C21—C22—C23—C24	53.6 (7)
C7—C8—C9—O5	−65.9 (6)	C28—C22—C23—C24	179.2 (5)
C7—C8—C9—C10	177.7 (5)	C22—C23—C24—C25	−54.1 (7)
O5—C9—O4—C5	59.5 (6)	C22—C23—C24—C27	−178.8 (5)
C8—C9—O4—C5	−59.9 (6)	C23—C24—C25—O9	−65.0 (6)
C10—C9—O4—C5	172.4 (5)	C27—C24—C25—O9	58.1 (7)
C6—C5—O4—C9	63.0 (6)	C23—C24—C25—O8	55.9 (6)
C4—C5—O4—C9	−168.9 (5)	C27—C24—C25—O8	179.1 (5)
O4—C9—C10—C11	−86.4 (6)	C23—C24—C25—C26	170.1 (5)
O5—C9—C10—C11	29.2 (6)	C27—C24—C25—C26	−66.8 (7)
C8—C9—C10—C11	147.9 (5)	C22—C21—O8—C25	62.3 (6)
C9—C10—C11—C12	−20.5 (6)	C20—C21—O8—C25	−174.6 (4)
C10—C11—C12—O5	4.6 (6)	O9—C25—O8—C21	56.9 (7)
C10—C11—C12—C13	−111.0 (6)	C26—C25—O8—C21	178.3 (4)
C10—C11—C12—C32	123.9 (5)	C24—C25—O8—C21	−61.8 (6)
O4—C9—O5—C12	88.1 (5)	C1—N1—C26—C25	−112.6 (6)
C8—C9—O5—C12	−150.6 (4)	C37—N1—C26—C25	79.9 (6)
C10—C9—O5—C12	−27.6 (6)	O9—C25—C26—N1	49.4 (6)
C13—C12—O5—C9	132.2 (5)	O8—C25—C26—N1	−71.1 (6)
C32—C12—O5—C9	−104.7 (5)	C24—C25—C26—N1	171.6 (5)
C11—C12—O5—C9	14.5 (6)	O6—C16—C30—C31	53.5 (7)
O5—C12—C13—O6	71.8 (6)	C17—C16—C30—C31	168.2 (5)
C32—C12—C13—O6	−50.1 (7)	C15—C16—C30—C31	−63.4 (7)
C11—C12—C13—O6	−174.1 (5)	C1—N1—C37—C38	−82.8 (7)
O5—C12—C13—C14	−44.4 (7)	C26—N1—C37—C38	83.6 (7)
C32—C12—C13—C14	−166.3 (5)	N1—C37—C38—O10	−4.0 (9)
C11—C12—C13—C14	69.8 (7)	N1—C37—C38—N2	175.0 (5)
O6—C13—C14—C15	39.8 (6)	O10—C38—N2—C39	7.1 (11)
C12—C13—C14—C15	159.7 (5)	C37—C38—N2—C39	−171.9 (6)
C13—C14—C15—C16	−31.1 (6)	C38—N2—C39—C42	54.4 (9)
C14—C15—C16—O6	11.2 (6)	C38—N2—C39—C40	173.5 (6)
C14—C15—C16—C17	−104.7 (6)	C38—N2—C39—C41	−67.6 (8)

Hydrogen-bond geometry (Å, º)

D—H···*A*	*D*—H	H···*A*	*D*···*A*	*D*—H···*A*
O3—H3*O*···O5	0.84	2.11	2.796 (6)	139
O9—H9*O*···O3	0.84	1.93	2.746 (5)	164
N2—H2*N*···O6	0.86	2.45	3.300 (6)	170
