# Supplementary material for: Access to Cyclic Monensin Derivatives via a Four-Component Ugi Reaction
Source: J Org Chem. 2026 Jul 4;91(28):9933–9. doi: 10.1021/acs.joc.6c01246 (PMC13386529; doi:10.1021/acs.joc.6c01246)

checkCIF/PLATON report

Structure factors have been supplied for datablock(s) MON_UGI_7_RT

THIS REPORT IS FOR GUIDANCE ONLY. IF USED AS PART OF A REVIEW PROCEDURE FOR PUBLICATION, IT SHOULD NOT REPLACE THE EXPERTISE OF AN EXPERIENCED CRYSTALLOGRAPHIC REFEREE.

No syntax errors found. CIF dictionary Interpreting this report

Datablock: MON_UGI_7_RT

```
Bond precision:    C-C = 0.0052 A           Wavelength=0.71073

Cell:              a=10.6322(5)      b=11.7204(6)      c=36.0543(18)
                   alpha=90          beta=90           gamma=90
Temperature:       295 K
```

	Calculated	Reported
Volume	4492.9(4)	4492.9(4)
Space group	P 21 21 21	P 21 21 21
Hall group	P 2ac 2ab	P 2ac 2ab
Moiety formula	C44 H74 N2 O10	C44 H74 N2 O10
Sum formula	C44 H74 N2 O10	C44 H74 N2 O10
Mr	791.05	791.05
Dx,g cm-3	1.169	1.169
Z	4	4
Mu (mm-1)	0.082	0.082
F000	1728.0	1728.0
F000'	1728.83	
h,k,lmax	14,15,49	13,15,48
Nref	11953[6636]	10944
Tmin,Tmax	0.976,0.983	0.990,1.000
Tmin'	0.974	

```
Correction method= # Reported T Limits: Tmin=0.990 Tmax=1.000
AbsCorr = MULTI-SCAN
```

Data completeness= 1.65/0.92   Theta(max)= 28.998

R(reflections)= 0.0574(5434)   wR2(reflections)= 0.1073(10944)

S = 1.002   Npar= 519

The following ALERTS were generated. Each ALERT has the format
test-name_ALERT_alert-type_alert-level.
Click on the hyperlinks for more details of the test.

Alert level C

```
STRVA01_ALERT_4_C           Flack parameter is too small
           From the CIF: _refine_ls_abs_structure_Flack   -0.300
           From the CIF: _refine_ls_abs_structure_Flack_su    0.500
PLAT220_ALERT_2_C NonSolvent   Resd 1  C   Ueq(max)/Ueq(min) Range        4.6 Ratio
PLAT222_ALERT_3_C NonSolvent Resd 1  H   Uiso(max)/Uiso(min) Range        5.7 Ratio
PLAT230_ALERT_2_C Hirshfeld Test Diff for    O2       --C35      .        7.0 s.u.
PLAT241_ALERT_2_C High  MainResAtom  Ueq as Compared to Neighbours        C11 Check
PLAT241_ALERT_2_C High  MainResAtom  Ueq as Compared to Neighbours        C41 Check
PLAT241_ALERT_2_C High  MainResAtom  Ueq as Compared to Neighbours        C42 Check
PLAT242_ALERT_2_C Low   MainResAtom  Ueq as Compared to Neighbours         C2 Check
PLAT340_ALERT_3_C Low Bond Precision on  C-C Bonds ...............     0.0052 Ang.
PLAT906_ALERT_3_C Large K Value in the Analysis of Variance ......      8.214 Check
PLAT906_ALERT_3_C Large K Value in the Analysis of Variance ......      2.512 Check
PLAT910_ALERT_3_C Missing FCF Reflection(s) Below Theta(Min)[Deg]=       2.56 Note
               1  0  1,   0  1  1,   0  0  2,   1  0  2,   0  1  2,   0  1  3,
               0  0  4,
PLAT911_ALERT_3_C Missing FCF Refl Between Thmin & STh/L=    0.600          2 Report
               1  1  0,   0 14  0,
```

Alert level G

```
PLAT002_ALERT_2_G Number of Distance or Angle Restraints on AtSite          2 Note
PLAT007_ALERT_5_G Number of Unrefined Donor-H Atoms ..............          2 Report
             H3      H91
PLAT032_ALERT_4_G Std. Uncertainty on Flack Parameter Value High .      0.500 Report
PLAT172_ALERT_4_G The CIF-Embedded .res File Contains DFIX Records          1 Report
PLAT398_ALERT_2_G Deviating  C-O-C   Angle From 120 for O6       .      107.9 Degree
PLAT398_ALERT_2_G Deviating  C-O-C   Angle From 120 for O7       .      109.3 Degree
PLAT791_ALERT_4_G Model has Chirality at C2         (Sohncke SpGr)          S Verify
PLAT791_ALERT_4_G Model has Chirality at C3         (Sohncke SpGr)          R Verify
PLAT791_ALERT_4_G Model has Chirality at C4         (Sohncke SpGr)          S Verify
PLAT791_ALERT_4_G Model has Chirality at C5         (Sohncke SpGr)          S Verify
PLAT791_ALERT_4_G Model has Chirality at C6         (Sohncke SpGr)          R Verify
PLAT791_ALERT_4_G Model has Chirality at C7         (Sohncke SpGr)          S Verify
PLAT791_ALERT_4_G Model has Chirality at C9         (Sohncke SpGr)          R Verify
PLAT791_ALERT_4_G Model has Chirality at C12        (Sohncke SpGr)          S Verify
PLAT791_ALERT_4_G Model has Chirality at C13        (Sohncke SpGr)          R Verify
PLAT791_ALERT_4_G Model has Chirality at C16        (Sohncke SpGr)          S Verify
PLAT791_ALERT_4_G Model has Chirality at C17        (Sohncke SpGr)          R Verify
PLAT791_ALERT_4_G Model has Chirality at C18        (Sohncke SpGr)          S Verify
PLAT791_ALERT_4_G Model has Chirality at C20        (Sohncke SpGr)          R Verify
PLAT791_ALERT_4_G Model has Chirality at C21        (Sohncke SpGr)          S Verify
```

```
PLAT791_ALERT_4_G Model has Chirality at C22        (Sohncke SpGr)          S Verify
PLAT791_ALERT_4_G Model has Chirality at C24        (Sohncke SpGr)          R Verify
PLAT791_ALERT_4_G Model has Chirality at C25        (Sohncke SpGr)          R Verify
PLAT860_ALERT_3_G Number of Least-Squares Restraints .............          1 Note
PLAT899_ALERT_4_G SHELXL2018 is Outdated   and Succeeded by SHELXL     2019/3 Note
PLAT912_ALERT_4_G Missing # of FCF Reflections Above STh/L=  0.600        267 Note
PLAT916_ALERT_2_G Hooft y and Flack x Parameter Values Differ by .       0.20 Check
PLAT961_ALERT_5_G Dataset Contains no Negative Intensities .......     Please Check
PLAT965_ALERT_2_G The SHELXL WEIGHT Optimisation has not Converged     Please Check
PLAT967_ALERT_5_G Note: Two-Theta Cutoff Value in Embedded .res ..       58.0 Degree
PLAT969_ALERT_5_G The 'Henn et al.' R-Factor-gap value ..........       2.358 Note
              Predicted wR2: Based on SigI**2  4.55 or SHELX Weight 10.71
PLAT978_ALERT_2_G Number C-C Bonds with Positive Residual Density.          0 Info
PLAT994_ALERT_1_G SHELXL .ins Contains no or MERG 0 Instruction ..          ! Note
```

```
   0 ALERT level A = Most likely a serious problem - resolve or explain
   0 ALERT level B = A potentially serious problem, consider carefully
  13 ALERT level C = Check. Ensure it is not caused by an omission or oversight
  33 ALERT level G = General information/check it is not something unexpected

   1 ALERT type 1 CIF construction/syntax error, inconsistent or missing data
  12 ALERT type 2 Indicator that the structure model may be wrong or deficient
   7 ALERT type 3 Indicator that the structure quality may be low
  22 ALERT type 4 Improvement, methodology, query or suggestion
   4 ALERT type 5 Informative message, check
```

It is advisable to attempt to resolve as many as possible of the alerts in all categories. Often the minor alerts point to easily fixed oversights, errors and omissions in your CIF or refinement strategy, so attention to these fine details can be worthwhile. It is up to the individual to critically assess their own results and, if necessary, seek expert advice.

PLATON version of 15/01/2026; check.def file version of 02/01/2026

---

duplicate check

No duplication found

Datablock MON_UGI_7_RT - ellipsoid plot

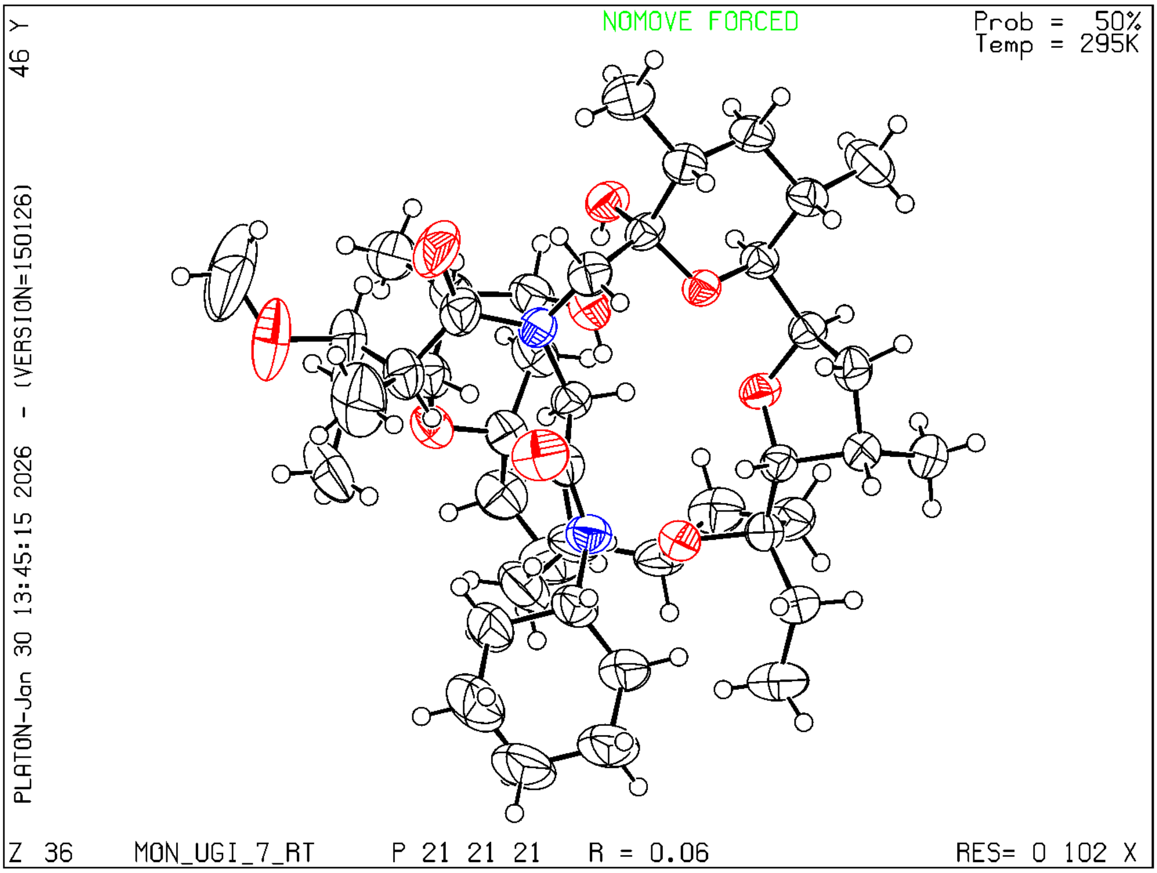

Supplement: Supplementary file 1 [file jo6c01246_si_001.zip › Compouds data/Compound 5/scXRD/checkcif_UGI_7_5.pdf]
