# Supplementary material for: Access to Cyclic Monensin Derivatives via a Four-Component Ugi Reaction
Source: J Org Chem. 2026 Jul 4;91(28):9933–9. doi: 10.1021/acs.joc.6c01246 (PMC13386529; doi:10.1021/acs.joc.6c01246)
Supplement: Supplementary file 1 [file jo6c01246_si_001.zip › Compouds data/Compound 5/scXRD/printcif_MON_UGI_7_RT.pdf]

Title

Enter author details here

Abstract

Table 1

Experimental details

Crystal data	
Chemical formula	$C_{44}H_{74}N_2O_{10}$
M_r	791.05
Crystal system, space group	Orthorhombic, $P2_12_12_1$
Temperature (K)	295
a, *b*, *c* (Å)	10.6322 (5), 11.7204 (6), 36.0543 (18)
V (Å^3)	4492.9 (4)
Z	4
Radiation type	Mo $K\alpha$
μ (mm^{-1})	0.08
Crystal size (mm)	0.32 × 0.24 × 0.21
Data collection	
Diffractometer	Xcalibur, Atlas
Absorption correction	Multi-scan<br>*CrysAlis PRO* 1.171.42.93a (Rigaku Oxford Diffraction, 2023) Empirical absorption correction using spherical harmonics, implemented in SCALE3 ABSPACK scaling algorithm.
T_{min}, T_{max}	0.990, 1.000
No. of measured, independent and observed [$I > 2\sigma(I)$] reflections	34323, 10944, 5434
R_{int}	0.048
(sin $\theta/\lambda)_{max}$ (Å^{-1})	0.682
Refinement	
$R[F^2 > 2\sigma(F^2)]$, $wR(F^2)$, S	0.057, 0.107, 1.00
No. of reflections	10944
No. of parameters	519
No. of restraints	1
H-atom treatment	H atoms treated by a mixture of independent and constrained refinement
$\Delta\rho_{max}$, $\Delta\rho_{min}$ (e Å^{-3})	0.13, −0.13
Absolute structure	Flack x determined using 1641 quotients [(I+)-(I-)]/[(I+)+(I-)] (Parsons, Flack and Wagner, Acta Cryst. B69 (2013) 249-259).
Absolute structure parameter	−0.3 (5)

Computer programs: *CrysAlis PRO* 1.171.42.93a (Rigaku Oxford Diffraction, 2023), *SHELXT* 2014/5 (Sheldrick, 2014), *SHELXL2018*/3 (Sheldrick, 2018), Brandenburg & Putz (2006). Diamond 3.0. Crystal and Molecular Structure Visualisation, University of Bonn, Germany.

Table 2

Hydrogen-bond geometry (Å, º)

D—H···*A*	*D*—H	H···*A*	*D*···*A*	*D*—H···*A*
O3—H3···O5	0.82	2.12	2.787 (3)	139
C14—H14*B*···O1^{i}	0.97	2.53	3.410 (5)	152
O9—H91···O3	0.82	2.06	2.870 (3)	169
C37—H37*A*···O8	0.97	2.23	2.986 (4)	134
N2—H2···O6	0.85 (3)	2.55 (3)	3.385 (4)	168 (3)

Symmetry code: (i) *x*, *y*+1, *z*.

Acknowledgements

Funding information

References

Figure 1

supporting information

Title

Computing details

Data collection: *CrysAlis PRO* 1.171.42.93a (Rigaku Oxford Diffraction, 2023); cell refinement: *CrysAlis PRO* 1.171.42.93a (Rigaku Oxford Diffraction, 2023); data reduction: *CrysAlis PRO* 1.171.42.93a (Rigaku Oxford Diffraction, 2023); program(s) used to solve structure: *SHELXT* 2014/5 (Sheldrick, 2014); program(s) used to refine structure: *SHELXL2018*/3 (Sheldrick, 2018); molecular graphics: Brandenburg & Putz (2006). Diamond 3.0. Crystal and Molecular Structure Visualisation, University of Bonn, Germany.

(MON_UGI_7_RT)

Crystal data

$C_{44}H_{74}N_2O_{10}$
M_r = 791.05
Orthorhombic, $P2_12_12_1$
a = 10.6322 (5) Å
b = 11.7204 (6) Å
c = 36.0543 (18) Å
V = 4492.9 (4) Å^3
Z = 4
F(000) = 1728
D_x = 1.169 Mg m^{-3}
Mo $K\alpha$ radiation, λ = 0.71073 Å
Cell parameters from 8290 reflections
θ = 3.1–27.5°
μ = 0.08 mm^{-1}
T = 295 K
Parallelepiped, colourless
0.32 × 0.24 × 0.21 mm

Data collection

Xcalibur, Atlas diffractometer
Radiation source: fine-focus sealed X-ray tube
Detector resolution: 10.6249 pixels mm^{-1}
ω–scan
Absorption correction: multi-scan *CrysAlis PRO* 1.171.42.93a (Rigaku Oxford Diffraction, 2023) Empirical absorption correction using spherical harmonics, implemented in SCALE3 ABSPACK scaling algorithm.
T_{min} = 0.990, T_{max} = 1.000
34323 measured reflections
10944 independent reflections
5434 reflections with $I > 2\sigma(I)$
R_{int} = 0.048
θ_{max} = 29.0°, θ_{min} = 2.6°
h = −13→13
k = −14→15
l = −48→33

Refinement

Refinement on F^2
Least-squares matrix: full
$R[F^2 > 2\sigma(F^2)]$ = 0.057
$wR(F^2)$ = 0.107
S = 1.00
10944 reflections
519 parameters
1 restraint
Primary atom site location: structure-invariant direct methods
Secondary atom site location: difference Fourier map
Hydrogen site location: mixed
H atoms treated by a mixture of independent and constrained refinement
$w = 1/[\sigma^2(F_o^2) + (0.0317P)^2]$ where $P = (F_o^2 + 2F_c^2)/3$
$(\Delta/\sigma)_{max}$ < 0.001
$\Delta\rho_{max}$ = 0.13 e Å^{-3}
$\Delta\rho_{min}$ = −0.13 e Å^{-3}
Absolute structure: Flack x determined using 1641 quotients [(I+)-(I-)]/[(I+)+(I-)] (Parsons, Flack and Wagner, Acta Cryst. B69 (2013) 249-259).
Absolute structure parameter: −0.3 (5)

Special details

Geometry. All e.s.d.'s (except the e.s.d. in the dihedral angle between two l.s. planes) are estimated using the full covariance matrix. The cell e.s.d.'s are taken into account individually in the estimation of e.s.d.'s in distances, angles and torsion angles; correlations between e.s.d.'s in cell parameters are only used when they are defined by crystal symmetry. An approximate (isotropic) treatment of cell e.s.d.'s is used for estimating e.s.d.'s involving l.s. planes.

Refinement. Refinement of F^2 against ALL reflections. The weighted *R*-factor *wR* and goodness of fit *S* are based on F^2, conventional *R*-factors *R* are based on *F*, with *F* set to zero for negative F^2. The threshold expression of $F^2 > \sigma(F^2)$ is used only for calculating *R*-factors(gt) *etc*. and is not relevant to the choice of reflections for refinement. *R*-factors based on F^2 are statistically about twice as large as those based on *F*, and *R*- factors based on ALL data will be even larger.

Fractional atomic coordinates and isotropic or equivalent isotropic displacement parameters (Å^2)

	x	*y*	*z*	U_{iso}*/U_{eq}
C1	0.6144 (3)	0.2406 (3)	0.37093 (10)	0.0641 (10)
O1	0.5781 (3)	0.1480 (2)	0.38266 (9)	0.1069 (11)
C2	0.5735 (3)	0.2817 (3)	0.33280 (9)	0.0662 (10)
H2A	0.602495	0.360143	0.328848	0.079*
C3	0.4252 (3)	0.2771 (3)	0.33055 (11)	0.0766 (12)
H3A	0.391516	0.252012	0.354499	0.092*
O2	0.3890 (3)	0.1983 (3)	0.30262 (10)	0.1321 (14)
C4	0.3696 (4)	0.3956 (3)	0.32078 (10)	0.0766 (11)
H4	0.278645	0.385375	0.317930	0.092*
C5	0.3893 (3)	0.4783 (3)	0.35336 (9)	0.0589 (9)
H5	0.480017	0.483911	0.357885	0.071*
C6	0.3261 (3)	0.4462 (3)	0.39019 (9)	0.0633 (10)
H6	0.363615	0.374511	0.398733	0.076*
C7	0.3569 (3)	0.5379 (3)	0.41882 (9)	0.0622 (10)
H7	0.305608	0.522867	0.440859	0.075*
O3	0.48640 (19)	0.5300 (2)	0.42963 (6)	0.0621 (6)
H3	0.527263	0.579014	0.418775	0.093*
C8	0.3226 (3)	0.6555 (3)	0.40471 (9)	0.0644 (10)
H8A	0.353029	0.712442	0.422055	0.077*
H8B	0.231773	0.662168	0.403425	0.077*
C9	0.3784 (3)	0.6790 (3)	0.36644 (9)	0.0571 (9)
O4	0.34577 (19)	0.5896 (2)	0.34155 (6)	0.0591 (6)
C10	0.3405 (3)	0.7918 (3)	0.34827 (11)	0.0716 (11)
H10A	0.296541	0.777732	0.325148	0.086*
H10B	0.285501	0.834764	0.364572	0.086*
C11	0.4593 (3)	0.8569 (4)	0.34121 (13)	0.1009 (15)
H11A	0.465237	0.921967	0.357755	0.121*
H11B	0.461771	0.884117	0.315807	0.121*
C12	0.5674 (3)	0.7725 (3)	0.34848 (10)	0.0665 (11)
O5	0.51152 (18)	0.68371 (19)	0.37114 (6)	0.0564 (6)
C13	0.6696 (3)	0.8292 (3)	0.37120 (10)	0.0649 (10)
H13	0.703862	0.892608	0.356652	0.078*
C14	0.6365 (4)	0.8739 (3)	0.40932 (11)	0.0746 (11)
H14A	0.582396	0.820987	0.422476	0.090*
H14B	0.594889	0.947445	0.407730	0.090*
C15	0.7634 (3)	0.8844 (3)	0.42812 (10)	0.0680 (10)
H15A	0.756298	0.869205	0.454487	0.082*
H15B	0.798029	0.960187	0.424665	0.082*
C16	0.8462 (3)	0.7942 (3)	0.40912 (9)	0.0542 (9)
O6	0.77004 (18)	0.74943 (18)	0.37818 (5)	0.0531 (6)

C17	0.8778 (2)	0.6916 (3)	0.43341 (8)	0.0458 (7)
H17	0.902934	0.629136	0.416913	0.055*
C18	0.9823 (3)	0.7053 (3)	0.46298 (8)	0.0553 (9)
H18	1.065299	0.698381	0.451350	0.066*
C19	0.9539 (3)	0.5990 (3)	0.48601 (9)	0.0573 (9)
H19A	0.983523	0.530439	0.473707	0.069*
H19B	0.991873	0.603825	0.510420	0.069*
C20	0.8110 (3)	0.6009 (2)	0.48843 (8)	0.0469 (8)
H20	0.786997	0.650931	0.509021	0.056*
O7	0.76946 (16)	0.65338 (16)	0.45418 (5)	0.0484 (5)
C21	0.7486 (3)	0.4853 (2)	0.49437 (8)	0.0444 (7)
H21	0.657526	0.496071	0.496522	0.053*
C22	0.7966 (3)	0.4233 (3)	0.52887 (8)	0.0535 (8)
H22	0.887743	0.413601	0.526367	0.064*
C23	0.7367 (3)	0.3050 (3)	0.53005 (9)	0.0593 (9)
H23A	0.774220	0.261802	0.550147	0.071*
H23B	0.647701	0.313158	0.535410	0.071*
C24	0.7518 (3)	0.2381 (3)	0.49411 (9)	0.0568 (9)
H24	0.841060	0.219283	0.491180	0.068*
C25	0.7117 (3)	0.3103 (3)	0.46079 (8)	0.0458 (7)
O8	0.77474 (17)	0.41859 (16)	0.46210 (5)	0.0438 (5)
O9	0.58095 (17)	0.32470 (19)	0.46289 (6)	0.0564 (6)
H91	0.560373	0.381223	0.450938	0.085*
C26	0.7524 (3)	0.2565 (3)	0.42419 (9)	0.0557 (8)
H26A	0.843019	0.263386	0.421957	0.067*
H26B	0.732266	0.175841	0.424838	0.067*
N1	0.6944 (2)	0.3067 (2)	0.39141 (7)	0.0499 (7)
C27	0.6775 (4)	0.1256 (3)	0.49654 (11)	0.0892 (13)
H27A	0.591206	0.141819	0.502284	0.134*
H27B	0.712886	0.078460	0.515656	0.134*
H27C	0.682232	0.086414	0.473203	0.134*
C28	0.7713 (4)	0.4875 (3)	0.56479 (9)	0.0871 (12)
H28A	0.812173	0.560493	0.563928	0.131*
H28B	0.803434	0.444343	0.585336	0.131*
H28C	0.682348	0.498186	0.567752	0.131*
C29	0.9778 (3)	0.8120 (3)	0.48741 (9)	0.0753 (11)
H29A	0.896635	0.817531	0.498943	0.113*
H29B	0.992346	0.878379	0.472435	0.113*
H29C	1.041539	0.806882	0.506198	0.113*
C30	0.9697 (3)	0.8411 (3)	0.39273 (10)	0.0753 (11)
H30A	1.019071	0.872668	0.412896	0.090*
H30B	1.016776	0.777487	0.382574	0.090*
C31	0.9585 (4)	0.9309 (4)	0.36301 (12)	0.1123 (17)
H31A	0.916044	0.899420	0.341854	0.168*
H31B	1.040882	0.956098	0.355849	0.168*
H31C	0.911276	0.994542	0.372390	0.168*
C32	0.6172 (4)	0.7194 (4)	0.31310 (10)	0.0973 (15)
H32A	0.671930	0.656994	0.319163	0.146*
H32B	0.663143	0.775655	0.299264	0.146*
H32C	0.548150	0.691973	0.298453	0.146*
C33	0.1837 (3)	0.4276 (4)	0.38679 (11)	0.0928 (14)
H33A	0.146470	0.491624	0.374341	0.139*

H33B	0.147762	0.420148	0.411091	0.139*
H33C	0.167918	0.359373	0.372817	0.139*
C34	0.4188 (4)	0.4428 (4)	0.28406 (11)	0.1067 (16)
H34A	0.371669	0.509605	0.277415	0.160*
H34B	0.409647	0.386093	0.265042	0.160*
H34C	0.506033	0.462422	0.286653	0.160*
C35	0.2924 (7)	0.1274 (5)	0.3126 (2)	0.203 (4)
H35A	0.277497	0.073224	0.293105	0.304*
H35B	0.217701	0.171624	0.316694	0.304*
H35C	0.314313	0.087621	0.334947	0.304*
C36	0.6354 (4)	0.2015 (4)	0.30404 (13)	0.1207 (18)
H36A	0.725035	0.210859	0.304834	0.181*
H36B	0.605183	0.220072	0.279686	0.181*
H36C	0.614322	0.123830	0.309761	0.181*
C37	0.7436 (3)	0.4167 (3)	0.37981 (8)	0.0506 (8)
H37A	0.773715	0.457197	0.401527	0.061*
H37B	0.675585	0.461048	0.369171	0.061*
C38	0.8500 (3)	0.4094 (3)	0.35167 (9)	0.0542 (8)
O10	0.8973 (2)	0.3193 (2)	0.34223 (7)	0.0803 (8)
N2	0.8859 (3)	0.5105 (3)	0.33803 (8)	0.0585 (8)
H2	0.856 (3)	0.574 (3)	0.3448 (10)	0.070*
C39	0.9703 (3)	0.5212 (3)	0.30604 (9)	0.0648 (10)
H39	1.041587	0.469375	0.309641	0.078*
C40	0.9027 (4)	0.4871 (4)	0.27103 (10)	0.0926 (14)
H40A	0.827937	0.533695	0.268085	0.111*
H40B	0.876386	0.408028	0.272897	0.111*
C41	0.9865 (5)	0.5016 (6)	0.23761 (12)	0.134 (2)
H41A	1.056742	0.449043	0.239447	0.161*
H41B	0.939499	0.482601	0.215399	0.161*
C42	1.0353 (5)	0.6197 (6)	0.23437 (13)	0.139 (2)
H42A	0.965878	0.671689	0.229901	0.167*
H42B	1.092286	0.624499	0.213427	0.167*
C43	1.1035 (4)	0.6548 (5)	0.26929 (12)	0.1122 (18)
H43A	1.129931	0.733682	0.267130	0.135*
H43B	1.178180	0.608114	0.272237	0.135*
C44	1.0204 (3)	0.6415 (4)	0.30315 (10)	0.0834 (12)
H44A	1.068369	0.659639	0.325263	0.100*
H44B	0.950606	0.694604	0.301578	0.100*

Atomic displacement parameters (Å^2)

	U^{11}	U^{22}	U^{33}	U^{12}	U^{13}	U^{23}
C1	0.078 (2)	0.045 (2)	0.069 (3)	−0.003 (2)	−0.011 (2)	−0.006 (2)
O1	0.137 (2)	0.0554 (17)	0.128 (3)	−0.0378 (18)	−0.0536 (19)	0.0195 (18)
C2	0.078 (2)	0.060 (2)	0.060 (2)	0.003 (2)	−0.0051 (18)	−0.018 (2)
C3	0.085 (3)	0.071 (3)	0.074 (3)	−0.004 (2)	−0.020 (2)	−0.026 (2)
O2	0.122 (3)	0.123 (3)	0.151 (3)	−0.020 (2)	−0.043 (2)	−0.074 (3)
C4	0.087 (3)	0.082 (3)	0.061 (3)	0.003 (2)	−0.020 (2)	−0.019 (2)
C5	0.061 (2)	0.058 (2)	0.058 (2)	0.0038 (19)	−0.0091 (17)	0.0004 (19)
C6	0.066 (2)	0.067 (3)	0.056 (2)	−0.0042 (19)	−0.0068 (17)	0.010 (2)
C7	0.060 (2)	0.078 (3)	0.049 (2)	−0.001 (2)	0.0043 (16)	0.004 (2)
O3	0.0646 (14)	0.0704 (18)	0.0512 (15)	−0.0031 (12)	−0.0097 (11)	0.0064 (12)

C8	0.058 (2)	0.082 (3)	0.053 (2)	0.013 (2)	0.0034 (16)	−0.001 (2)
C9	0.054 (2)	0.062 (2)	0.055 (2)	0.0094 (19)	−0.0030 (16)	−0.002 (2)
O4	0.0590 (13)	0.0690 (17)	0.0492 (14)	0.0066 (13)	−0.0074 (10)	0.0048 (13)
C10	0.075 (2)	0.062 (3)	0.078 (3)	0.020 (2)	−0.009 (2)	0.012 (2)
C11	0.078 (3)	0.101 (4)	0.123 (4)	0.011 (3)	−0.009 (2)	0.057 (3)
C12	0.061 (2)	0.079 (3)	0.060 (2)	0.008 (2)	−0.0023 (18)	0.031 (2)
O5	0.0512 (13)	0.0576 (15)	0.0603 (14)	0.0037 (11)	−0.0057 (10)	0.0137 (12)
C13	0.068 (2)	0.059 (2)	0.068 (3)	0.000 (2)	0.0001 (18)	0.027 (2)
C14	0.093 (3)	0.042 (2)	0.088 (3)	0.013 (2)	0.016 (2)	0.002 (2)
C15	0.092 (3)	0.041 (2)	0.072 (3)	−0.005 (2)	0.006 (2)	0.0006 (19)
C16	0.067 (2)	0.050 (2)	0.045 (2)	−0.0107 (18)	0.0026 (16)	−0.0038 (17)
O6	0.0557 (12)	0.0538 (14)	0.0497 (13)	0.0011 (12)	0.0004 (10)	0.0021 (11)
C17	0.0481 (17)	0.0435 (19)	0.0459 (18)	−0.0045 (15)	0.0034 (14)	−0.0035 (16)
C18	0.0547 (18)	0.063 (2)	0.048 (2)	−0.0142 (17)	−0.0003 (15)	−0.0001 (18)
C19	0.051 (2)	0.063 (2)	0.058 (2)	−0.0038 (17)	−0.0116 (15)	0.0007 (19)
C20	0.055 (2)	0.041 (2)	0.0445 (19)	−0.0020 (16)	−0.0003 (14)	0.0008 (16)
O7	0.0495 (12)	0.0422 (12)	0.0536 (13)	−0.0042 (10)	0.0011 (10)	0.0086 (11)
C21	0.0491 (17)	0.0405 (18)	0.0437 (19)	0.0010 (15)	0.0007 (14)	0.0013 (15)
C22	0.062 (2)	0.050 (2)	0.048 (2)	−0.0009 (17)	−0.0022 (15)	0.0069 (17)
C23	0.070 (2)	0.052 (2)	0.056 (2)	−0.0039 (19)	−0.0097 (17)	0.0227 (18)
C24	0.065 (2)	0.045 (2)	0.061 (2)	0.0028 (18)	−0.0089 (17)	0.0120 (18)
C25	0.0458 (19)	0.0334 (17)	0.058 (2)	−0.0014 (15)	−0.0027 (14)	0.0044 (16)
O8	0.0523 (11)	0.0335 (11)	0.0456 (12)	−0.0012 (10)	0.0022 (9)	−0.0002 (10)
O9	0.0485 (12)	0.0549 (15)	0.0657 (15)	−0.0041 (11)	−0.0035 (10)	0.0096 (12)
C26	0.066 (2)	0.0366 (18)	0.065 (2)	0.0073 (18)	−0.0037 (18)	−0.0006 (17)
N1	0.0656 (16)	0.0347 (15)	0.0495 (16)	−0.0058 (14)	−0.0006 (13)	−0.0046 (13)
C27	0.133 (3)	0.048 (2)	0.087 (3)	−0.018 (2)	−0.017 (3)	0.023 (2)
C28	0.138 (3)	0.076 (3)	0.046 (2)	−0.002 (3)	−0.007 (2)	0.003 (2)
C29	0.101 (3)	0.067 (3)	0.058 (2)	−0.032 (2)	−0.0052 (19)	−0.006 (2)
C30	0.078 (2)	0.089 (3)	0.059 (2)	−0.035 (2)	0.0061 (18)	0.009 (2)
C31	0.120 (4)	0.126 (4)	0.091 (3)	−0.044 (3)	0.010 (3)	0.043 (3)
C32	0.078 (2)	0.165 (5)	0.049 (2)	−0.009 (3)	−0.0079 (19)	0.018 (3)
C33	0.075 (3)	0.123 (4)	0.080 (3)	−0.024 (3)	−0.012 (2)	0.019 (3)
C34	0.153 (4)	0.118 (4)	0.049 (3)	0.022 (3)	−0.021 (2)	−0.017 (3)
C35	0.218 (7)	0.101 (5)	0.289 (9)	−0.042 (6)	−0.120 (7)	−0.044 (6)
C36	0.117 (3)	0.131 (4)	0.114 (4)	0.015 (3)	0.014 (3)	−0.066 (4)
C37	0.062 (2)	0.0411 (19)	0.0489 (19)	−0.0051 (17)	0.0020 (16)	0.0009 (16)
C38	0.058 (2)	0.056 (2)	0.048 (2)	−0.003 (2)	−0.0019 (16)	−0.0061 (19)
O10	0.0879 (17)	0.0651 (18)	0.0880 (19)	0.0119 (16)	0.0221 (14)	−0.0090 (16)
N2	0.0657 (19)	0.058 (2)	0.0520 (19)	−0.0042 (16)	0.0101 (14)	0.0021 (16)
C39	0.060 (2)	0.085 (3)	0.050 (2)	−0.002 (2)	0.0072 (17)	0.004 (2)
C40	0.096 (3)	0.133 (4)	0.049 (3)	−0.029 (3)	0.010 (2)	−0.012 (2)
C41	0.135 (4)	0.211 (7)	0.056 (3)	−0.032 (5)	0.024 (3)	−0.014 (4)
C42	0.112 (4)	0.237 (8)	0.069 (3)	−0.051 (4)	0.018 (3)	0.040 (4)
C43	0.089 (3)	0.166 (5)	0.082 (3)	−0.033 (3)	0.016 (3)	0.036 (3)
C44	0.077 (3)	0.104 (3)	0.069 (3)	−0.022 (3)	0.007 (2)	0.016 (2)

Geometric parameters (Å, º)

C1—O1	1.227 (4)	C23—C24	1.523 (4)
C1—N1	1.367 (4)	C23—H23A	0.9700
C1—C2	1.520 (5)	C23—H23B	0.9700

C2—C36	1.546 (5)	C24—C25	1.530 (4)
C2—C3	1.579 (5)	C24—C27	1.539 (4)
C2—H2A	0.9800	C24—H24	0.9800
C3—O2	1.420 (4)	C25—O9	1.402 (3)
C3—C4	1.549 (5)	C25—O8	1.436 (3)
C3—H3A	0.9800	C25—C26	1.525 (4)
O2—C35	1.369 (7)	O9—H91	0.8200
C4—C34	1.527 (5)	C26—N1	1.457 (4)
C4—C5	1.538 (5)	C26—H26A	0.9700
C4—H4	0.9800	C26—H26B	0.9700
C5—O4	1.449 (4)	N1—C37	1.453 (4)
C5—C6	1.535 (4)	C27—H27A	0.9600
C5—H5	0.9800	C27—H27B	0.9600
C6—C7	1.526 (5)	C27—H27C	0.9600
C6—C33	1.534 (4)	C28—H28A	0.9600
C6—H6	0.9800	C28—H28B	0.9600
C7—O3	1.434 (4)	C28—H28C	0.9600
C7—C8	1.514 (5)	C29—H29A	0.9600
C7—H7	0.9800	C29—H29B	0.9600
O3—H3	0.8200	C29—H29C	0.9600
C8—C9	1.527 (4)	C30—C31	1.507 (5)
C8—H8A	0.9700	C30—H30A	0.9700
C8—H8B	0.9700	C30—H30B	0.9700
C9—O4	1.422 (4)	C31—H31A	0.9600
C9—O5	1.427 (4)	C31—H31B	0.9600
C9—C10	1.530 (5)	C31—H31C	0.9600
C10—C11	1.497 (5)	C32—H32A	0.9600
C10—H10A	0.9700	C32—H32B	0.9600
C10—H10B	0.9700	C32—H32C	0.9600
C11—C12	1.539 (5)	C33—H33A	0.9600
C11—H11A	0.9700	C33—H33B	0.9600
C11—H11B	0.9700	C33—H33C	0.9600
C12—O5	1.450 (4)	C34—H34A	0.9600
C12—C13	1.514 (5)	C34—H34B	0.9600
C12—C32	1.515 (5)	C34—H34C	0.9600
C13—O6	1.441 (4)	C35—H35A	0.9600
C13—C14	1.512 (5)	C35—H35B	0.9600
C13—H13	0.9800	C35—H35C	0.9600
C14—C15	1.515 (5)	C36—H36A	0.9600
C14—H14A	0.9700	C36—H36B	0.9600
C14—H14B	0.9700	C36—H36C	0.9600
C15—C16	1.538 (4)	C37—C38	1.521 (4)
C15—H15A	0.9700	C37—H37A	0.9700
C15—H15B	0.9700	C37—H37B	0.9700
C16—O6	1.475 (3)	C38—O10	1.218 (4)
C16—C17	1.525 (4)	C38—N2	1.338 (4)
C16—C30	1.541 (4)	N2—C39	1.467 (4)
C17—O7	1.445 (3)	N2—H2	0.85 (3)
C17—C18	1.548 (4)	C39—C40	1.507 (5)
C17—H17	0.9800	C39—C44	1.510 (5)
C18—C19	1.528 (4)	C39—H39	0.9800
C18—C29	1.530 (4)	C40—C41	1.509 (5)

C18—H18	0.9800	C40—H40A	0.9700
C19—C20	1.522 (4)	C40—H40B	0.9700
C19—H19A	0.9700	C41—C42	1.483 (7)
C19—H19B	0.9700	C41—H41A	0.9700
C20—O7	1.448 (3)	C41—H41B	0.9700
C20—C21	1.523 (4)	C42—C43	1.510 (6)
C20—H20	0.9800	C42—H42A	0.9700
C21—O8	1.429 (3)	C42—H42B	0.9700
C21—C22	1.528 (4)	C43—C44	1.515 (5)
C21—H21	0.9800	C43—H43A	0.9700
C22—C28	1.522 (4)	C43—H43B	0.9700
C22—C23	1.526 (4)	C44—H44A	0.9700
C22—H22	0.9800	C44—H44B	0.9700

O1—C1—N1	120.7 (3)	C24—C23—H23A	108.9
O1—C1—C2	120.1 (3)	C22—C23—H23A	108.9
N1—C1—C2	119.1 (3)	C24—C23—H23B	108.9
C1—C2—C36	107.0 (3)	C22—C23—H23B	108.9
C1—C2—C3	108.7 (3)	H23A—C23—H23B	107.7
C36—C2—C3	111.7 (3)	C23—C24—C25	110.7 (2)
C1—C2—H2A	109.8	C23—C24—C27	109.8 (3)
C36—C2—H2A	109.8	C25—C24—C27	112.1 (3)
C3—C2—H2A	109.8	C23—C24—H24	108.1
O2—C3—C4	108.5 (3)	C25—C24—H24	108.1
O2—C3—C2	109.2 (3)	C27—C24—H24	108.1
C4—C3—C2	111.2 (3)	O9—C25—O8	110.8 (2)
O2—C3—H3A	109.3	O9—C25—C26	112.2 (2)
C4—C3—H3A	109.3	O8—C25—C26	105.1 (2)
C2—C3—H3A	109.3	O9—C25—C24	107.5 (2)
C35—O2—C3	114.4 (4)	O8—C25—C24	109.4 (2)
C34—C4—C5	112.8 (3)	C26—C25—C24	111.8 (2)
C34—C4—C3	113.0 (3)	C21—O8—C25	114.8 (2)
C5—C4—C3	109.8 (3)	C25—O9—H91	109.5
C34—C4—H4	106.9	N1—C26—C25	114.5 (2)
C5—C4—H4	106.9	N1—C26—H26A	108.6
C3—C4—H4	106.9	C25—C26—H26A	108.6
O4—C5—C6	109.6 (3)	N1—C26—H26B	108.6
O4—C5—C4	107.5 (3)	C25—C26—H26B	108.6
C6—C5—C4	116.5 (3)	H26A—C26—H26B	107.6
O4—C5—H5	107.7	C1—N1—C37	124.9 (3)
C6—C5—H5	107.7	C1—N1—C26	118.2 (3)
C4—C5—H5	107.7	C37—N1—C26	116.0 (3)
C7—C6—C33	111.5 (3)	C24—C27—H27A	109.5
C7—C6—C5	108.6 (3)	C24—C27—H27B	109.5
C33—C6—C5	113.4 (3)	H27A—C27—H27B	109.5
C7—C6—H6	107.7	C24—C27—H27C	109.5
C33—C6—H6	107.7	H27A—C27—H27C	109.5
C5—C6—H6	107.7	H27B—C27—H27C	109.5
O3—C7—C8	112.5 (3)	C22—C28—H28A	109.5
O3—C7—C6	110.1 (3)	C22—C28—H28B	109.5
C8—C7—C6	111.2 (3)	H28A—C28—H28B	109.5
O3—C7—H7	107.6	C22—C28—H28C	109.5

C8—C7—H7	107.6	H28A—C28—H28C	109.5
C6—C7—H7	107.6	H28B—C28—H28C	109.5
C7—O3—H3	109.5	C18—C29—H29A	109.5
C7—C8—C9	112.0 (3)	C18—C29—H29B	109.5
C7—C8—H8A	109.2	H29A—C29—H29B	109.5
C9—C8—H8A	109.2	C18—C29—H29C	109.5
C7—C8—H8B	109.2	H29A—C29—H29C	109.5
C9—C8—H8B	109.2	H29B—C29—H29C	109.5
H8A—C8—H8B	107.9	C31—C30—C16	117.0 (3)
O4—C9—O5	110.2 (3)	C31—C30—H30A	108.0
O4—C9—C8	110.0 (3)	C16—C30—H30A	108.0
O5—C9—C8	106.6 (3)	C31—C30—H30B	108.0
O4—C9—C10	107.6 (3)	C16—C30—H30B	108.0
O5—C9—C10	106.2 (3)	H30A—C30—H30B	107.3
C8—C9—C10	116.2 (3)	C30—C31—H31A	109.5
C9—O4—C5	113.6 (2)	C30—C31—H31B	109.5
C11—C10—C9	107.0 (3)	H31A—C31—H31B	109.5
C11—C10—H10A	110.3	C30—C31—H31C	109.5
C9—C10—H10A	110.3	H31A—C31—H31C	109.5
C11—C10—H10B	110.3	H31B—C31—H31C	109.5
C9—C10—H10B	110.3	C12—C32—H32A	109.5
H10A—C10—H10B	108.6	C12—C32—H32B	109.5
C10—C11—C12	105.9 (3)	H32A—C32—H32B	109.5
C10—C11—H11A	110.6	C12—C32—H32C	109.5
C12—C11—H11A	110.6	H32A—C32—H32C	109.5
C10—C11—H11B	110.6	H32B—C32—H32C	109.5
C12—C11—H11B	110.6	C6—C33—H33A	109.5
H11A—C11—H11B	108.7	C6—C33—H33B	109.5
O5—C12—C13	107.7 (3)	H33A—C33—H33B	109.5
O5—C12—C32	108.8 (3)	C6—C33—H33C	109.5
C13—C12—C32	112.6 (3)	H33A—C33—H33C	109.5
O5—C12—C11	104.6 (3)	H33B—C33—H33C	109.5
C13—C12—C11	110.3 (3)	C4—C34—H34A	109.5
C32—C12—C11	112.4 (3)	C4—C34—H34B	109.5
C9—O5—C12	111.5 (2)	H34A—C34—H34B	109.5
O6—C13—C14	103.8 (3)	C4—C34—H34C	109.5
O6—C13—C12	110.0 (3)	H34A—C34—H34C	109.5
C14—C13—C12	118.5 (3)	H34B—C34—H34C	109.5
O6—C13—H13	108.1	O2—C35—H35A	109.5
C14—C13—H13	108.1	O2—C35—H35B	109.5
C12—C13—H13	108.1	H35A—C35—H35B	109.5
C13—C14—C15	103.1 (3)	O2—C35—H35C	109.5
C13—C14—H14A	111.1	H35A—C35—H35C	109.5
C15—C14—H14A	111.1	H35B—C35—H35C	109.5
C13—C14—H14B	111.1	C2—C36—H36A	109.5
C15—C14—H14B	111.1	C2—C36—H36B	109.5
H14A—C14—H14B	109.1	H36A—C36—H36B	109.5
C14—C15—C16	104.8 (3)	C2—C36—H36C	109.5
C14—C15—H15A	110.8	H36A—C36—H36C	109.5
C16—C15—H15A	110.8	H36B—C36—H36C	109.5
C14—C15—H15B	110.8	N1—C37—C38	114.2 (3)
C16—C15—H15B	110.8	N1—C37—H37A	108.7

H15A—C15—H15B	108.9	C38—C37—H37A	108.7
O6—C16—C17	106.0 (2)	N1—C37—H37B	108.7
O6—C16—C15	105.5 (3)	C38—C37—H37B	108.7
C17—C16—C15	114.4 (3)	H37A—C37—H37B	107.6
O6—C16—C30	107.7 (2)	O10—C38—N2	123.1 (3)
C17—C16—C30	108.3 (3)	O10—C38—C37	122.8 (3)
C15—C16—C30	114.4 (3)	N2—C38—C37	114.0 (3)
C13—O6—C16	108.0 (2)	C38—N2—C39	122.7 (3)
O7—C17—C16	111.5 (2)	C38—N2—H2	124 (2)
O7—C17—C18	104.3 (2)	C39—N2—H2	112 (2)
C16—C17—C18	118.1 (3)	N2—C39—C40	110.1 (3)
O7—C17—H17	107.5	N2—C39—C44	110.5 (3)
C16—C17—H17	107.5	C40—C39—C44	111.0 (3)
C18—C17—H17	107.5	N2—C39—H39	108.4
C19—C18—C29	110.3 (3)	C40—C39—H39	108.4
C19—C18—C17	98.5 (2)	C44—C39—H39	108.4
C29—C18—C17	117.3 (3)	C39—C40—C41	110.9 (3)
C19—C18—H18	110.0	C39—C40—H40A	109.5
C29—C18—H18	110.0	C41—C40—H40A	109.5
C17—C18—H18	110.0	C39—C40—H40B	109.5
C20—C19—C18	102.5 (2)	C41—C40—H40B	109.5
C20—C19—H19A	111.3	H40A—C40—H40B	108.0
C18—C19—H19A	111.3	C42—C41—C40	112.0 (5)
C20—C19—H19B	111.3	C42—C41—H41A	109.2
C18—C19—H19B	111.3	C40—C41—H41A	109.2
H19A—C19—H19B	109.2	C42—C41—H41B	109.2
O7—C20—C19	105.2 (2)	C40—C41—H41B	109.2
O7—C20—C21	111.4 (2)	H41A—C41—H41B	107.9
C19—C20—C21	115.4 (3)	C41—C42—C43	110.8 (4)
O7—C20—H20	108.2	C41—C42—H42A	109.5
C19—C20—H20	108.2	C43—C42—H42A	109.5
C21—C20—H20	108.2	C41—C42—H42B	109.5
C17—O7—C20	109.3 (2)	C43—C42—H42B	109.5
O8—C21—C20	106.7 (2)	H42A—C42—H42B	108.1
O8—C21—C22	109.7 (2)	C42—C43—C44	111.3 (3)
C20—C21—C22	113.1 (2)	C42—C43—H43A	109.4
O8—C21—H21	109.1	C44—C43—H43A	109.4
C20—C21—H21	109.1	C42—C43—H43B	109.4
C22—C21—H21	109.1	C44—C43—H43B	109.4
C28—C22—C23	110.6 (3)	H43A—C43—H43B	108.0
C28—C22—C21	113.5 (3)	C39—C44—C43	110.9 (4)
C23—C22—C21	108.4 (2)	C39—C44—H44A	109.5
C28—C22—H22	108.1	C43—C44—H44A	109.5
C23—C22—H22	108.1	C39—C44—H44B	109.5
C21—C22—H22	108.1	C43—C44—H44B	109.5
C24—C23—C22	113.5 (3)	H44A—C44—H44B	108.0

O1—C1—C2—C36	−67.3 (5)	C30—C16—C17—O7	171.3 (3)
N1—C1—C2—C36	111.0 (4)	O6—C16—C17—C18	165.9 (2)
O1—C1—C2—C3	53.5 (5)	C15—C16—C17—C18	−78.4 (3)
N1—C1—C2—C3	−128.2 (3)	C30—C16—C17—C18	50.5 (4)
C1—C2—C3—O2	−117.1 (4)	O7—C17—C18—C19	40.5 (3)

C36—C2—C3—O2	0.7 (5)	C16—C17—C18—C19	164.9 (3)
C1—C2—C3—C4	123.1 (3)	O7—C17—C18—C29	−77.6 (3)
C36—C2—C3—C4	−119.0 (4)	C16—C17—C18—C29	46.8 (4)
C4—C3—O2—C35	−102.5 (5)	C29—C18—C19—C20	80.5 (3)
C2—C3—O2—C35	136.1 (4)	C17—C18—C19—C20	−42.9 (3)
O2—C3—C4—C34	−61.9 (4)	C18—C19—C20—O7	31.2 (3)
C2—C3—C4—C34	58.2 (4)	C18—C19—C20—C21	154.4 (3)
O2—C3—C4—C5	171.2 (3)	C16—C17—O7—C20	−151.3 (2)
C2—C3—C4—C5	−68.6 (4)	C18—C17—O7—C20	−22.8 (3)
C34—C4—C5—O4	48.1 (4)	C19—C20—O7—C17	−5.1 (3)
C3—C4—C5—O4	175.1 (3)	C21—C20—O7—C17	−130.8 (2)
C34—C4—C5—C6	171.4 (3)	O7—C20—C21—O8	55.3 (3)
C3—C4—C5—C6	−61.6 (4)	C19—C20—C21—O8	−64.5 (3)
O4—C5—C6—C7	−58.0 (3)	O7—C20—C21—C22	176.1 (2)
C4—C5—C6—C7	179.8 (3)	C19—C20—C21—C22	56.2 (3)
O4—C5—C6—C33	66.5 (4)	O8—C21—C22—C28	−179.3 (3)
C4—C5—C6—C33	−55.7 (4)	C20—C21—C22—C28	61.7 (4)
C33—C6—C7—O3	162.6 (3)	O8—C21—C22—C23	−56.0 (3)
C5—C6—C7—O3	−71.7 (3)	C20—C21—C22—C23	−175.0 (2)
C33—C6—C7—C8	−72.0 (4)	C28—C22—C23—C24	176.8 (3)
C5—C6—C7—C8	53.6 (3)	C21—C22—C23—C24	51.8 (3)
O3—C7—C8—C9	72.4 (4)	C22—C23—C24—C25	−50.2 (4)
C6—C7—C8—C9	−51.6 (4)	C22—C23—C24—C27	−174.4 (3)
C7—C8—C9—O4	52.7 (3)	C23—C24—C25—O9	−69.1 (3)
C7—C8—C9—O5	−66.8 (4)	C27—C24—C25—O9	53.9 (3)
C7—C8—C9—C10	175.2 (3)	C23—C24—C25—O8	51.3 (3)
O5—C9—O4—C5	58.0 (3)	C27—C24—C25—O8	174.2 (3)
C8—C9—O4—C5	−59.2 (3)	C23—C24—C25—C26	167.4 (3)
C10—C9—O4—C5	173.4 (3)	C27—C24—C25—C26	−69.7 (3)
C6—C5—O4—C9	63.0 (3)	C20—C21—O8—C25	−173.5 (2)
C4—C5—O4—C9	−169.5 (3)	C22—C21—O8—C25	63.6 (3)
O4—C9—C10—C11	−115.0 (3)	O9—C25—O8—C21	57.9 (3)
O5—C9—C10—C11	3.0 (4)	C26—C25—O8—C21	179.3 (2)
C8—C9—C10—C11	121.2 (4)	C24—C25—O8—C21	−60.4 (3)
C9—C10—C11—C12	10.5 (4)	O9—C25—C26—N1	46.4 (4)
C10—C11—C12—O5	−19.9 (4)	O8—C25—C26—N1	−74.1 (3)
C10—C11—C12—C13	−135.4 (3)	C24—C25—C26—N1	167.2 (3)
C10—C11—C12—C32	98.0 (4)	O1—C1—N1—C37	179.3 (3)
O4—C9—O5—C12	99.7 (3)	C2—C1—N1—C37	1.0 (5)
C8—C9—O5—C12	−141.0 (3)	O1—C1—N1—C26	10.5 (5)
C10—C9—O5—C12	−16.6 (4)	C2—C1—N1—C26	−167.8 (3)
C13—C12—O5—C9	140.3 (3)	C25—C26—N1—C1	−114.2 (3)
C32—C12—O5—C9	−97.4 (3)	C25—C26—N1—C37	76.0 (3)
C11—C12—O5—C9	23.0 (4)	O6—C16—C30—C31	56.2 (4)
O5—C12—C13—O6	66.0 (3)	C17—C16—C30—C31	170.4 (3)
C32—C12—C13—O6	−54.0 (4)	C15—C16—C30—C31	−60.7 (4)
C11—C12—C13—O6	179.5 (3)	C1—N1—C37—C38	−77.4 (4)
O5—C12—C13—C14	−53.1 (4)	C26—N1—C37—C38	91.7 (3)
C32—C12—C13—C14	−173.1 (3)	N1—C37—C38—O10	−6.3 (5)
C11—C12—C13—C14	60.4 (4)	N1—C37—C38—N2	173.1 (3)
O6—C13—C14—C15	38.5 (3)	O10—C38—N2—C39	10.6 (5)
C12—C13—C14—C15	160.8 (3)	C37—C38—N2—C39	−168.8 (3)

C13—C14—C15—C16	−27.5 (4)	C38—N2—C39—C40	71.7 (4)
C14—C15—C16—O6	7.2 (3)	C38—N2—C39—C44	−165.4 (3)
C14—C15—C16—C17	−108.8 (3)	N2—C39—C40—C41	178.0 (4)
C14—C15—C16—C30	125.4 (3)	C44—C39—C40—C41	55.3 (5)
C14—C13—O6—C16	−35.1 (3)	C39—C40—C41—C42	−56.1 (6)
C12—C13—O6—C16	−162.8 (2)	C40—C41—C42—C43	56.0 (6)
C17—C16—O6—C13	139.0 (2)	C41—C42—C43—C44	−55.5 (6)
C15—C16—O6—C13	17.3 (3)	N2—C39—C44—C43	−177.7 (3)
C30—C16—O6—C13	−105.3 (3)	C40—C39—C44—C43	−55.2 (4)
O6—C16—C17—O7	−73.3 (3)	C42—C43—C44—C39	55.3 (6)
C15—C16—C17—O7	42.4 (3)		

Hydrogen-bond geometry (Å, º)

D—H···*A*	*D*—H	H···*A*	*D*···*A*	*D*—H···*A*
O3—H3···O5	0.82	2.12	2.787 (3)	139
C14—H14*B*···O1^{i}	0.97	2.53	3.410 (5)	152
O9—H91···O3	0.82	2.06	2.870 (3)	169
C37—H37*A*···O8	0.97	2.23	2.986 (4)	134
N2—H2···O6	0.85 (3)	2.55 (3)	3.385 (4)	168 (3)

Symmetry code: (i) *x*, *y*+1, *z*.
