# Supplementary material for: Access to Cyclic Monensin Derivatives via a Four-Component Ugi Reaction
Source: J Org Chem. 2026 Jul 4;91(28):9933–9. doi: 10.1021/acs.joc.6c01246 (PMC13386529; doi:10.1021/acs.joc.6c01246)

checkCIF/PLATON report

Structure factors have been supplied for datablock(s) RG_MONUGI2_100

THIS REPORT IS FOR GUIDANCE ONLY. IF USED AS PART OF A REVIEW PROCEDURE FOR PUBLICATION, IT SHOULD NOT REPLACE THE EXPERTISE OF AN EXPERIENCED CRYSTALLOGRAPHIC REFEREE.

No syntax errors found. CIF dictionary Interpreting this report

Datablock: RG_MONUGI2_100

Bond precision:	C-C = 0.0045 A		Wavelength=0.71073
Cell:	a=12.5180(3)	b=11.3789(3)	c=15.0822(4)
	alpha=90	beta=96.558(2)	gamma=90
Temperature:	100 K		

	Calculated	Reported
Volume	2134.27(10)	2134.27(10)
Space group	P 21	P 21
Hall group	P 2yb	P 2yb
Moiety formula	C45 H70 N2 O10	C45 H70 N2 O10
Sum formula	C45 H70 N2 O10	C45 H70 N2 O10
Mr	799.03	799.03
Dx,g cm-3	1.243	1.243
Z	2	2
Mu (mm-1)	0.087	0.087
F000	868.0	868.0
F000'	868.42	
h,k,lmax	17,15,20	17,15,20
Nref	11708[6134]	10554
Tmin,Tmax	0.970,0.979	0.988,1.000
Tmin'	0.970	

Correction method= # Reported T Limits: Tmin=0.988 Tmax=1.000
AbsCorr = MULTI-SCAN

Data completeness= 1.72/0.90   Theta(max)= 29.315

R(reflections)= 0.0562(7646)   wR2(reflections)= 0.1098(10554)

S = 1.008   Npar= 525

The following ALERTS were generated. Each ALERT has the format
test-name_ALERT_alert-type_alert-level.
Click on the hyperlinks for more details of the test.

Alert level B

```
PLAT410_ALERT_2_B Short Intra H...H Contact  H2        ..H37B      .        1.86 Ang.
                                                      x,y,z  =      1_555 Check
```

Alert level C

```
STRVA01_ALERT_4_C           Flack test results are ambiguous.
           From the CIF: _refine_ls_abs_structure_Flack    0.500
           From the CIF: _refine_ls_abs_structure_Flack_su    0.500
PLAT340_ALERT_3_C Low Bond Precision on  C-C Bonds ...............    0.00455 Ang.
PLAT420_ALERT_2_C D-H Bond Without Acceptor  N2       --H2A       .     Please Check
PLAT910_ALERT_3_C Missing FCF Reflection(s) Below Theta(Min)[Deg]=       2.43 Note
                1  0  0,  -1  0  1,   0  0  1,   0  1  1,   1  0  1,
```

Alert level G

```
PLAT007_ALERT_5_G Number of Unrefined Donor-H Atoms .............          3 Report
              H2A     H3A     H9
PLAT032_ALERT_4_G Std. Uncertainty on Flack Parameter Value High .      0.500 Report
PLAT398_ALERT_2_G Deviating  C-O-C   Angle From 120 for O6       .      108.9 Degree
PLAT398_ALERT_2_G Deviating  C-O-C   Angle From 120 for O7       .      108.6 Degree
PLAT791_ALERT_4_G Model has Chirality at C2         (Sohncke SpGr)          S Verify
PLAT791_ALERT_4_G Model has Chirality at C3         (Sohncke SpGr)          R Verify
PLAT791_ALERT_4_G Model has Chirality at C4         (Sohncke SpGr)          S Verify
PLAT791_ALERT_4_G Model has Chirality at C5         (Sohncke SpGr)          S Verify
PLAT791_ALERT_4_G Model has Chirality at C6         (Sohncke SpGr)          R Verify
PLAT791_ALERT_4_G Model has Chirality at C7         (Sohncke SpGr)          S Verify
PLAT791_ALERT_4_G Model has Chirality at C9         (Sohncke SpGr)          R Verify
PLAT791_ALERT_4_G Model has Chirality at C12        (Sohncke SpGr)          S Verify
PLAT791_ALERT_4_G Model has Chirality at C13        (Sohncke SpGr)          R Verify
PLAT791_ALERT_4_G Model has Chirality at C16        (Sohncke SpGr)          S Verify
PLAT791_ALERT_4_G Model has Chirality at C17        (Sohncke SpGr)          R Verify
PLAT791_ALERT_4_G Model has Chirality at C18        (Sohncke SpGr)          S Verify
PLAT791_ALERT_4_G Model has Chirality at C20        (Sohncke SpGr)          R Verify
PLAT791_ALERT_4_G Model has Chirality at C21        (Sohncke SpGr)          S Verify
PLAT791_ALERT_4_G Model has Chirality at C22        (Sohncke SpGr)          S Verify
PLAT791_ALERT_4_G Model has Chirality at C24        (Sohncke SpGr)          R Verify
PLAT791_ALERT_4_G Model has Chirality at C25        (Sohncke SpGr)          R Verify
PLAT899_ALERT_4_G SHELXL2018 is Outdated   and Succeeded by SHELXL     2019/3 Note
PLAT912_ALERT_4_G Missing # of FCF Reflections Above STh/L=  0.600       356 Note
PLAT916_ALERT_2_G Hooft y and Flack x Parameter Values Differ by .      0.10 Check
PLAT965_ALERT_2_G The SHELXL WEIGHT Optimisation has not Converged     Please Check
PLAT969_ALERT_5_G The 'Henn et al.' R-Factor-gap value ..........      2.077 Note
```

```
              Predicted wR2: Based on SigI**2  5.29 or SHELX Weight 10.89 Note
PLAT978_ALERT_2_G Number C-C Bonds with Positive Residual Density.         3 Info
```

```
  0 ALERT level A = Most likely a serious problem - resolve or explain
  1 ALERT level B = A potentially serious problem, consider carefully
  4 ALERT level C = Check. Ensure it is not caused by an omission or oversight
 27 ALERT level G = General information/check it is not something unexpected

  0 ALERT type 1 CIF construction/syntax error, inconsistent or missing data
  7 ALERT type 2 Indicator that the structure model may be wrong or deficient
  2 ALERT type 3 Indicator that the structure quality may be low
 21 ALERT type 4 Improvement, methodology, query or suggestion
  2 ALERT type 5 Informative message, check
```

It is advisable to attempt to resolve as many as possible of the alerts in all categories. Often the minor alerts point to easily fixed oversights, errors and omissions in your CIF or refinement strategy, so attention to these fine details can be worthwhile. It is up to the individual to critically assess their own results and, if necessary, seek expert advice.

PLATON version of 23/04/2026; check.def file version of 30/03/2026

---

duplicate check

No duplication found

Datablock RG_MONUGI2_100 - ellipsoid plot

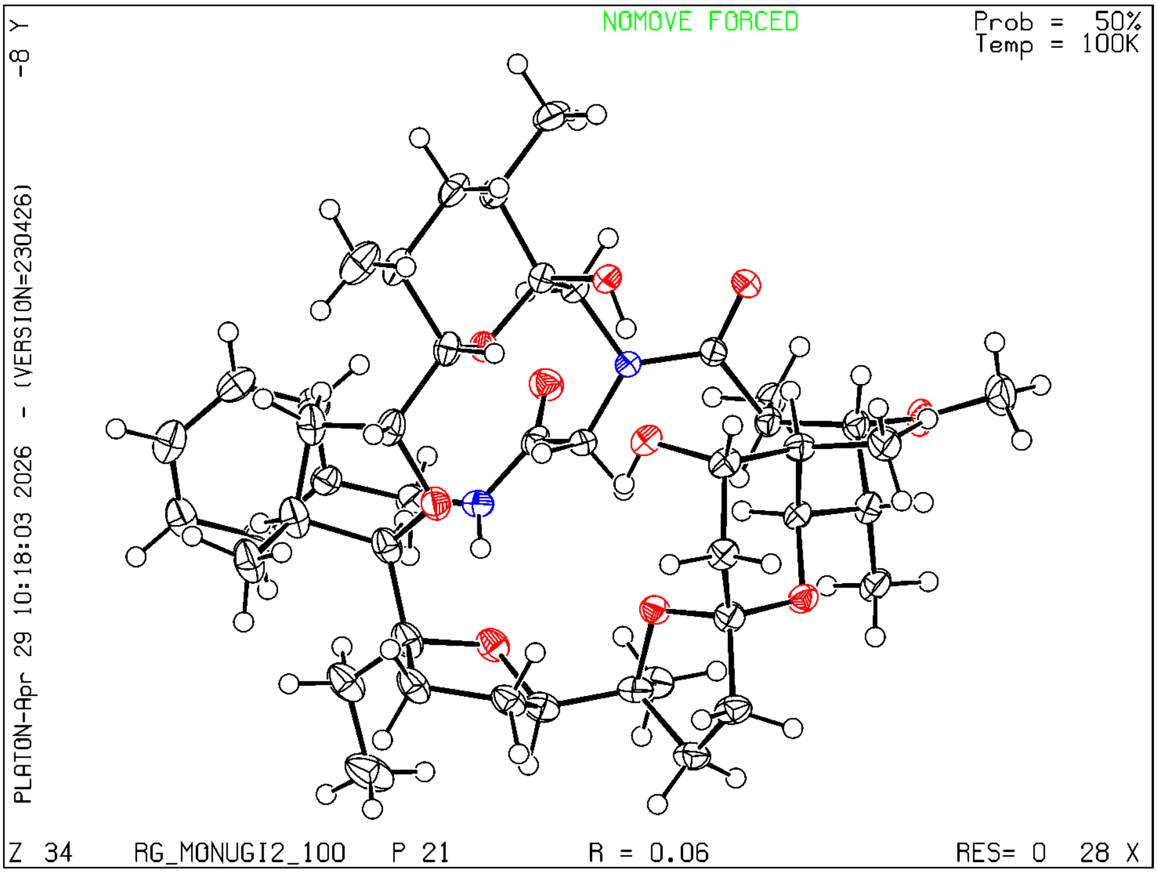

Supplement: Supplementary file 1 [file jo6c01246_si_001.zip › Compouds data/Compound 6/scXRD/checkcif_UGI_2_6.pdf]
