# Supplementary material for: Access to Cyclic Monensin Derivatives via a Four-Component Ugi Reaction
Source: J Org Chem. 2026 Jul 4;91(28):9933–9. doi: 10.1021/acs.joc.6c01246 (PMC13386529; doi:10.1021/acs.joc.6c01246)
Supplement: Supplementary file 1 [file jo6c01246_si_001.zip › Compouds data/Compound 6/scXRD/printcif_RG_MONUGI2_100.pdf]

Title

Enter author details here

Abstract

Table 1

Experimental details

Crystal data	
Chemical formula	$C_{45}H_{70}N_2O_{10}$
M_r	799.03
Crystal system, space group	Monoclinic, $P2_1$
Temperature (K)	100
a, b, c (Å)	12.5180 (3), 11.3789 (3), 15.0822 (4)
β (°)	96.558 (2)
V (Å^3)	2134.27 (10)
Z	2
Radiation type	Mo $K\alpha$
μ (mm^{-1})	0.09
Crystal size (mm)	0.35 × 0.29 × 0.24
Data collection	
Diffractometer	Xcalibur, Atlas
Absorption correction	Multi-scan<br>*CrysAlis PRO* 1.171.42.93a (Rigaku Oxford Diffraction, 2023) Empirical absorption correction using spherical harmonics, implemented in SCALE3 ABSPACK scaling algorithm.
T_{min}, T_{max}	0.988, 1.000
No. of measured, independent and observed [$I > 2\sigma(I)$] reflections	42363, 10554, 7646
R_{int}	0.070
(sin $\theta/\lambda)_{max}$ (Å^{-1})	0.689
Refinement	
$R[F^2 > 2\sigma(F^2)]$, $wR(F^2)$, S	0.056, 0.110, 1.01
No. of reflections	10554
No. of parameters	525
No. of restraints	1
H-atom treatment	H-atom parameters constrained
$\Delta\rho_{max}$, $\Delta\rho_{min}$ (e Å^{-3})	0.22, −0.25
Absolute structure	Flack x determined using 2682 quotients [(I+)-(I-)]/[(I+)+(I-)] (Parsons, Flack and Wagner, Acta Cryst. B69 (2013) 249-259).
Absolute structure parameter	0.5 (5)

Computer programs: *CrysAlis PRO* 1.171.42.93a (Rigaku Oxford Diffraction, 2023), *SHELXT* 2014/5 (Sheldrick, 2014), *SHELXL2018*/3 (Sheldrick, 2018), Brandenburg & Putz (2006). Diamond 3.0. Crystal and Molecular Structure Visualisation, University of Bonn, Germany.

Table 2

Hydrogen-bond geometry (Å, º)

D—H···*A*	*D*—H	H···*A*	*D*···*A*	*D*—H···*A*
O3—H3*A*···O5	0.84	2.10	2.798 (3)	140
O9—H9···O3	0.84	1.88	2.697 (3)	164

Acknowledgements

Funding information

References

Figure 1

supporting information

Title

Computing details

Data collection: *CrysAlis PRO* 1.171.42.93a (Rigaku Oxford Diffraction, 2023); cell refinement: *CrysAlis PRO* 1.171.42.93a (Rigaku Oxford Diffraction, 2023); data reduction: *CrysAlis PRO* 1.171.42.93a (Rigaku Oxford Diffraction, 2023); program(s) used to solve structure: *SHELXT* 2014/5 (Sheldrick, 2014); program(s) used to refine structure: *SHELXL2018*/3 (Sheldrick, 2018); molecular graphics: Brandenburg & Putz (2006). Diamond 3.0. Crystal and Molecular Structure Visualisation, University of Bonn, Germany; software used to prepare material for publication: *SHELXL2018*/3 (Sheldrick, 2018).

(RG_MONUGI2_100)

Crystal data

$C_{45}H_{70}N_2O_{10}$
M_r = 799.03
Monoclinic, $P2_1$
a = 12.5180 (3) Å
b = 11.3789 (3) Å
c = 15.0822 (4) Å
β = 96.558 (2)°
V = 2134.27 (10) Å^3
Z = 2
F(000) = 868
D_x = 1.243 Mg m^{-3}
Mo $K\alpha$ radiation, λ = 0.71073 Å
Cell parameters from 11452 reflections
θ = 2.9–26.3°
μ = 0.09 mm^{-1}
T = 100 K
Parallelepiped, colourless
0.35 × 0.29 × 0.24 mm

Data collection

Xcalibur, Atlas diffractometer
Radiation source: fine-focus sealed X-ray tube
Detector resolution: 10.6249 pixels mm^{-1}
ω–scan
Absorption correction: multi-scan *CrysAlis PRO* 1.171.42.93a (Rigaku Oxford Diffraction, 2023) Empirical absorption correction using spherical harmonics, implemented in SCALE3 ABSPACK scaling algorithm.
T_{min} = 0.988, T_{max} = 1.000
42363 measured reflections
10554 independent reflections
7646 reflections with $I > 2\sigma(I)$
R_{int} = 0.070
θ_{max} = 29.3°, θ_{min} = 2.4°
h = −17→16
k = −15→15
l = −20→20

Refinement

Refinement on F^2
Least-squares matrix: full
$R[F^2 > 2\sigma(F^2)]$ = 0.056
$wR(F^2)$ = 0.110
S = 1.01
10554 reflections
525 parameters
1 restraint
Primary atom site location: structure-invariant direct methods
Secondary atom site location: difference Fourier map
Hydrogen site location: inferred from neighbouring sites
H-atom parameters constrained
$w = 1/[\sigma^2(F_o^2) + (0.0427P)^2 + 0.2201P]$ where $P = (F_o^2 + 2F_c^2)/3$
$(\Delta/\sigma)_{max}$ < 0.001
$\Delta\rho_{max}$ = 0.22 e Å^{-3}
$\Delta\rho_{min}$ = −0.25 e Å^{-3}
Absolute structure: Flack x determined using 2682 quotients [(I+)-(I-)]/[(I+)+(I-)] (Parsons, Flack and Wagner, Acta Cryst. B69 (2013) 249-259).
Absolute structure parameter: 0.5 (5)

Special details

Geometry. All e.s.d.'s (except the e.s.d. in the dihedral angle between two l.s. planes) are estimated using the full covariance matrix. The cell e.s.d.'s are taken into account individually in the estimation of e.s.d.'s in distances, angles and torsion angles; correlations between e.s.d.'s in cell parameters are only used when they are defined by crystal symmetry. An approximate (isotropic) treatment of cell e.s.d.'s is used for estimating e.s.d.'s involving l.s. planes.

Refinement. Refinement of F^2 against ALL reflections. The weighted R-factor wR and goodness of fit S are based on F^2, conventional R-factors R are based on F, with F set to zero for negative F^2. The threshold expression of $F^2 > \sigma(F^2)$ is used only for calculating R-factors(gt) *etc*. and is not relevant to the choice of reflections for refinement. R-factors based on F^2 are statistically about twice as large as those based on F, and R- factors based on ALL data will be even larger.

Fractional atomic coordinates and isotropic or equivalent isotropic displacement parameters (Å^2)

	x	y	z	U_{iso}*/U_{eq}
N1	0.37598 (18)	0.6961 (2)	0.73130 (17)	0.0199 (6)
C1	0.4609 (2)	0.7665 (3)	0.7610 (2)	0.0201 (7)
O1	0.45112 (15)	0.8475 (2)	0.81305 (14)	0.0257 (5)
C2	0.5681 (2)	0.7514 (3)	0.7216 (2)	0.0225 (7)
H2	0.572562	0.669018	0.699162	0.027*
C3	0.6634 (2)	0.7727 (3)	0.7956 (2)	0.0229 (7)
H3	0.634008	0.803399	0.850063	0.027*
O2	0.73184 (16)	0.8609 (2)	0.76375 (15)	0.0296 (5)
C4	0.7275 (2)	0.6588 (3)	0.8208 (2)	0.0230 (7)
H4	0.782043	0.677710	0.872545	0.028*
C5	0.6521 (2)	0.5653 (3)	0.8519 (2)	0.0215 (7)
H5	0.590496	0.555322	0.804145	0.026*
C6	0.6062 (2)	0.5939 (3)	0.9394 (2)	0.0246 (7)
H6	0.560885	0.666041	0.929156	0.029*
C7	0.5319 (2)	0.4926 (3)	0.9616 (2)	0.0241 (7)
H7	0.513054	0.504979	1.023619	0.029*
O3	0.43423 (16)	0.4948 (2)	0.90173 (15)	0.0270 (5)
H3A	0.439639	0.448403	0.859129	0.041*
C8	0.5878 (2)	0.3748 (3)	0.9588 (2)	0.0244 (7)
H8A	0.534765	0.311239	0.963719	0.029*
H8B	0.643713	0.368473	1.010530	0.029*
C9	0.6394 (2)	0.3585 (3)	0.8735 (2)	0.0242 (7)
O4	0.71020 (16)	0.45519 (19)	0.86143 (14)	0.0233 (5)
C10	0.7028 (3)	0.2457 (3)	0.8676 (2)	0.0284 (8)
H10A	0.671988	0.181541	0.900975	0.034*
H10B	0.779046	0.257079	0.891672	0.034*
C11	0.6924 (3)	0.2187 (3)	0.7680 (2)	0.0292 (8)
H11A	0.685135	0.133076	0.757504	0.035*
H11B	0.756332	0.247205	0.741360	0.035*
C12	0.5898 (2)	0.2841 (3)	0.7268 (2)	0.0254 (7)
O5	0.55682 (15)	0.3532 (2)	0.80026 (13)	0.0225 (5)
C13	0.4991 (3)	0.1992 (3)	0.6975 (2)	0.0272 (8)
H13	0.523565	0.143956	0.652352	0.033*
C14	0.4540 (3)	0.1286 (3)	0.7694 (2)	0.0279 (8)
H14A	0.449844	0.176271	0.823834	0.034*
H14B	0.497992	0.057694	0.785205	0.034*
C15	0.3419 (3)	0.0956 (3)	0.7254 (2)	0.0299 (8)
H15A	0.290283	0.090478	0.770318	0.036*
H15B	0.343454	0.019085	0.694264	0.036*
C16	0.3103 (3)	0.1954 (3)	0.6583 (2)	0.0282 (8)

O6	0.40806 (17)	0.26563 (19)	0.65616 (14)	0.0265 (5)
C17	0.2260 (2)	0.2807 (3)	0.6861 (2)	0.0266 (7)
H17	0.231503	0.353874	0.650127	0.032*
C18	0.1067 (3)	0.2444 (3)	0.6742 (2)	0.0319 (8)
H18	0.076222	0.251580	0.610225	0.038*
C19	0.0632 (3)	0.3413 (3)	0.7294 (2)	0.0316 (8)
H19A	−0.007447	0.319338	0.748022	0.038*
H19B	0.055645	0.415970	0.695583	0.038*
C20	0.1488 (2)	0.3515 (3)	0.8100 (2)	0.0265 (7)
H20	0.130683	0.294437	0.856432	0.032*
O7	0.24811 (16)	0.31404 (19)	0.77833 (14)	0.0264 (5)
C21	0.1590 (2)	0.4735 (3)	0.8522 (2)	0.0230 (7)
H21	0.210323	0.469791	0.908065	0.028*
C22	0.0501 (2)	0.5198 (3)	0.8749 (2)	0.0279 (8)
H22	−0.001020	0.519059	0.818898	0.034*
C23	0.0624 (2)	0.6468 (3)	0.9068 (2)	0.0276 (8)
H23A	−0.009275	0.678465	0.915620	0.033*
H23B	0.107460	0.648558	0.965185	0.033*
C24	0.1133 (2)	0.7250 (3)	0.8413 (2)	0.0259 (7)
H24	0.064416	0.725770	0.784021	0.031*
C25	0.2205 (2)	0.6691 (3)	0.8226 (2)	0.0223 (7)
O8	0.20127 (16)	0.55142 (18)	0.78975 (14)	0.0220 (5)
O9	0.28862 (15)	0.6693 (2)	0.90255 (14)	0.0241 (5)
H9	0.341211	0.624832	0.897804	0.036*
C26	0.2686 (2)	0.7349 (3)	0.7486 (2)	0.0220 (7)
H26A	0.219209	0.726691	0.692834	0.026*
H26B	0.272247	0.819431	0.764157	0.026*
C27	0.1257 (3)	0.8511 (3)	0.8746 (2)	0.0325 (8)
H27A	0.170940	0.852449	0.932117	0.049*
H27B	0.054793	0.883650	0.881955	0.049*
H27C	0.159494	0.898474	0.831213	0.049*
C28	0.0023 (3)	0.4443 (4)	0.9436 (3)	0.0403 (10)
H28A	−0.010172	0.364674	0.919882	0.060*
H28B	−0.066086	0.478445	0.956599	0.060*
H28C	0.052287	0.441121	0.998466	0.060*
C29	0.0814 (3)	0.1225 (3)	0.7107 (3)	0.0388 (9)
H29A	0.108621	0.118065	0.774213	0.058*
H29B	0.116027	0.061784	0.677866	0.058*
H29C	0.003426	0.110039	0.703427	0.058*
C30	0.2746 (3)	0.1526 (3)	0.5637 (2)	0.0351 (9)
H30A	0.212253	0.099566	0.565596	0.042*
H30B	0.249854	0.221285	0.526620	0.042*
C31	0.3601 (4)	0.0882 (4)	0.5182 (3)	0.0470 (11)
H31A	0.329600	0.063412	0.458431	0.070*
H31B	0.384470	0.018955	0.553559	0.070*
H31C	0.421189	0.140760	0.513483	0.070*
C32	0.6130 (3)	0.3641 (3)	0.6505 (2)	0.0293 (8)
H32A	0.548298	0.409461	0.629910	0.044*
H32B	0.633859	0.316433	0.601202	0.044*
H32C	0.671610	0.418001	0.671248	0.044*
C33	0.6930 (3)	0.6196 (3)	1.0169 (2)	0.0311 (8)
H33A	0.660475	0.620879	1.073033	0.047*

H33B	0.725917	0.696064	1.007616	0.047*
H33C	0.748233	0.558201	1.019817	0.047*
C34	0.7886 (2)	0.6181 (3)	0.7442 (2)	0.0275 (8)
H34A	0.738905	0.612836	0.689242	0.041*
H34B	0.820520	0.540766	0.758407	0.041*
H34C	0.845684	0.674668	0.735842	0.041*
C35	0.8018 (3)	0.9128 (3)	0.8333 (3)	0.0418 (10)
H35A	0.841654	0.977108	0.808980	0.063*
H35B	0.852416	0.853587	0.859954	0.063*
H35C	0.759569	0.943734	0.878941	0.063*
C36	0.5625 (2)	0.8360 (3)	0.6417 (2)	0.0279 (8)
H36A	0.498058	0.818851	0.600503	0.042*
H36B	0.626558	0.825955	0.610798	0.042*
H36C	0.559167	0.917144	0.663044	0.042*
C37	0.3788 (2)	0.5963 (3)	0.6718 (2)	0.0224 (7)
H37A	0.338787	0.530249	0.695107	0.027*
H37B	0.454369	0.570981	0.671089	0.027*
C38	0.3306 (2)	0.6234 (3)	0.5770 (2)	0.0233 (7)
O10	0.30917 (17)	0.7235 (2)	0.55092 (15)	0.0281 (5)
N2	0.3103 (2)	0.5274 (2)	0.52471 (18)	0.0253 (6)
H2A	0.340254	0.459992	0.541927	0.030*
C39	0.2403 (2)	0.5328 (3)	0.4407 (2)	0.0284 (8)
H39A	0.274802	0.491222	0.393849	0.034*
H39B	0.229835	0.615868	0.422201	0.034*
C40	0.1318 (2)	0.4772 (3)	0.4496 (2)	0.0238 (7)
C41	0.0969 (3)	0.3798 (3)	0.4001 (2)	0.0294 (8)
H41	0.141255	0.346557	0.359564	0.035*
C42	−0.0026 (3)	0.3299 (3)	0.4090 (2)	0.0333 (8)
H42	−0.026143	0.263323	0.374081	0.040*
C43	−0.0673 (3)	0.3765 (3)	0.4680 (2)	0.0342 (9)
H43	−0.135179	0.341995	0.474239	0.041*
C44	−0.0328 (3)	0.4741 (3)	0.5183 (2)	0.0318 (8)
H44	−0.077060	0.506889	0.559119	0.038*
C45	0.0662 (3)	0.5237 (3)	0.5090 (2)	0.0283 (8)
H45	0.089643	0.590385	0.543830	0.034*

Atomic displacement parameters (Å^2)

	U^{11}	U^{22}	U^{33}	U^{12}	U^{13}	U^{23}
N1	0.0159 (12)	0.0179 (14)	0.0258 (15)	0.0003 (10)	0.0023 (11)	−0.0029 (12)
C1	0.0193 (15)	0.0164 (16)	0.0240 (17)	−0.0003 (12)	0.0006 (13)	0.0026 (14)
O1	0.0234 (11)	0.0188 (12)	0.0350 (13)	−0.0016 (10)	0.0037 (10)	−0.0056 (11)
C2	0.0181 (15)	0.0210 (18)	0.0284 (18)	−0.0019 (13)	0.0026 (14)	0.0003 (14)
C3	0.0190 (15)	0.0197 (17)	0.0303 (18)	−0.0035 (13)	0.0045 (14)	0.0023 (14)
O2	0.0213 (10)	0.0236 (12)	0.0433 (14)	−0.0069 (10)	0.0003 (10)	0.0049 (12)
C4	0.0189 (15)	0.0244 (18)	0.0253 (18)	−0.0009 (13)	0.0007 (14)	0.0035 (15)
C5	0.0178 (15)	0.0226 (17)	0.0234 (18)	0.0029 (13)	−0.0004 (13)	0.0009 (14)
C6	0.0236 (16)	0.0209 (17)	0.0290 (19)	0.0032 (13)	0.0025 (15)	0.0002 (15)
C7	0.0226 (15)	0.0286 (19)	0.0214 (17)	0.0029 (14)	0.0035 (14)	0.0022 (15)
O3	0.0201 (11)	0.0298 (14)	0.0312 (13)	0.0027 (9)	0.0032 (10)	−0.0014 (11)
C8	0.0240 (16)	0.0231 (18)	0.0264 (18)	0.0008 (13)	0.0040 (14)	0.0055 (15)
C9	0.0244 (15)	0.0200 (17)	0.0281 (18)	0.0009 (14)	0.0027 (14)	0.0044 (15)

O4	0.0201 (11)	0.0204 (12)	0.0294 (13)	0.0025 (9)	0.0022 (10)	0.0027 (10)
C10	0.0296 (17)	0.0212 (18)	0.035 (2)	0.0064 (14)	0.0069 (16)	0.0050 (16)
C11	0.0324 (18)	0.0207 (18)	0.035 (2)	0.0058 (14)	0.0065 (16)	−0.0011 (16)
C12	0.0291 (17)	0.0223 (18)	0.0259 (18)	0.0048 (14)	0.0080 (15)	−0.0017 (15)
O5	0.0236 (10)	0.0200 (11)	0.0239 (11)	0.0006 (9)	0.0030 (9)	−0.0006 (10)
C13	0.0341 (18)	0.0212 (18)	0.0263 (18)	0.0036 (14)	0.0035 (15)	0.0011 (15)
C14	0.0369 (18)	0.0177 (17)	0.0284 (19)	0.0012 (14)	0.0002 (15)	−0.0002 (15)
C15	0.0400 (19)	0.0186 (17)	0.030 (2)	−0.0051 (15)	−0.0021 (16)	0.0004 (15)
C16	0.0350 (19)	0.0205 (18)	0.0282 (19)	−0.0063 (14)	0.0001 (16)	0.0029 (15)
O6	0.0325 (12)	0.0205 (12)	0.0261 (12)	−0.0013 (10)	0.0015 (10)	0.0028 (10)
C17	0.0319 (17)	0.0200 (17)	0.0262 (18)	−0.0043 (14)	−0.0037 (15)	0.0034 (15)
C18	0.0347 (18)	0.0276 (19)	0.031 (2)	−0.0087 (15)	−0.0058 (16)	0.0034 (16)
C19	0.0285 (17)	0.031 (2)	0.0331 (19)	−0.0092 (16)	−0.0034 (15)	0.0047 (17)
C20	0.0204 (15)	0.0273 (18)	0.0317 (18)	−0.0048 (14)	0.0021 (14)	0.0068 (16)
O7	0.0253 (11)	0.0251 (13)	0.0277 (13)	−0.0020 (9)	−0.0012 (10)	−0.0011 (10)
C21	0.0196 (15)	0.0287 (19)	0.0208 (17)	−0.0040 (14)	0.0028 (13)	0.0031 (14)
C22	0.0162 (15)	0.040 (2)	0.0278 (19)	−0.0016 (14)	0.0020 (14)	0.0038 (16)
C23	0.0161 (15)	0.042 (2)	0.0247 (18)	0.0028 (14)	0.0038 (14)	−0.0010 (16)
C24	0.0184 (15)	0.035 (2)	0.0248 (18)	0.0012 (14)	0.0033 (14)	−0.0031 (16)
C25	0.0177 (14)	0.0244 (18)	0.0244 (17)	−0.0007 (13)	0.0009 (13)	−0.0013 (15)
O8	0.0207 (10)	0.0214 (12)	0.0245 (12)	−0.0029 (9)	0.0043 (10)	−0.0004 (10)
O9	0.0180 (10)	0.0263 (13)	0.0276 (13)	0.0027 (9)	0.0003 (10)	−0.0041 (10)
C26	0.0164 (14)	0.0233 (17)	0.0264 (17)	0.0020 (13)	0.0030 (13)	−0.0020 (14)
C27	0.0238 (16)	0.034 (2)	0.041 (2)	0.0075 (16)	0.0092 (15)	−0.0016 (18)
C28	0.0254 (18)	0.053 (3)	0.044 (2)	0.0026 (17)	0.0114 (17)	0.016 (2)
C29	0.040 (2)	0.030 (2)	0.044 (2)	−0.0133 (17)	−0.0047 (18)	−0.0007 (18)
C30	0.050 (2)	0.0228 (19)	0.030 (2)	−0.0040 (16)	−0.0034 (17)	0.0007 (16)
C31	0.073 (3)	0.035 (2)	0.030 (2)	0.012 (2)	−0.009 (2)	−0.0073 (18)
C32	0.0328 (17)	0.0255 (18)	0.0304 (18)	0.0018 (15)	0.0073 (15)	−0.0001 (16)
C33	0.0303 (18)	0.034 (2)	0.029 (2)	−0.0010 (16)	0.0031 (16)	−0.0049 (16)
C34	0.0184 (15)	0.0278 (19)	0.037 (2)	0.0011 (14)	0.0055 (15)	0.0048 (16)
C35	0.032 (2)	0.026 (2)	0.065 (3)	−0.0068 (16)	−0.0067 (19)	0.002 (2)
C36	0.0236 (16)	0.029 (2)	0.0319 (18)	−0.0041 (14)	0.0043 (14)	0.0043 (16)
C37	0.0187 (15)	0.0200 (17)	0.0288 (19)	0.0007 (12)	0.0044 (14)	−0.0032 (15)
C38	0.0144 (14)	0.0258 (18)	0.0312 (19)	−0.0014 (13)	0.0090 (14)	0.0002 (15)
O10	0.0304 (12)	0.0233 (13)	0.0307 (13)	0.0003 (10)	0.0040 (10)	0.0009 (11)
N2	0.0246 (14)	0.0230 (15)	0.0281 (16)	0.0040 (11)	0.0027 (12)	−0.0047 (13)
C39	0.0276 (17)	0.032 (2)	0.0254 (18)	0.0020 (15)	0.0014 (15)	−0.0052 (16)
C40	0.0233 (15)	0.0242 (18)	0.0230 (17)	0.0040 (14)	−0.0020 (14)	0.0012 (14)
C41	0.0308 (18)	0.030 (2)	0.0264 (18)	0.0046 (15)	−0.0014 (15)	0.0000 (16)
C42	0.0379 (19)	0.026 (2)	0.033 (2)	−0.0063 (16)	−0.0079 (17)	0.0034 (16)
C43	0.0261 (17)	0.039 (2)	0.036 (2)	−0.0035 (16)	−0.0007 (16)	0.0137 (18)
C44	0.0259 (17)	0.039 (2)	0.031 (2)	0.0086 (16)	0.0036 (15)	0.0081 (17)
C45	0.0279 (17)	0.0273 (19)	0.0290 (19)	0.0006 (14)	0.0000 (15)	−0.0026 (16)

Geometric parameters (Å, º)

N1—C1	1.366 (4)	C22—C28	1.520 (5)
N1—C37	1.450 (4)	C22—C23	1.524 (5)
N1—C26	1.467 (4)	C22—H22	1.0000
C1—O1	1.226 (4)	C23—C24	1.523 (5)
C1—C2	1.538 (4)	C23—H23A	0.9900

C2—C36	1.537 (4)	C23—H23B	0.9900
C2—C3	1.557 (4)	C24—C27	1.523 (5)
C2—H2	1.0000	C24—C25	1.539 (4)
C3—O2	1.437 (4)	C24—H24	1.0000
C3—C4	1.549 (4)	C25—O9	1.395 (4)
C3—H3	1.0000	C25—O8	1.439 (4)
O2—C35	1.416 (4)	C25—C26	1.524 (4)
C4—C34	1.528 (4)	O9—H9	0.8400
C4—C5	1.531 (4)	C26—H26A	0.9900
C4—H4	1.0000	C26—H26B	0.9900
C5—O4	1.448 (4)	C27—H27A	0.9800
C5—C6	1.534 (4)	C27—H27B	0.9800
C5—H5	1.0000	C27—H27C	0.9800
C6—C33	1.531 (5)	C28—H28A	0.9800
C6—C7	1.541 (4)	C28—H28B	0.9800
C6—H6	1.0000	C28—H28C	0.9800
C7—O3	1.436 (4)	C29—H29A	0.9800
C7—C8	1.515 (4)	C29—H29B	0.9800
C7—H7	1.0000	C29—H29C	0.9800
O3—H3A	0.8400	C30—C31	1.523 (5)
C8—C9	1.516 (4)	C30—H30A	0.9900
C8—H8A	0.9900	C30—H30B	0.9900
C8—H8B	0.9900	C31—H31A	0.9800
C9—O5	1.425 (4)	C31—H31B	0.9800
C9—O4	1.438 (4)	C31—H31C	0.9800
C9—C10	1.516 (4)	C32—H32A	0.9800
C10—C11	1.525 (5)	C32—H32B	0.9800
C10—H10A	0.9900	C32—H32C	0.9800
C10—H10B	0.9900	C33—H33A	0.9800
C11—C12	1.551 (4)	C33—H33B	0.9800
C11—H11A	0.9900	C33—H33C	0.9800
C11—H11B	0.9900	C34—H34A	0.9800
C12—O5	1.456 (4)	C34—H34B	0.9800
C12—C13	1.517 (5)	C34—H34C	0.9800
C12—C32	1.521 (5)	C35—H35A	0.9800
C13—O6	1.448 (4)	C35—H35B	0.9800
C13—C14	1.512 (5)	C35—H35C	0.9800
C13—H13	1.0000	C36—H36A	0.9800
C14—C15	1.530 (5)	C36—H36B	0.9800
C14—H14A	0.9900	C36—H36C	0.9800
C14—H14B	0.9900	C37—C38	1.519 (4)
C15—C16	1.543 (5)	C37—H37A	0.9900
C15—H15A	0.9900	C37—H37B	0.9900
C15—H15B	0.9900	C38—O10	1.225 (4)
C16—O6	1.465 (4)	C38—N2	1.354 (4)
C16—C30	1.524 (5)	N2—C39	1.457 (4)
C16—C17	1.528 (5)	N2—H2A	0.8800
C17—O7	1.439 (4)	C39—C40	1.518 (4)
C17—C18	1.540 (4)	C39—H39A	0.9900
C17—H17	1.0000	C39—H39B	0.9900
C18—C19	1.519 (5)	C40—C41	1.380 (5)
C18—C29	1.538 (5)	C40—C45	1.387 (5)

C18—H18	1.0000	C41—C42	1.389 (5)
C19—C20	1.531 (4)	C41—H41	0.9500
C19—H19A	0.9900	C42—C43	1.377 (5)
C19—H19B	0.9900	C42—H42	0.9500
C20—O7	1.446 (4)	C43—C44	1.386 (5)
C20—C21	1.527 (5)	C43—H43	0.9500
C20—H20	1.0000	C44—C45	1.384 (5)
C21—O8	1.438 (4)	C44—H44	0.9500
C21—C22	1.536 (4)	C45—H45	0.9500
C21—H21	1.0000		

C1—N1—C37	126.1 (2)	C20—C21—H21	109.2
C1—N1—C26	117.4 (2)	C22—C21—H21	109.2
C37—N1—C26	115.7 (2)	C28—C22—C23	110.8 (3)
O1—C1—N1	121.4 (3)	C28—C22—C21	112.7 (3)
O1—C1—C2	119.2 (3)	C23—C22—C21	109.6 (3)
N1—C1—C2	119.2 (3)	C28—C22—H22	107.9
C36—C2—C1	105.6 (2)	C23—C22—H22	107.9
C36—C2—C3	115.3 (3)	C21—C22—H22	107.9
C1—C2—C3	109.6 (3)	C24—C23—C22	112.6 (3)
C36—C2—H2	108.7	C24—C23—H23A	109.1
C1—C2—H2	108.7	C22—C23—H23A	109.1
C3—C2—H2	108.7	C24—C23—H23B	109.1
O2—C3—C4	110.7 (2)	C22—C23—H23B	109.1
O2—C3—C2	107.8 (2)	H23A—C23—H23B	107.8
C4—C3—C2	112.2 (3)	C23—C24—C27	111.8 (3)
O2—C3—H3	108.7	C23—C24—C25	108.4 (3)
C4—C3—H3	108.7	C27—C24—C25	113.0 (3)
C2—C3—H3	108.7	C23—C24—H24	107.8
C35—O2—C3	112.7 (3)	C27—C24—H24	107.8
C34—C4—C5	113.6 (3)	C25—C24—H24	107.8
C34—C4—C3	111.0 (3)	O9—C25—O8	111.2 (3)
C5—C4—C3	109.6 (2)	O9—C25—C26	112.2 (2)
C34—C4—H4	107.4	O8—C25—C26	105.6 (2)
C5—C4—H4	107.4	O9—C25—C24	107.5 (2)
C3—C4—H4	107.4	O8—C25—C24	109.4 (2)
O4—C5—C4	108.1 (2)	C26—C25—C24	110.9 (3)
O4—C5—C6	109.3 (3)	C21—O8—C25	114.1 (2)
C4—C5—C6	115.0 (3)	C25—O9—H9	109.5
O4—C5—H5	108.1	N1—C26—C25	115.5 (3)
C4—C5—H5	108.1	N1—C26—H26A	108.4
C6—C5—H5	108.1	C25—C26—H26A	108.4
C33—C6—C5	113.2 (3)	N1—C26—H26B	108.4
C33—C6—C7	111.7 (3)	C25—C26—H26B	108.4
C5—C6—C7	108.9 (3)	H26A—C26—H26B	107.5
C33—C6—H6	107.6	C24—C27—H27A	109.5
C5—C6—H6	107.6	C24—C27—H27B	109.5
C7—C6—H6	107.6	H27A—C27—H27B	109.5
O3—C7—C8	111.1 (3)	C24—C27—H27C	109.5
O3—C7—C6	110.0 (3)	H27A—C27—H27C	109.5
C8—C7—C6	111.3 (2)	H27B—C27—H27C	109.5
O3—C7—H7	108.1	C22—C28—H28A	109.5

C8—C7—H7	108.1	C22—C28—H28B	109.5
C6—C7—H7	108.1	H28A—C28—H28B	109.5
C7—O3—H3A	109.5	C22—C28—H28C	109.5
C7—C8—C9	112.1 (3)	H28A—C28—H28C	109.5
C7—C8—H8A	109.2	H28B—C28—H28C	109.5
C9—C8—H8A	109.2	C18—C29—H29A	109.5
C7—C8—H8B	109.2	C18—C29—H29B	109.5
C9—C8—H8B	109.2	H29A—C29—H29B	109.5
H8A—C8—H8B	107.9	C18—C29—H29C	109.5
O5—C9—O4	109.7 (2)	H29A—C29—H29C	109.5
O5—C9—C8	108.7 (2)	H29B—C29—H29C	109.5
O4—C9—C8	110.0 (3)	C31—C30—C16	115.4 (3)
O5—C9—C10	104.9 (3)	C31—C30—H30A	108.4
O4—C9—C10	107.8 (2)	C16—C30—H30A	108.4
C8—C9—C10	115.4 (3)	C31—C30—H30B	108.4
C9—O4—C5	111.4 (2)	C16—C30—H30B	108.4
C9—C10—C11	104.0 (3)	H30A—C30—H30B	107.5
C9—C10—H10A	111.0	C30—C31—H31A	109.5
C11—C10—H10A	111.0	C30—C31—H31B	109.5
C9—C10—H10B	111.0	H31A—C31—H31B	109.5
C11—C10—H10B	111.0	C30—C31—H31C	109.5
H10A—C10—H10B	109.0	H31A—C31—H31C	109.5
C10—C11—C12	105.7 (3)	H31B—C31—H31C	109.5
C10—C11—H11A	110.6	C12—C32—H32A	109.5
C12—C11—H11A	110.6	C12—C32—H32B	109.5
C10—C11—H11B	110.6	H32A—C32—H32B	109.5
C12—C11—H11B	110.6	C12—C32—H32C	109.5
H11A—C11—H11B	108.7	H32A—C32—H32C	109.5
O5—C12—C13	107.3 (2)	H32B—C32—H32C	109.5
O5—C12—C32	110.3 (3)	C6—C33—H33A	109.5
C13—C12—C32	111.1 (3)	C6—C33—H33B	109.5
O5—C12—C11	104.4 (3)	H33A—C33—H33B	109.5
C13—C12—C11	111.7 (3)	C6—C33—H33C	109.5
C32—C12—C11	111.7 (3)	H33A—C33—H33C	109.5
C9—O5—C12	111.6 (2)	H33B—C33—H33C	109.5
O6—C13—C14	104.2 (2)	C4—C34—H34A	109.5
O6—C13—C12	108.6 (3)	C4—C34—H34B	109.5
C14—C13—C12	117.2 (3)	H34A—C34—H34B	109.5
O6—C13—H13	108.8	C4—C34—H34C	109.5
C14—C13—H13	108.8	H34A—C34—H34C	109.5
C12—C13—H13	108.8	H34B—C34—H34C	109.5
C13—C14—C15	102.6 (3)	O2—C35—H35A	109.5
C13—C14—H14A	111.2	O2—C35—H35B	109.5
C15—C14—H14A	111.2	H35A—C35—H35B	109.5
C13—C14—H14B	111.2	O2—C35—H35C	109.5
C15—C14—H14B	111.2	H35A—C35—H35C	109.5
H14A—C14—H14B	109.2	H35B—C35—H35C	109.5
C14—C15—C16	105.0 (3)	C2—C36—H36A	109.5
C14—C15—H15A	110.8	C2—C36—H36B	109.5
C16—C15—H15A	110.8	H36A—C36—H36B	109.5
C14—C15—H15B	110.8	C2—C36—H36C	109.5
C16—C15—H15B	110.8	H36A—C36—H36C	109.5

H15A—C15—H15B	108.8	H36B—C36—H36C	109.5
O6—C16—C30	108.1 (3)	N1—C37—C38	112.9 (3)
O6—C16—C17	105.4 (2)	N1—C37—H37A	109.0
C30—C16—C17	108.8 (3)	C38—C37—H37A	109.0
O6—C16—C15	105.3 (3)	N1—C37—H37B	109.0
C30—C16—C15	113.8 (3)	C38—C37—H37B	109.0
C17—C16—C15	114.9 (3)	H37A—C37—H37B	107.8
C13—O6—C16	109.0 (2)	O10—C38—N2	123.0 (3)
O7—C17—C16	111.7 (3)	O10—C38—C37	122.6 (3)
O7—C17—C18	105.1 (3)	N2—C38—C37	114.4 (3)
C16—C17—C18	119.3 (3)	C38—N2—C39	121.8 (3)
O7—C17—H17	106.7	C38—N2—H2A	119.1
C16—C17—H17	106.7	C39—N2—H2A	119.1
C18—C17—H17	106.7	N2—C39—C40	111.2 (3)
C19—C18—C29	110.9 (3)	N2—C39—H39A	109.4
C19—C18—C17	98.4 (3)	C40—C39—H39A	109.4
C29—C18—C17	115.9 (3)	N2—C39—H39B	109.4
C19—C18—H18	110.4	C40—C39—H39B	109.4
C29—C18—H18	110.4	H39A—C39—H39B	108.0
C17—C18—H18	110.4	C41—C40—C45	118.8 (3)
C18—C19—C20	103.2 (3)	C41—C40—C39	121.2 (3)
C18—C19—H19A	111.1	C45—C40—C39	120.0 (3)
C20—C19—H19A	111.1	C40—C41—C42	120.5 (3)
C18—C19—H19B	111.1	C40—C41—H41	119.7
C20—C19—H19B	111.1	C42—C41—H41	119.7
H19A—C19—H19B	109.1	C43—C42—C41	120.4 (3)
O7—C20—C21	111.9 (2)	C43—C42—H42	119.8
O7—C20—C19	105.4 (3)	C41—C42—H42	119.8
C21—C20—C19	114.7 (3)	C42—C43—C44	119.5 (3)
O7—C20—H20	108.2	C42—C43—H43	120.2
C21—C20—H20	108.2	C44—C43—H43	120.2
C19—C20—H20	108.2	C45—C44—C43	119.9 (3)
C17—O7—C20	108.6 (2)	C45—C44—H44	120.1
O8—C21—C20	107.8 (2)	C43—C44—H44	120.1
O8—C21—C22	109.7 (3)	C44—C45—C40	120.9 (3)
C20—C21—C22	111.8 (3)	C44—C45—H45	119.6
O8—C21—H21	109.2	C40—C45—H45	119.6

C37—N1—C1—O1	−178.3 (3)	C17—C16—O6—C13	136.6 (3)
C26—N1—C1—O1	12.3 (4)	C15—C16—O6—C13	14.7 (3)
C37—N1—C1—C2	7.6 (4)	O6—C16—C17—O7	−71.1 (3)
C26—N1—C1—C2	−161.8 (3)	C30—C16—C17—O7	173.2 (3)
O1—C1—C2—C36	−83.2 (3)	C15—C16—C17—O7	44.3 (4)
N1—C1—C2—C36	91.0 (3)	O6—C16—C17—C18	165.9 (3)
O1—C1—C2—C3	41.5 (4)	C30—C16—C17—C18	50.2 (4)
N1—C1—C2—C3	−144.2 (3)	C15—C16—C17—C18	−78.7 (4)
C36—C2—C3—O2	−7.1 (4)	O7—C17—C18—C19	41.3 (3)
C1—C2—C3—O2	−126.1 (3)	C16—C17—C18—C19	167.5 (3)
C36—C2—C3—C4	−129.3 (3)	O7—C17—C18—C29	−76.8 (4)
C1—C2—C3—C4	111.7 (3)	C16—C17—C18—C29	49.3 (4)
C4—C3—O2—C35	−74.7 (3)	C29—C18—C19—C20	81.0 (3)
C2—C3—O2—C35	162.2 (3)	C17—C18—C19—C20	−41.0 (3)

O2—C3—C4—C34	−52.0 (4)	C18—C19—C20—O7	27.7 (3)
C2—C3—C4—C34	68.5 (3)	C18—C19—C20—C21	151.2 (3)
O2—C3—C4—C5	−178.4 (3)	C16—C17—O7—C20	−156.4 (3)
C2—C3—C4—C5	−57.8 (3)	C18—C17—O7—C20	−25.7 (3)
C34—C4—C5—O4	46.3 (3)	C21—C20—O7—C17	−126.4 (3)
C3—C4—C5—O4	171.2 (2)	C19—C20—O7—C17	−1.1 (3)
C34—C4—C5—C6	168.8 (3)	O7—C20—C21—O8	52.1 (3)
C3—C4—C5—C6	−66.3 (3)	C19—C20—C21—O8	−67.9 (3)
O4—C5—C6—C33	66.5 (3)	O7—C20—C21—C22	172.7 (2)
C4—C5—C6—C33	−55.3 (4)	C19—C20—C21—C22	52.7 (4)
O4—C5—C6—C7	−58.4 (3)	O8—C21—C22—C28	−177.8 (3)
C4—C5—C6—C7	179.7 (3)	C20—C21—C22—C28	62.6 (4)
C33—C6—C7—O3	162.0 (3)	O8—C21—C22—C23	−54.0 (3)
C5—C6—C7—O3	−72.1 (3)	C20—C21—C22—C23	−173.5 (3)
C33—C6—C7—C8	−74.3 (3)	C28—C22—C23—C24	178.2 (3)
C5—C6—C7—C8	51.5 (3)	C21—C22—C23—C24	53.3 (4)
O3—C7—C8—C9	73.0 (3)	C22—C23—C24—C27	−179.6 (3)
C6—C7—C8—C9	−49.9 (4)	C22—C23—C24—C25	−54.3 (3)
C7—C8—C9—O5	−66.0 (3)	C23—C24—C25—O9	−64.1 (3)
C7—C8—C9—O4	54.2 (3)	C27—C24—C25—O9	60.4 (3)
C7—C8—C9—C10	176.5 (3)	C23—C24—C25—O8	56.7 (3)
O5—C9—O4—C5	57.1 (3)	C27—C24—C25—O8	−178.8 (3)
C8—C9—O4—C5	−62.4 (3)	C23—C24—C25—C26	172.8 (3)
C10—C9—O4—C5	170.9 (3)	C27—C24—C25—C26	−62.6 (4)
C4—C5—O4—C9	−168.7 (2)	C20—C21—O8—C25	−177.0 (2)
C6—C5—O4—C9	65.5 (3)	C22—C21—O8—C25	61.1 (3)
O5—C9—C10—C11	30.6 (3)	O9—C25—O8—C21	55.9 (3)
O4—C9—C10—C11	−86.3 (3)	C26—C25—O8—C21	177.8 (2)
C8—C9—C10—C11	150.3 (3)	C24—C25—O8—C21	−62.7 (3)
C9—C10—C11—C12	−22.5 (3)	C1—N1—C26—C25	−105.1 (3)
C10—C11—C12—O5	6.6 (3)	C37—N1—C26—C25	84.5 (3)
C10—C11—C12—C13	−109.1 (3)	O9—C25—C26—N1	54.1 (4)
C10—C11—C12—C32	125.8 (3)	O8—C25—C26—N1	−67.2 (3)
O4—C9—O5—C12	87.4 (3)	C24—C25—C26—N1	174.3 (3)
C8—C9—O5—C12	−152.2 (3)	O6—C16—C30—C31	53.4 (4)
C10—C9—O5—C12	−28.2 (3)	C17—C16—C30—C31	167.3 (3)
C13—C12—O5—C9	132.1 (3)	C15—C16—C30—C31	−63.2 (4)
C32—C12—O5—C9	−106.6 (3)	C1—N1—C37—C38	−103.3 (3)
C11—C12—O5—C9	13.5 (3)	C26—N1—C37—C38	66.2 (3)
O5—C12—C13—O6	68.7 (3)	N1—C37—C38—O10	11.7 (4)
C32—C12—C13—O6	−52.0 (3)	N1—C37—C38—N2	−166.3 (2)
C11—C12—C13—O6	−177.4 (2)	O10—C38—N2—C39	−13.9 (4)
O5—C12—C13—C14	−49.0 (4)	C37—C38—N2—C39	164.0 (2)
C32—C12—C13—C14	−169.7 (3)	C38—N2—C39—C40	−105.0 (3)
C11—C12—C13—C14	64.9 (4)	N2—C39—C40—C41	−117.8 (3)
O6—C13—C14—C15	37.4 (3)	N2—C39—C40—C45	61.6 (4)
C12—C13—C14—C15	157.5 (3)	C45—C40—C41—C42	0.6 (5)
C13—C14—C15—C16	−28.2 (3)	C39—C40—C41—C42	−180.0 (3)
C14—C15—C16—O6	9.2 (3)	C40—C41—C42—C43	−0.6 (5)
C14—C15—C16—C30	127.4 (3)	C41—C42—C43—C44	0.4 (5)
C14—C15—C16—C17	−106.2 (3)	C42—C43—C44—C45	−0.2 (5)
C14—C13—O6—C16	−33.1 (3)	C43—C44—C45—C40	0.2 (5)

C12—C13—O6—C16	−158.9 (2)	C41—C40—C45—C44	−0.4 (5)
C30—C16—O6—C13	−107.3 (3)	C39—C40—C45—C44	−179.8 (3)

Hydrogen-bond geometry (Å, º)

D—H···*A*	*D*—H	H···*A*	*D*···*A*	*D*—H···*A*
O3—H3*A*···O5	0.84	2.10	2.798 (3)	140
O9—H9···O3	0.84	1.88	2.697 (3)	164
