# Supplementary material for: Access to Cyclic Monensin Derivatives via a Four-Component Ugi Reaction
Source: J Org Chem. 2026 Jul 4;91(28):9933–9. doi: 10.1021/acs.joc.6c01246 (PMC13386529; doi:10.1021/acs.joc.6c01246)
Supplement: Supplementary file 2 [file jo6c01246_si_002.pdf]

Supplementary material

Access to Cyclic Monensin Derivatives via a Four-Component Ugi Reaction

Robert Graniczny [a], Adam Huczyński [a], Jan Janczak [b], Dagmara Kłopotowska [c], Joanna Wietrzyk [c], Michał Sulik [a,*]

[a] *Department of Medical Chemistry, Faculty of Chemistry, Adam Mickiewicz University, Uniwersytetu Poznańskiego 8, 61–614 Poznań, Poland*

[b] *Hirszfeld Institute of Immunology and Experimental Therapy, Polish Academy of Sciences, Rudolfa Weigla 12, 53–114 Wrocław, Poland*

[c] *Institute of Low Temperature and Structure Research, Polish Academy of Sciences, Okólna 2, Wrocław, 50–422, Poland*

Index

Spectroscopic and spectrometric analysis of newly synthesized compounds S2
X-ray measurements S9
Literature S15

Spectroscopic and spectrometric analysis of newly synthesized compounds

List of spectra

Figure S1. The $^{13}C\{^{1}H\}$ NMR (151 MHz) spectrum of **3** in dichloromethane-d2. ....S3

Figure S2. The ^{1}H NMR (600 MHz) spectrum of **3** in dichloromethane-d2..S3

Figure S3. The $^{13}C\{^{1}H\}$ NMR (151 MHz) spectrum of **4** in dichloromethane-d2. ....S4

Figure S4. The ^{1}H NMR (600 MHz) spectrum of **4** in dichloromethane-d2..S4

Figure S5. The $^{13}C\{^{1}H\}$ NMR (151 MHz) spectrum of **5** in dichloromethane-d2. ....S5

Figure S6. The ^{1}H NMR (600 MHz) spectrum of **5** in dichloromethane-d2..S5

Figure S7. The $^{13}C\{^{1}H\}$ NMR (151 MHz) spectrum of **6** in dichloromethane-d2. ....S6

Figure S8. The ^{1}H NMR (600 MHz) spectrum of **6** in dichloromethane-d2..S6

Figure S9. The FT-IR spectrum of crystals of **3** recorded in KBr pellet in the range of 4000-500 cm^{-1}. S7

Figure S10. The FT-IR spectrum of crystals of **4** recorded in KBr pellet in the range of 4000-500 cm^{-1}. S7

Figure S11. The FT-IR spectrum of crystals of **5** recorded in KBr pellet in the range of 4000-500 cm^{-1}. S8

Figure S12. The FT-IR spectrum of crystals of **6** recorded in KBr pellet in the range of 4000-500 cm^{-1}. S8

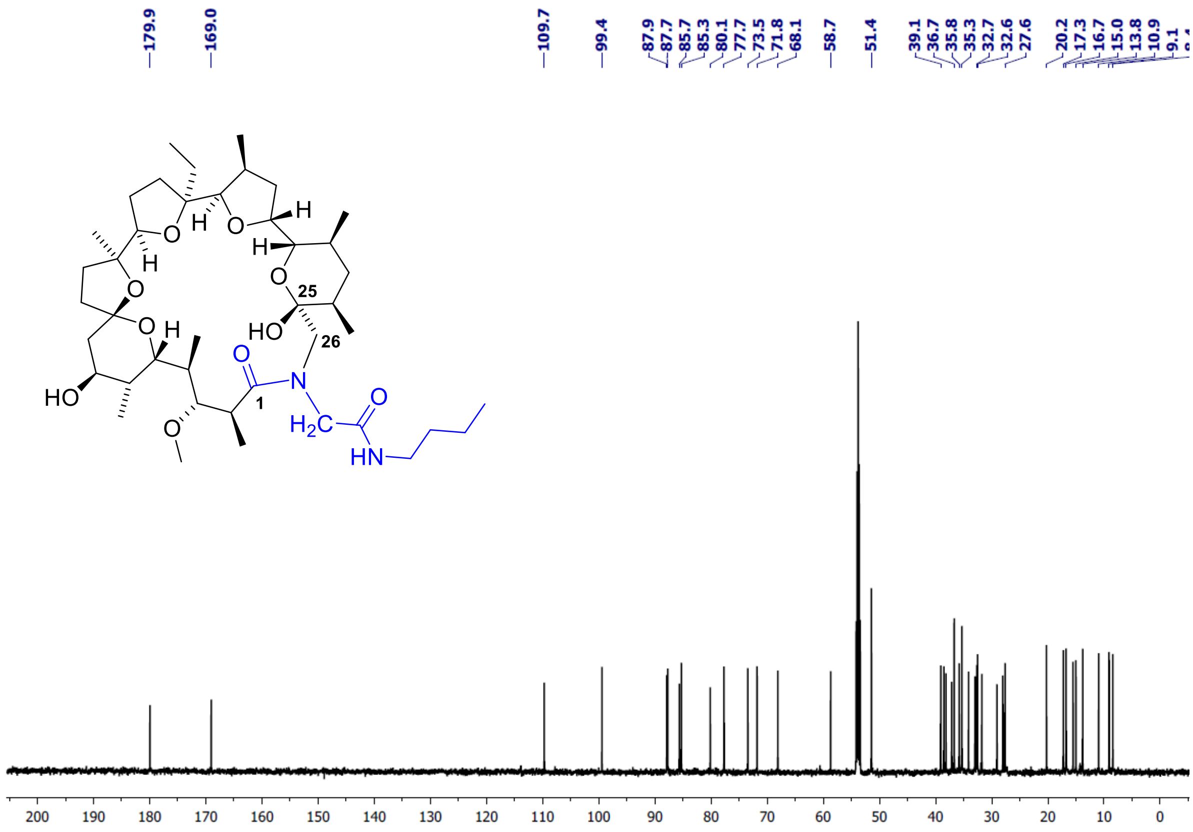

Figure S1. The $^{13}C\{^1H\}$ NMR (151 MHz) spectrum of **3** in dichloromethane-d2.

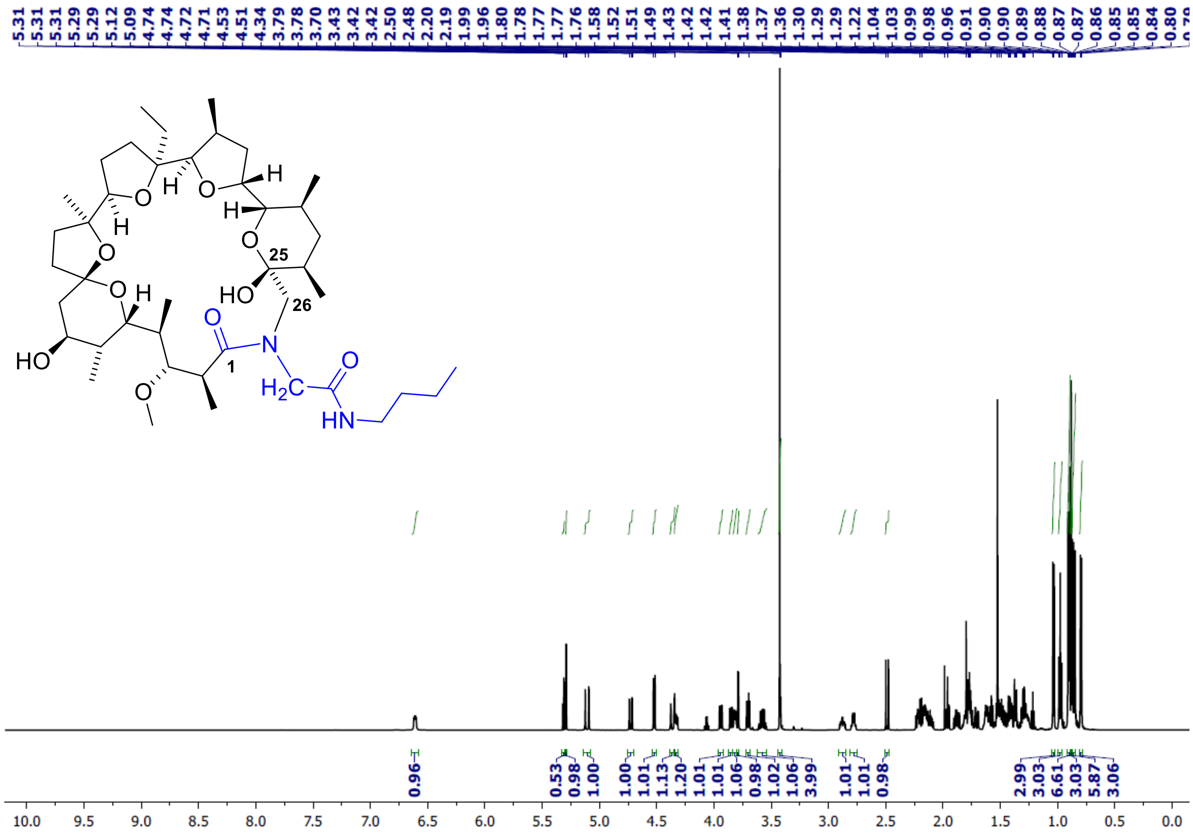

Figure S2. The ^1H NMR (600 MHz) spectrum of **3** in dichloromethane-d2.

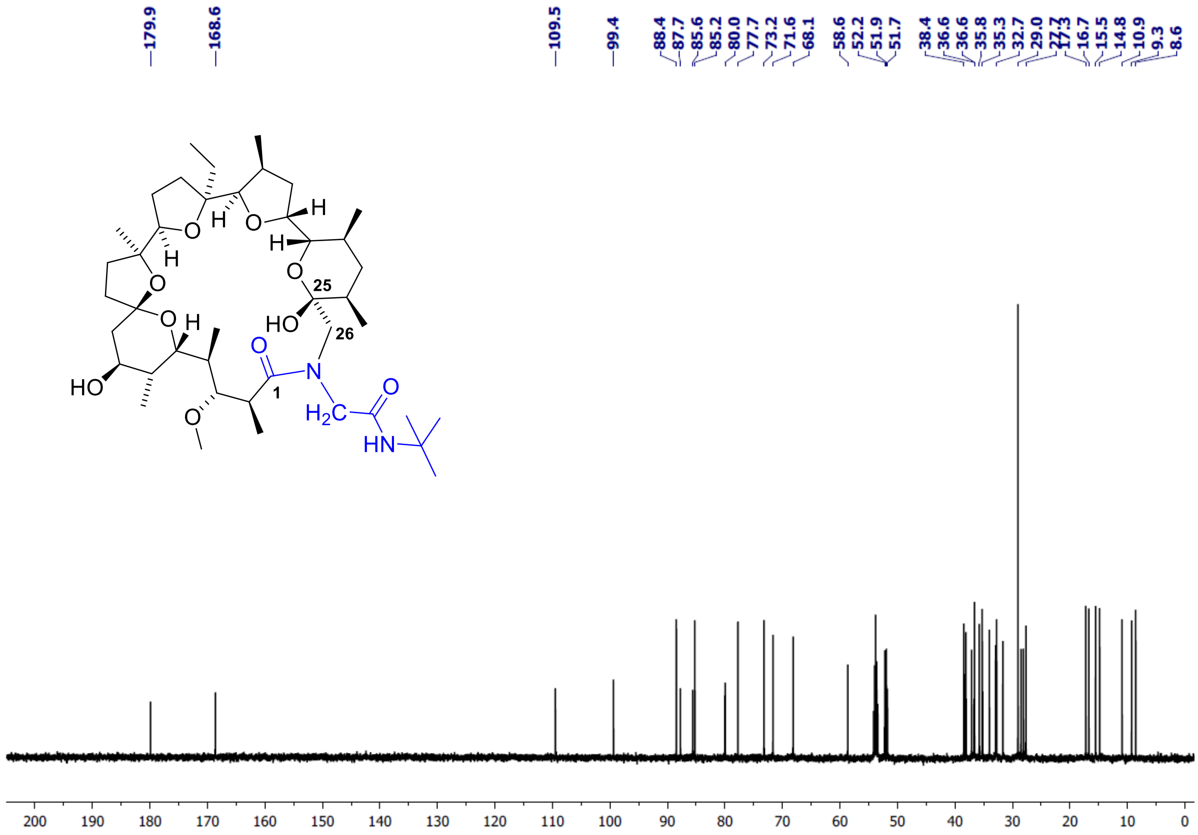

Figure S3. The $^{13}C\{^1H\}$ NMR (151 MHz) spectrum of **4** in dichloromethane-d2.

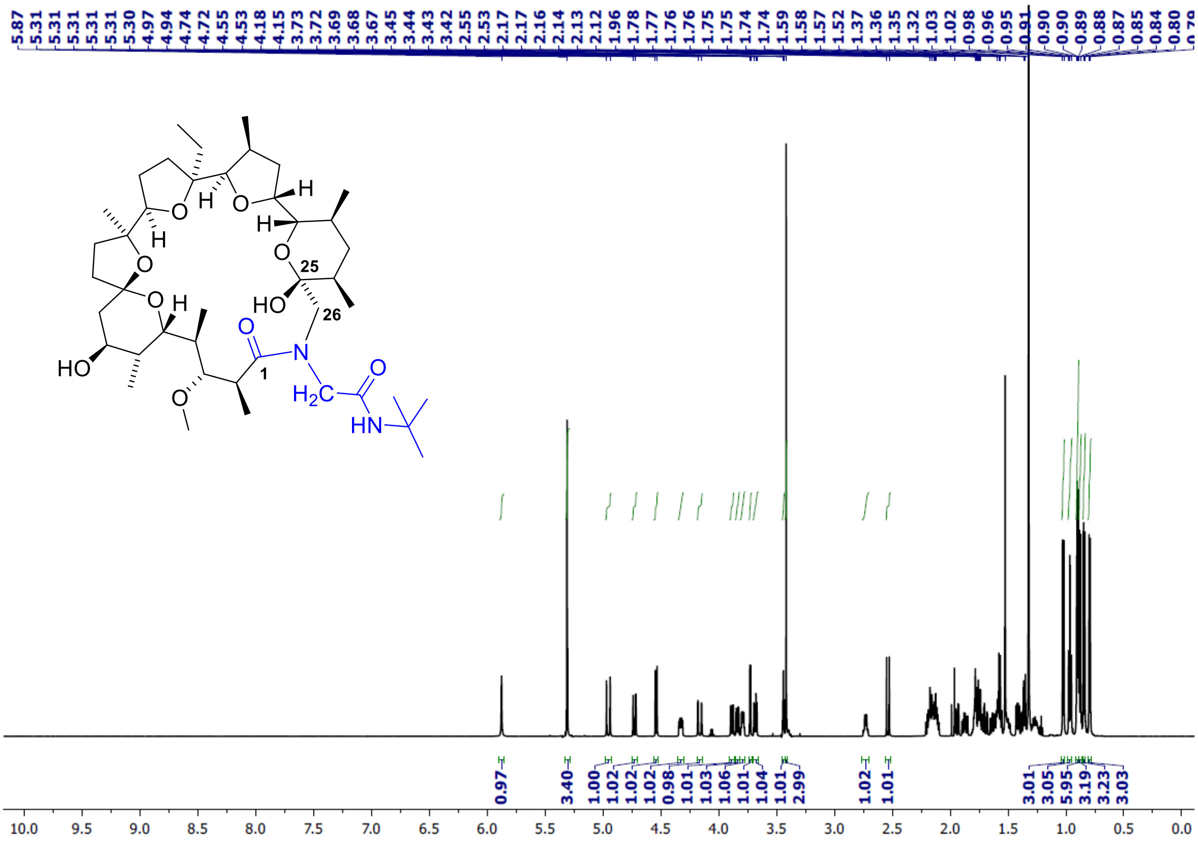

Figure S4. The ^1H NMR (600 MHz) spectrum of **4** in dichloromethane-d2.

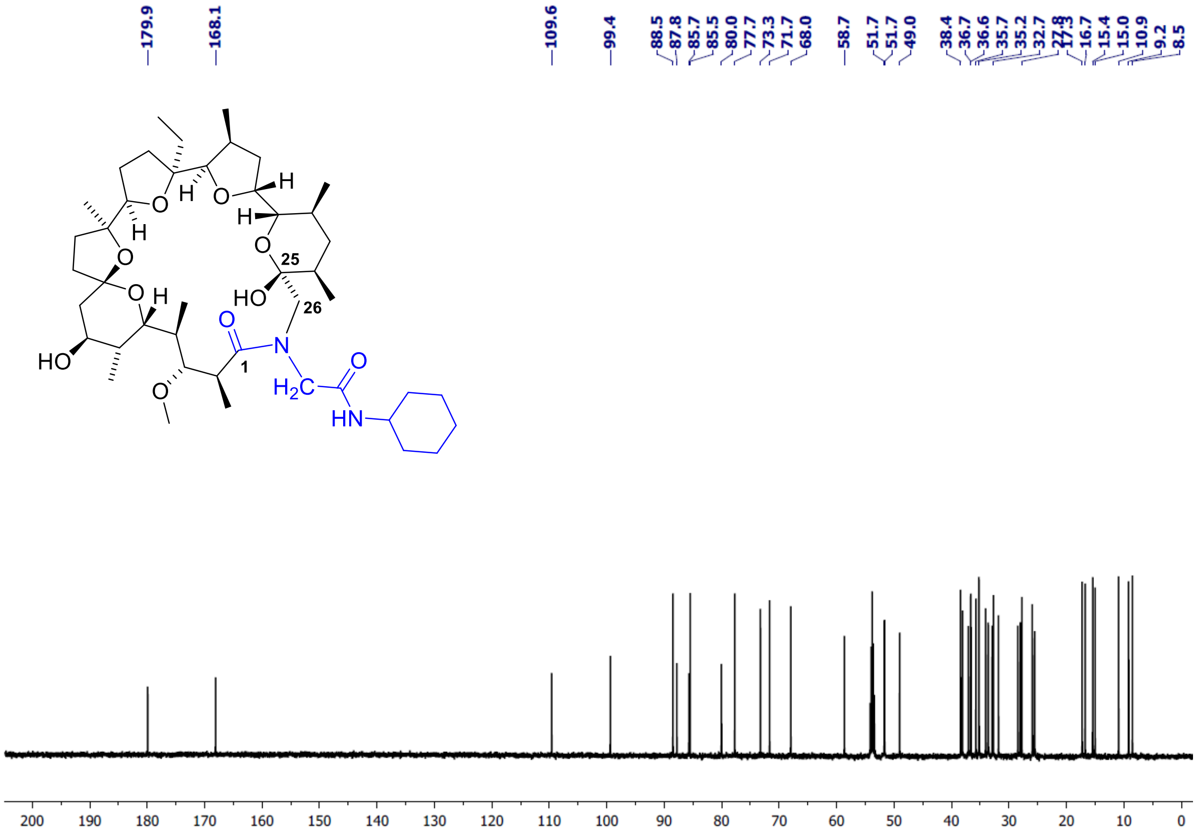

Figure S5. The $^{13}C\{^{1}H\}$ NMR (151 MHz) spectrum of **5** in dichloromethane-d2.

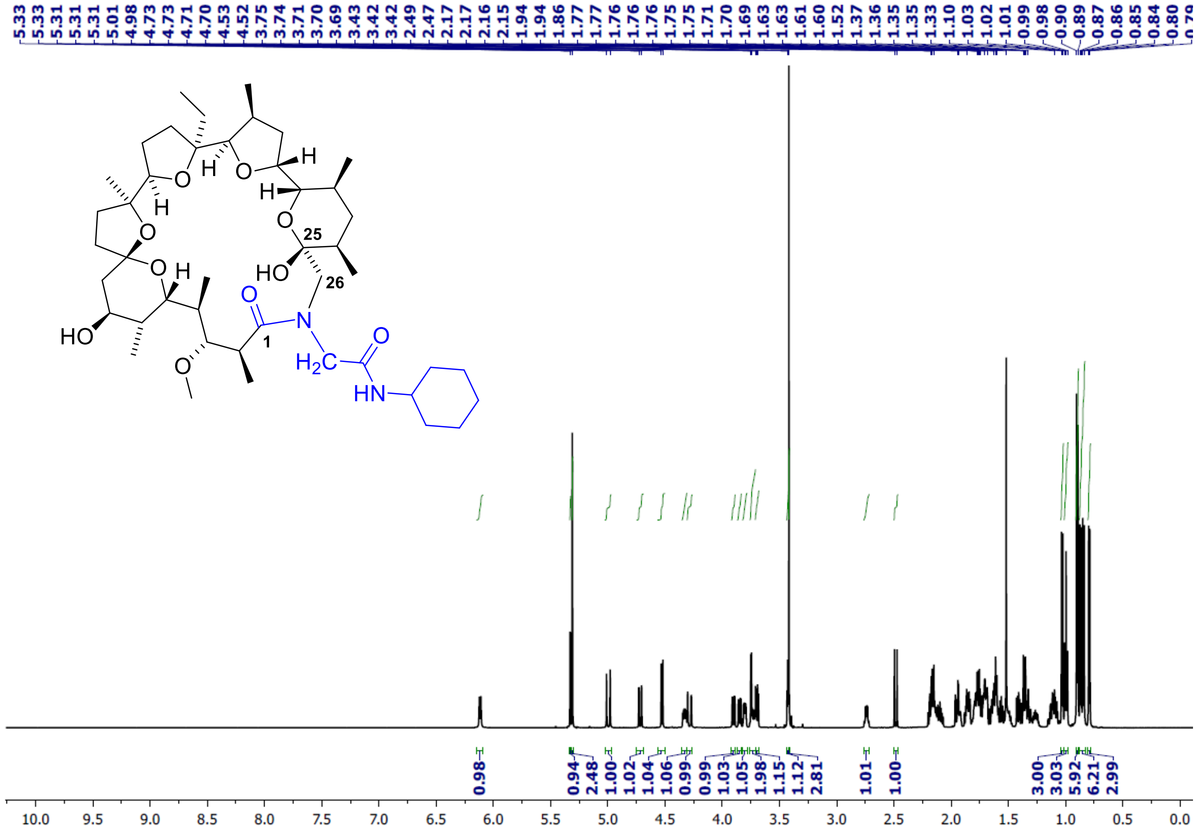

Figure S6. The ^{1}H NMR (600 MHz) spectrum of **5** in dichloromethane-d2.

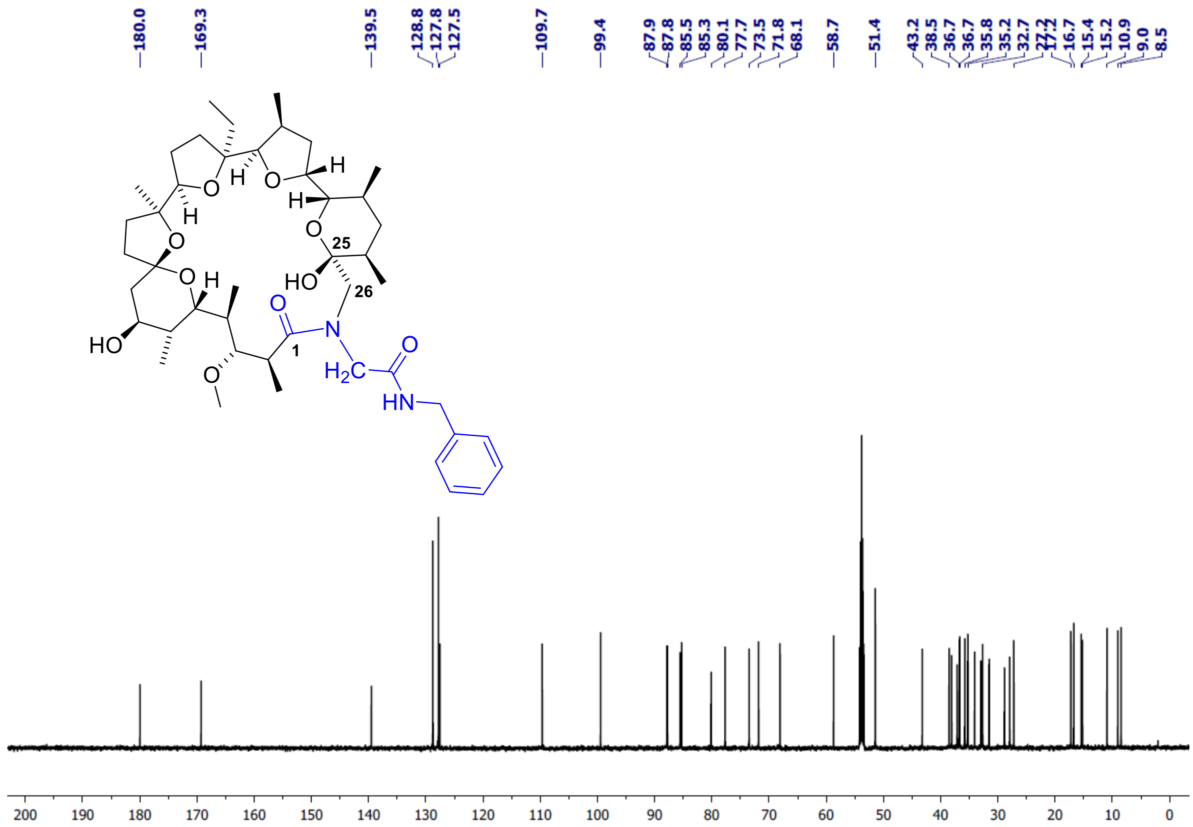

Figure S7. The $^{13}C\{^{1}H\}$ NMR (151 MHz) spectrum of **6** in dichloromethane-d2.

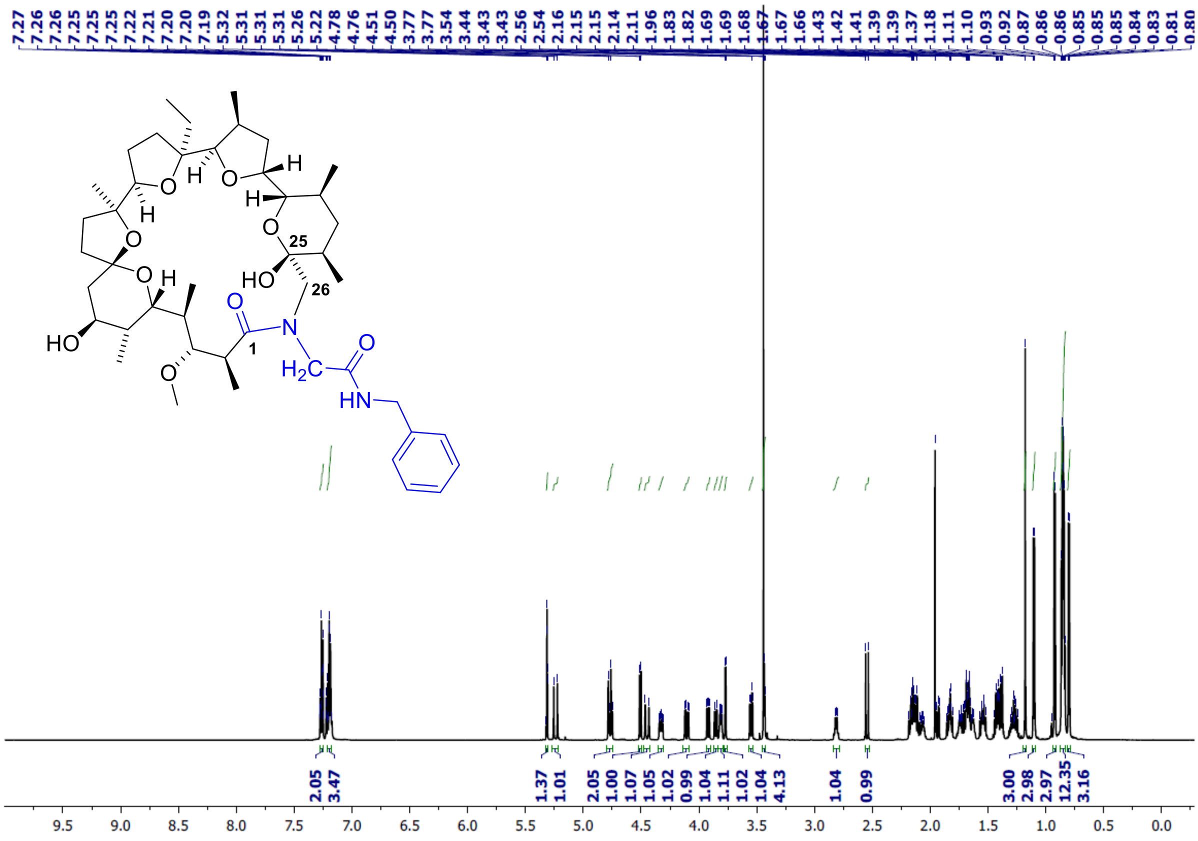

Figure S8. The ^{1}H NMR (600 MHz) spectrum of **6** in dichloromethane-d2.

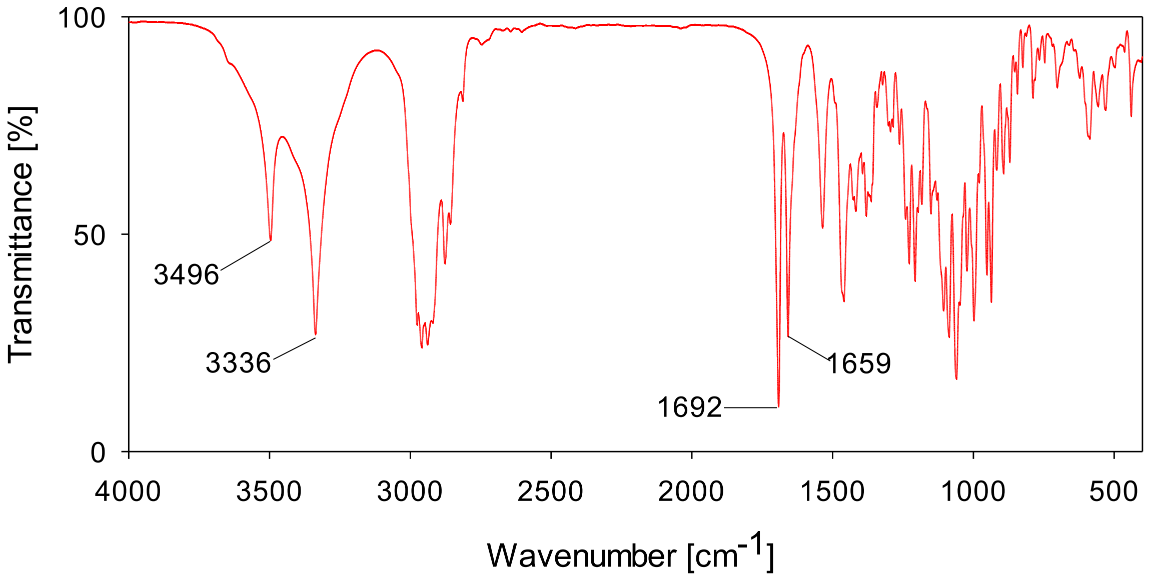

Figure S9. The FT-IR spectrum of crystals of **3** recorded in KBr pellet in the range of 4000-500 cm^{-1}.

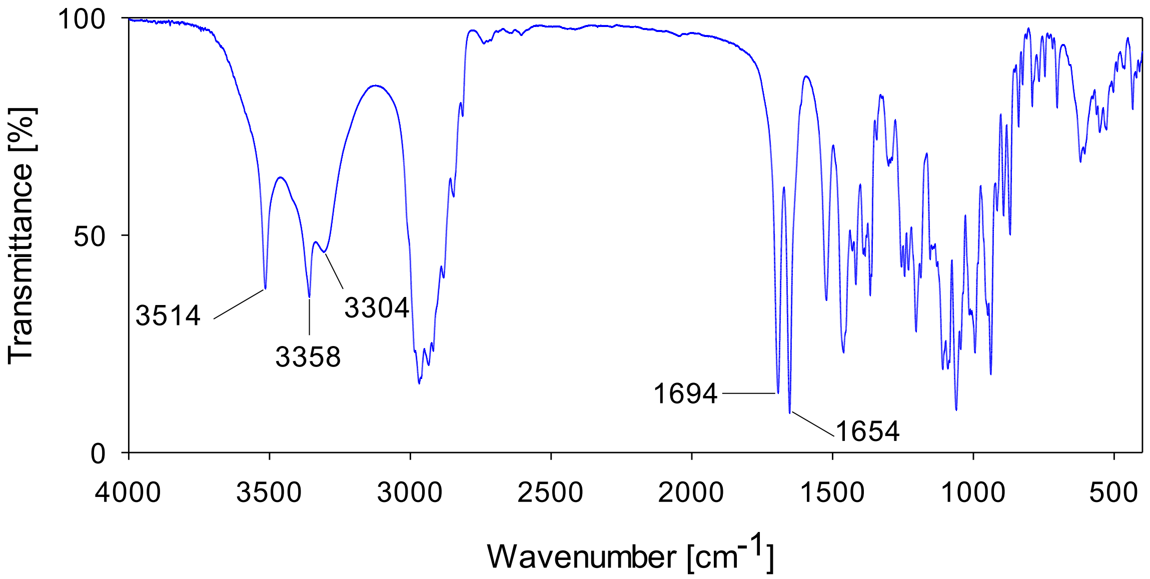

Figure S10. The FT-IR spectrum of crystals of **4** recorded in KBr pellet in the range of 4000-500 cm^{-1}.

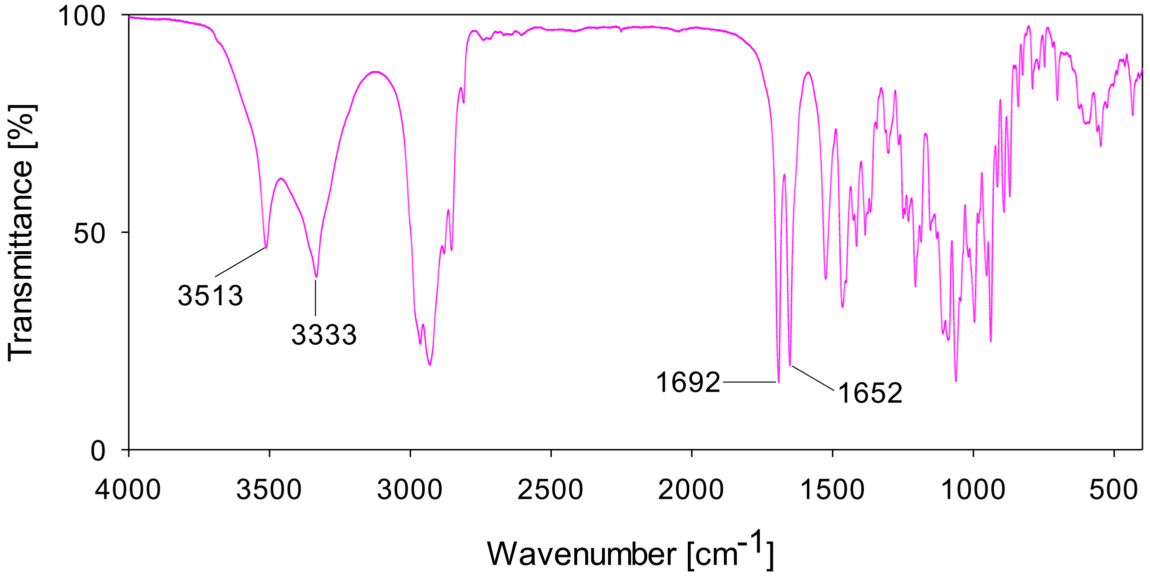

Figure S11. The FT-IR spectrum of crystals of **5** recorded in KBr pellet in the range of 4000-500 cm^{-1}.

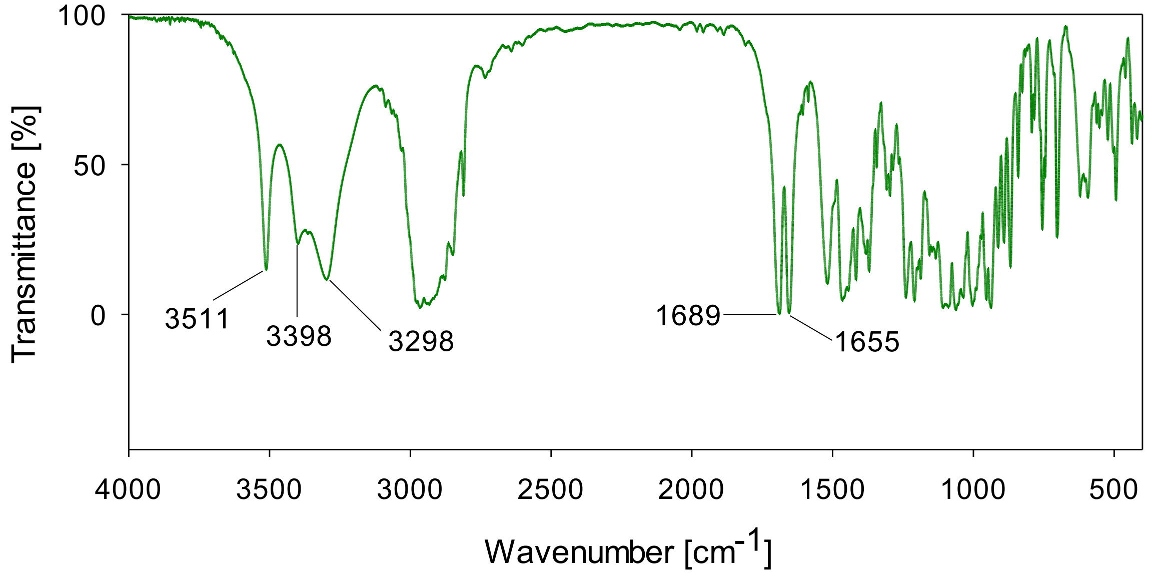

Figure S12. The FT-IR spectrum of crystals of **6** recorded in KBr pellet in the range of 4000-500 cm^{-1}.

X-ray measurements

The X-ray intensity data for compounds **3**–**6** were collected using graphite monochromatic Mo k$_\alpha$ radiation on a four-circle κ geometry Xcalibur diffractometer with Sapphire2 area CCD detector. Data collections were made using the CrysAlisPro 1.171.42.93a. Integration, scaling of the reflections, corrections for Lorenz and polarization effects and absorption corrections were performed using the CrysAlisPro 1.171.42.93a program.[1] The structures were solved by the direct methods using SHELXT-2014/7 [2] and refined using SHELXL-2018/3 program.[3] The hydrogen atoms linked to carbon atoms were introduced in their geometrical positions and treated as rigid. The H atoms involved in the hydrogen bonds were refined if they gave reasonable hydrogen bonds, otherwise they were constrained. The final difference Fourier maps showed no peaks of chemical significance. Details of the data collection parameters, crystallographic data and final agreement parameters are collected in Table S1. The hydrogen bonds are summarized in Table S2–S5. Structure visualizations were made with the Diamond 3.0.[4]

Table S1. Crystal data, collection data and structure refinement parameters for compounds **3**–**6**.

		**3**	**4**	**5**	**6**
Formula		$C_{42}H_{72}N_2O_{10}$	$C_{42}H_{72}N_2O_{10}$	$C_{44}H_{74}N_2O_{10}$	$C_{45}H_{70}N_2O_{10}$
Molecular weight		765.01	765.01	791.05	799.03
Temperature (K)		100	100	295	100
Crystal system		Monoclinic	Monoclinic	Orthorhombic	Monoclinic
Space group		$P2_1$	$P2_1$	$P2_12_12_1$	$P2_1$
Unit cell dimensions	*a* (Å)	13.7694 (11)	13.5682 (18)	10.6322 (5)	12.5180 (3)
	b (Å)	11.5488 (4)	11.2722 (7)	11.7204 (6)	11.3789 (3)
	c (Å)	14.8152 (11)	15.2350 (14)	36.0543 (18)	15.0822 (4)
	β (°)	117.327 (10)	109.469 (13)	–	96.558 (2)
V (Å^3)		2093.0 (3)	2196.9 (4)	4492.9 (4)	2134.27 (10)
Z		2	2	4	2
F(000)		836	836	1728	868
D_{cal} (g·cm^{-3})		1.214	1.156	1.169	1.243
θ range (°)		2.7–27.0°	2.5–27.5°	3.1–27.5°	2.4–29.3°
μ (mm^{-1})		0.09	0.08	0.08	0.09
Crystal size (mm)		0.33 × 0.31 × 0.24	0.29 × 0.25 × 0.15	0.32 × 0.24 × 0.21	0.35 × 0.29 × 0.24
T_{min} / T_{max}		0.985/1.000	0.988/1.000	0.990/1.000	0.988/1.000
Total / unique / obs refls		22652/8345/6338	20268/9716/4595	34323/10944/5434	42363/10554/7646
R_{int}		0.041	0.071	0.048	0.070

R $[F^2>2\sigma(F^2)]$	0.051	0.078	0.057	0.056
wR [F^2 all refls]	0.092	0.124	0.107	0.110
S	1.00	1.00	1.00	1.01
Flack parameter	0.6(5)	–0.2 (10)	–0.3 (5)	0.5 (5)
$\Delta\rho_{max}$, $\Delta\rho_{min}$ (eÅ^{-3})	0.22, −0.27	0.25, –0.29	0.13, –0.13	0.22, –0.25

Table S2. Hydrogen bond geometry for **3** (Å, º).

D*—H···*A	***D*—H**	**H···*A***	***D*···*A***	***D*—H···*A***
O3—H3*O*···O5	0.84	2.11	2.798 (3)	139
O9—H91···O3	0.84	1.92	2.736 (3)	164
N2—H2*N*···O6	0.93 (4)	2.19 (4)	3.119 (4)	178 (3)

Table S3. Hydrogen bond geometry for **4** (Å, º).

D*—H···*A	***D*—H**	**H···*A***	***D*···*A***	***D*—H···*A***
O3—H3*O*···O5	0.84	2.11	2.796 (6)	139
O9—H9*O*···O3	0.84	1.93	2.746 (5)	164
N2—H2*N*···O6	0.86	2.45	3.300 (6)	170

Table S4. Hydrogen bond geometry for **5** (Å, º).

D*—H···*A	***D*—H**	**H···*A***	***D*···*A***	***D*—H···*A***
O3—H3···O5	0.82	2.12	2.787 (3)	139
O9—H91···O3	0.82	2.06	2.870 (3)	169
N2—H2···O6	0.85 (3)	2.55 (3)	3.385 (4)	168 (3)

Table S5. Hydrogen bond geometry for **6** (Å, º).

D*—H···*A	***D*—H**	**H···*A***	***D*···*A***	***D*—H···*A***
O3—H3*A*···O5	0.84	2.10	2.798 (3)	140
O9—H9···O3	0.84	1.88	2.697 (3)	164

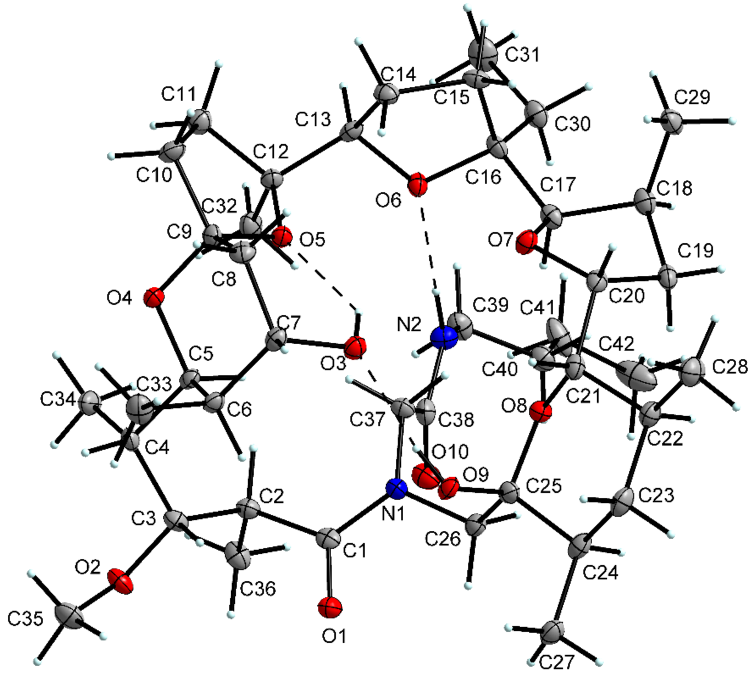

Figure S13. The crystal structure of compound **3**. Displacement ellipsoids are shown at the 40% probability level, H atoms as arbitrary radii.

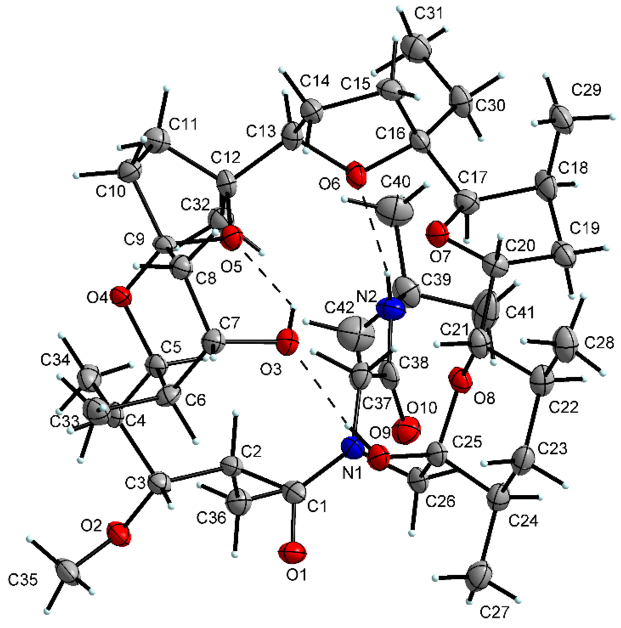

Figure S14. The crystal structure of compound **4**. Displacement ellipsoids are shown at the 40% probability level, H atoms as arbitrary radii.

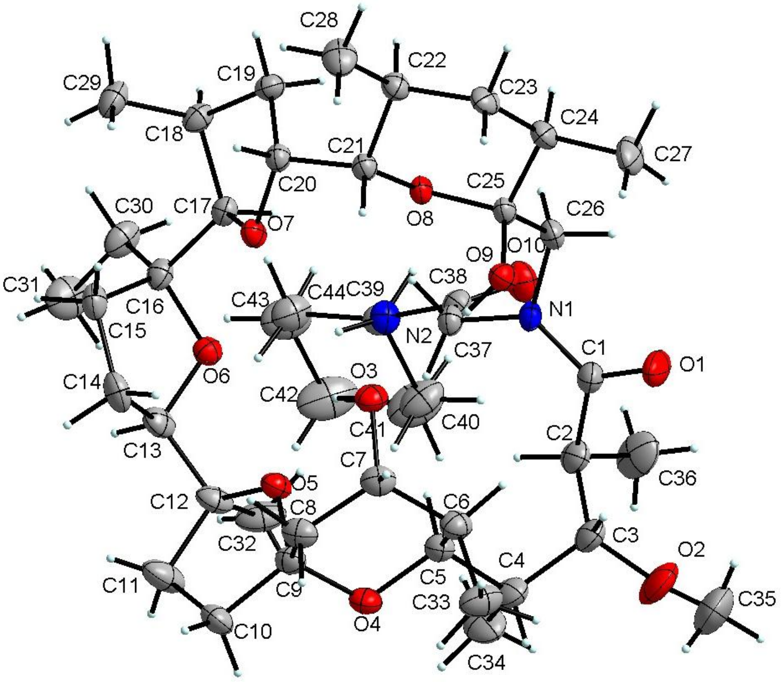

Figure S15. The crystal structure of compound **5**. Displacement ellipsoids are shown at the 40% probability level, H atoms as arbitrary radii.

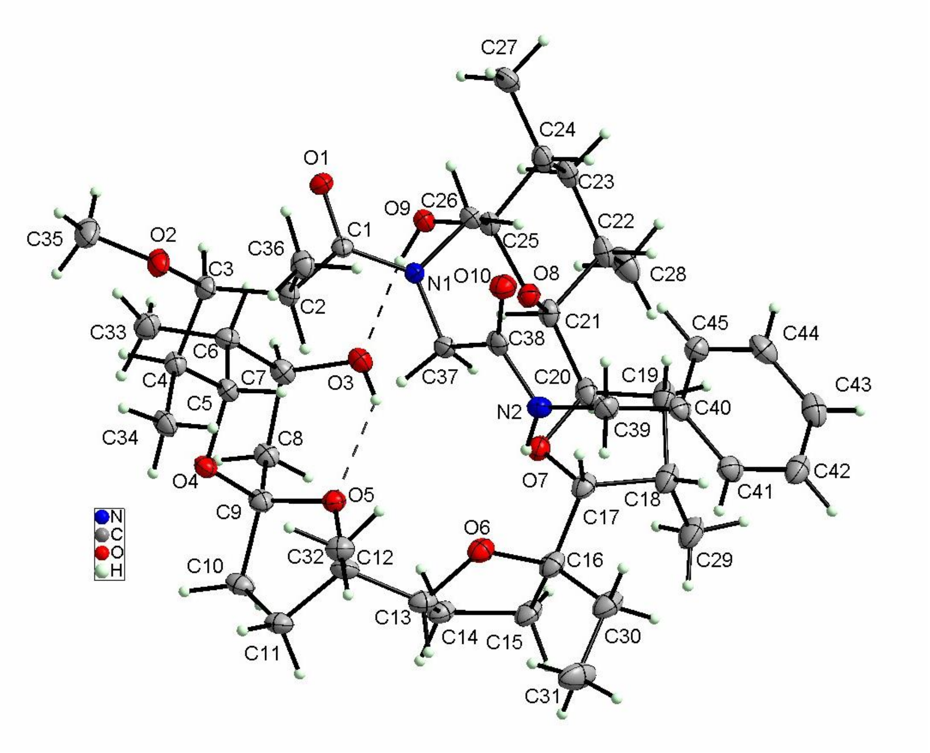

Figure S16. The crystal structure of compound **6**. Displacement ellipsoids are shown at the 40% probability level, H atoms as arbitrary radii.

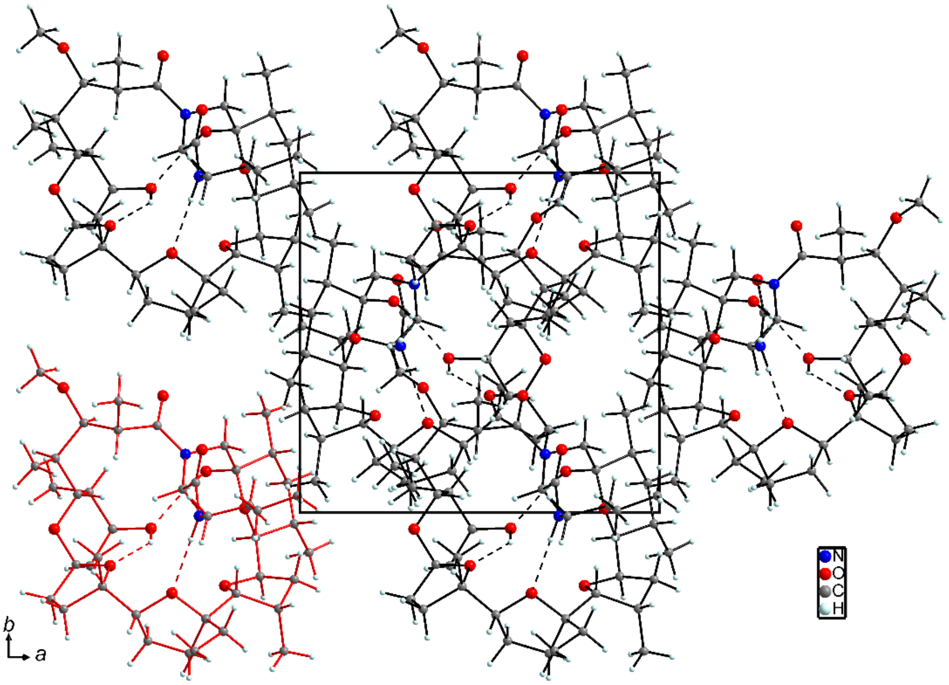

Figure S17. Packing along for **3**.

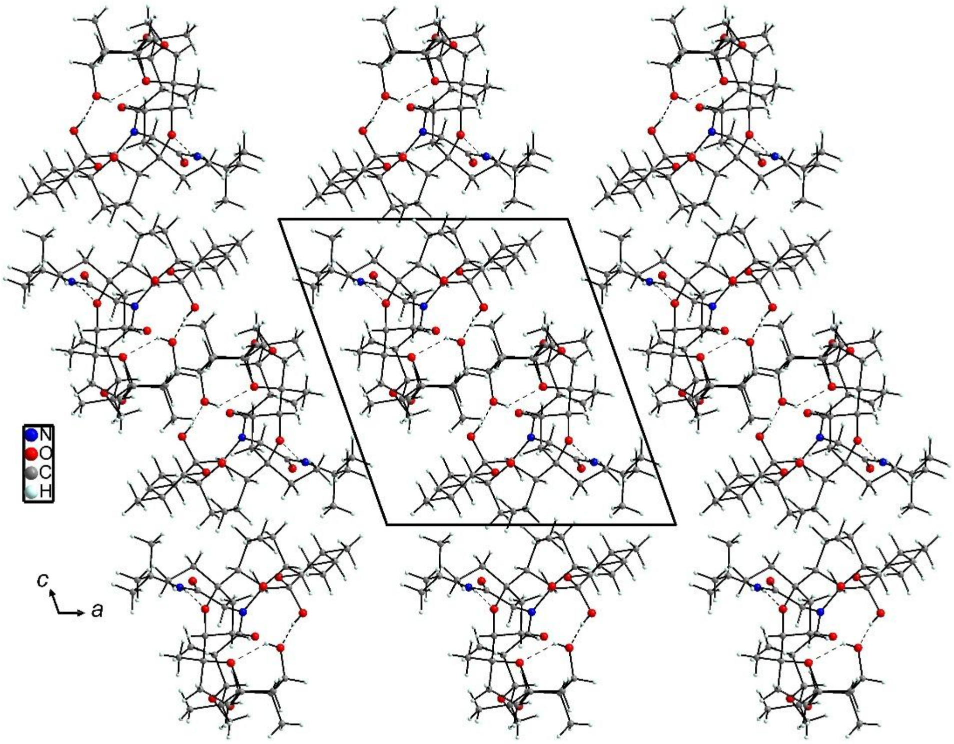

Figure S18. Packing along for **4**.

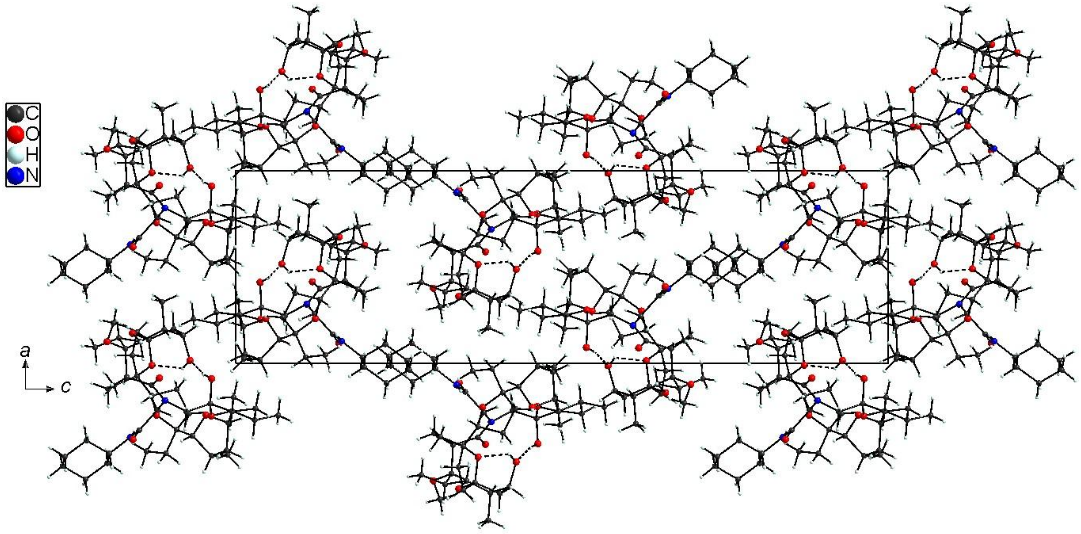

Figure S19. Packing along for **5**.

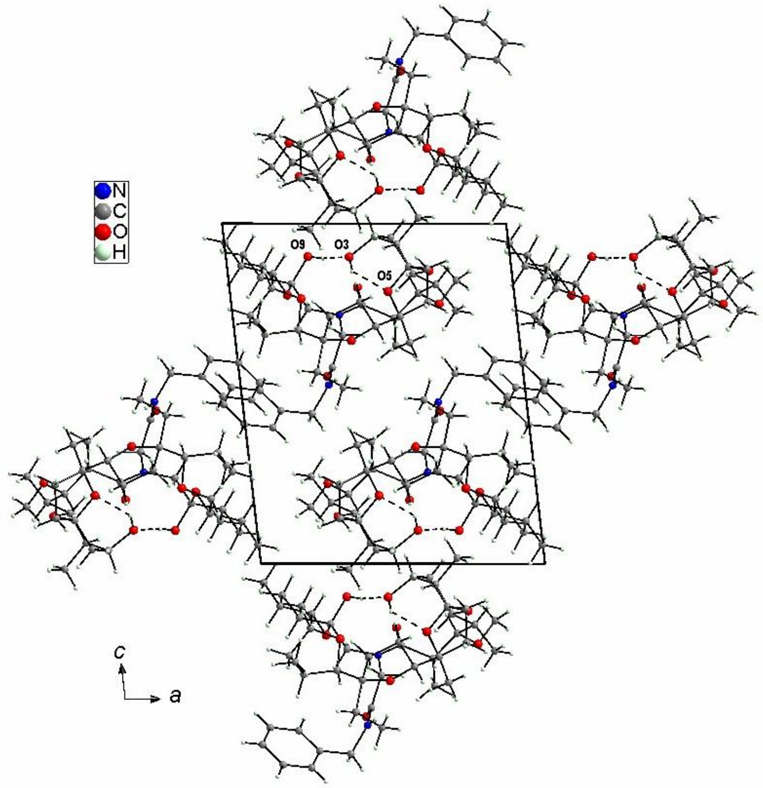

Figure S20. Packing along for **6**.

Literature

(1) Rigaku Oxford Diffraction, CrysAlisPro 1.171.42.93a Software system, Oxford, UK,2023. https://rigaku.com/products/crystallography/x-ray-diffraction/crysalispro.

(2) Sheldrick, G. M. SHELXT – Integrated Space-Group and Crystal-Structure Determination. *Acta Crystallogr. A Found. Adv.* **2015**, *71* (1), 3–8. https://doi.org/10.1107/S2053273314026370.

(3) Sheldrick, G. M. Crystal Structure Refinement with SHELXL. *Acta Crystallogr. C Struct. Chem.* **2015**, *71* (1), 3–8. https://doi.org/10.1107/S2053229614024218.

(4) Brandenburg, K.; Putz, H. DIAMOND Version 3.0. Crystal Impact GbR, Bonn, Germany 2006.
