# Supplementary material for: Access to Cyclic Monensin Derivatives via a Four-Component Ugi Reaction
Source: J Org Chem. 2026 Jul 4;91(28):9933–9. doi: 10.1021/acs.joc.6c01246 (PMC13386529; doi:10.1021/acs.joc.6c01246)

checkCIF/PLATON report

Structure factors have been supplied for datablock(s) monugi8_100

THIS REPORT IS FOR GUIDANCE ONLY. IF USED AS PART OF A REVIEW PROCEDURE FOR PUBLICATION, IT SHOULD NOT REPLACE THE EXPERTISE OF AN EXPERIENCED CRYSTALLOGRAPHIC REFEREE.

No syntax errors found. CIF dictionary Interpreting this report

Datablock: monugi8_100

Bond precision: C-C = 0.0089 A Wavelength=0.71073

Cell:	a=13.5682(18)	b=11.2722(7)	c=15.2350(14)
	alpha=90	beta=109.469(13)	gamma=90
Temperature:	100 K		

	Calculated	Reported
Volume	2196.9(4)	2196.9(4)
Space group	P 21	P 21
Hall group	P 2yb	P 2yb
Moiety formula	C42 H72 N2 O10	C42 H72 N2 O10
Sum formula	C42 H72 N2 O10	C42 H72 N2 O10
Mr	765.02	765.01
Dx,g cm-3	1.156	1.156
Z	2	2
Mu (mm-1)	0.081	0.081
F000	836.0	836.0
F000'	836.41	
h,k,lmax	17,14,19	17,14,19
Nref	10095[5306]	9716
Tmin,Tmax	0.977,0.988	0.988,1.000
Tmin'	0.977	

Correction method= # Reported T Limits: Tmin=0.988 Tmax=1.000
AbsCorr = MULTI-SCAN

Data completeness= 1.83/0.96 Theta(max)= 27.497

R(reflections)= 0.0777(4595) wR2(reflections)= 0.1240(9716)

S = 1.000 Npar= 504

The following ALERTS were generated. Each ALERT has the format
test-name_ALERT_alert-type_alert-level.
Click on the hyperlinks for more details of the test.

Alert level B

```
PLAT601_ALERT_2_B Unit-Cell Contains Solvent Accessible VOIDS .LE.        196 Ang**3
```

Author Response: In our opinion there is no solvent, we checked with the SQUEEZE procedure and it shows only trace electron density

---

Alert level C

```
STRVA01_ALERT_4_C           Flack test results are meaningless.
           From the CIF: _refine_ls_abs_structure_Flack   -0.200
           From the CIF: _refine_ls_abs_structure_Flack_su    1.000
PLAT026_ALERT_3_C Ratio Observed / Unique Reflections (too) Low ..        47% Check
PLAT340_ALERT_3_C Low Bond Precision on  C-C Bonds ..............    0.00887 Ang.
PLAT410_ALERT_2_C Short Intra H...H Contact  H2       ..H37B     .       1.96 Ang.
                                                     x,y,z  =      1_555 Check
PLAT906_ALERT_3_C Large K Value in the Analysis of Variance ......      5.866 Check
PLAT906_ALERT_3_C Large K Value in the Analysis of Variance ......      2.279 Check
PLAT910_ALERT_3_C Missing FCF Reflection(s) Below Theta(Min)[Deg]=       2.46 Note
                1  0  0,   1  1  0,  -1  0  1,   0  0  1,   0  1  1,
```

Alert level G

```
PLAT002_ALERT_2_G Number of Distance or Angle Restraints on AtSite          2 Note
PLAT007_ALERT_5_G Number of Unrefined Donor-H Atoms ..............          2 Report
              H3O     H9O
PLAT032_ALERT_4_G Std. Uncertainty on Flack Parameter Value High .      1.000 Report
PLAT172_ALERT_4_G The CIF-Embedded .res File Contains DFIX Records          1 Report
PLAT398_ALERT_2_G Deviating  C-O-C    Angle From 120 for O6       .      109.2 Degree
PLAT398_ALERT_2_G Deviating  C-O-C    Angle From 120 for O7       .      109.0 Degree
PLAT791_ALERT_4_G Model has Chirality at C2          (Sohncke SpGr)          S Verify
PLAT791_ALERT_4_G Model has Chirality at C3          (Sohncke SpGr)          R Verify
PLAT791_ALERT_4_G Model has Chirality at C4          (Sohncke SpGr)          S Verify
PLAT791_ALERT_4_G Model has Chirality at C5          (Sohncke SpGr)          S Verify
PLAT791_ALERT_4_G Model has Chirality at C6          (Sohncke SpGr)          R Verify
PLAT791_ALERT_4_G Model has Chirality at C7          (Sohncke SpGr)          S Verify
PLAT791_ALERT_4_G Model has Chirality at C9          (Sohncke SpGr)          R Verify
PLAT791_ALERT_4_G Model has Chirality at C12         (Sohncke SpGr)          S Verify
PLAT791_ALERT_4_G Model has Chirality at C13         (Sohncke SpGr)          R Verify
PLAT791_ALERT_4_G Model has Chirality at C16         (Sohncke SpGr)          S Verify
PLAT791_ALERT_4_G Model has Chirality at C17         (Sohncke SpGr)          R Verify
```

```
PLAT791_ALERT_4_G Model has Chirality at C18       (Sohncke SpGr)          S Verify
PLAT791_ALERT_4_G Model has Chirality at C20       (Sohncke SpGr)          R Verify
PLAT791_ALERT_4_G Model has Chirality at C21       (Sohncke SpGr)          S Verify
PLAT791_ALERT_4_G Model has Chirality at C22       (Sohncke SpGr)          S Verify
PLAT791_ALERT_4_G Model has Chirality at C24       (Sohncke SpGr)          R Verify
PLAT791_ALERT_4_G Model has Chirality at C25       (Sohncke SpGr)          R Verify
PLAT860_ALERT_3_G Number of Least-Squares Restraints ............          2 Note
PLAT899_ALERT_4_G SHELXL2018 is Outdated   and Succeeded by SHELXL     2019/3 Note
PLAT912_ALERT_4_G Missing # of FCF Reflections Above STh/L=  0.600         62 Note
PLAT913_ALERT_3_G Missing # of Very Strong Reflections in FCF ....          1 Note
                1  0  0,
PLAT941_ALERT_3_G Average HKL Measurement Multiplicity ..........        3.9 Low
PLAT961_ALERT_5_G Dataset Contains no Negative Intensities .......     Please Check
PLAT965_ALERT_2_G The SHELXL WEIGHT Optimisation has not Converged     Please Check
PLAT967_ALERT_5_G Note: Two-Theta Cutoff Value in Embedded .res ..       55.0 Degree
PLAT969_ALERT_5_G The 'Henn et al.' R-Factor-gap value ..........      1.112 Note
              Predicted wR2: Based on SigI**2 11.15 or SHELX Weight 12.41 Note
PLAT978_ALERT_2_G Number C-C Bonds with Positive Residual Density.          0 Info
```

```
   0 ALERT level A = Most likely a serious problem - resolve or explain
   1 ALERT level B = A potentially serious problem, consider carefully
   7 ALERT level C = Check. Ensure it is not caused by an omission or oversight
  33 ALERT level G = General information/check it is not something unexpected

   0 ALERT type 1 CIF construction/syntax error, inconsistent or missing data
   7 ALERT type 2 Indicator that the structure model may be wrong or deficient
   8 ALERT type 3 Indicator that the structure quality may be low
  22 ALERT type 4 Improvement, methodology, query or suggestion
   4 ALERT type 5 Informative message, check
```

It is advisable to attempt to resolve as many as possible of the alerts in all categories. Often the minor alerts point to easily fixed oversights, errors and omissions in your CIF or refinement strategy, so attention to these fine details can be worthwhile. It is up to the individual to critically assess their own results and, if necessary, seek expert advice.

PLATON version of 23/04/2026; check.def file version of 30/03/2026

---

duplicate check

No duplication found

Datablock monugi8_100 - ellipsoid plot

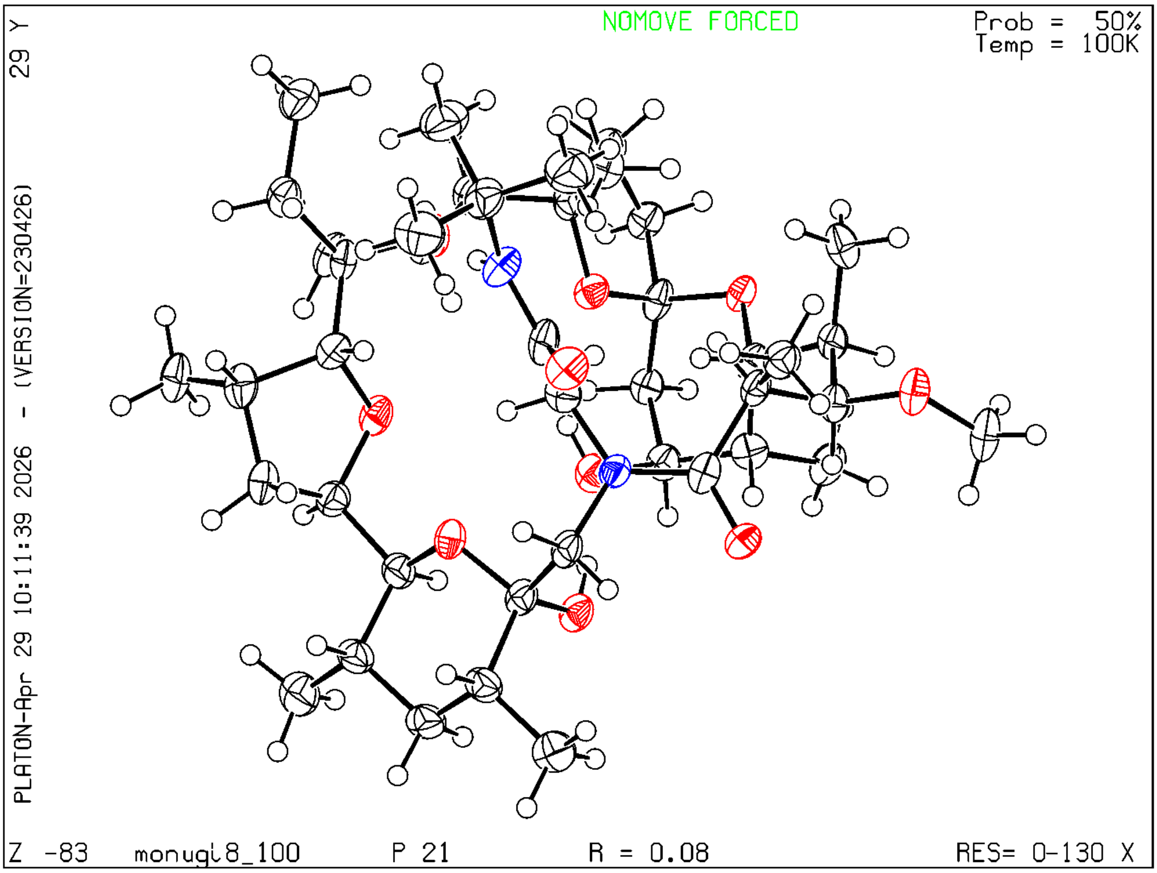

Supplement: Supplementary file 1 [file checkcif_UGI_8_4.pdf › Compouds data/Compound 4/scXRD/checkcif_UGI_8_4.pdf]
